# Exploring Species-Specificity in TLR4/MD-2 Inhibition with Amphiphilic Lipid A Mimicking Glycolipids

**DOI:** 10.3390/molecules28165948

**Published:** 2023-08-08

**Authors:** Alessio Borio, Aurora Holgado, Christina Passegger, Herbert Strobl, Rudi Beyaert, Holger Heine, Alla Zamyatina

**Affiliations:** 1 Institute of Organic Chemistry, Department of Chemistry, University of Natural Resources and Life Sciences, Muthgasse 18, 1190 Vienna, Austria; 2 Unit of Molecular Signal Transduction in Inflammation, VIB-UGent Center for Inflammation Research, Department for Biomedical Molecular Biology, Ghent University, Technologiepark 71, B-9052 Ghent, Belgium; 3 Division of Immunology and Pathophysiology, Medical University Graz, Heinrichstraße 31, 8010 Graz, Austria; 4 Research Group Innate Immunity, Priority Area Chronic Lung Diseases, Research Center Borstel, Leibniz Lung Center, Airway Research Center North (ARCN), German Center for Lung Research (DZL), Parkallee 22, 23845 Borstel, Germany

**Keywords:** carbohydrate-based inhibitors, lipopolysaccharide, inflammation, Toll-like receptor, carbohydrates

## Abstract

The Toll-like receptor 4 (TLR4)/myeloid differentiation factor 2 (MD-2) complex is a key receptor of the innate immune system and a major driver of inflammation that is responsible for the multifaceted defense response to Gram-negative infections. However, dysfunction in the tightly regulated mechanisms of TLR4-mediated signaling leads to the uncontrolled upregulation of local and systemic inflammation, often resulting in acute or chronic disease. Therefore, the TLR4/MD-2 receptor complex is an attractive target for the design and development of anti-inflammatory therapies which aim to control the unrestrained activation of TLR4-mediated signaling. Complex structure–activity relationships and species-specificity behind ligand recognition by the TLR4/MD-2 complex complicate the development of MD-2-specific TLR4 antagonists. The restriction of the conformational flexibility of the disaccharide polar head group is one of the key structural features of the newly developed lipid A—mimicking glycophospholipids, which are potential inhibitors of TLR4-mediated inflammation. Since phosphorylation has a crucial influence on MD-2–ligand interaction, glycolipids with variable numbers and positioning of phosphate groups were synthesized and evaluated for their ability to inhibit TLR4-mediated pro-inflammatory signaling in human and murine immune cells. A bis-phosphorylated glycolipid was found to have nanomolar antagonist activity on human TLR4 while acting as a partial agonist on murine TLR4. The glycolipid inhibited mTLR4/MD-2-mediated cytokine release, acting as an antagonist in the presence of lipopolysaccharide (LPS), but at the same time induced low-level cytokine production.

## 1. Introduction

The Toll-like Receptor 4 (TLR4)/myeloid differentiation 2 (MD-2) complex is a typical pattern recognition receptor (PRR) of the innate immune system that is responsible for the rapid recognition of pathogen-associated molecular patterns (PAMPs), and this early recognition event is critical for the initiation of beneficial innate immune responses such as inflammation [1,2]. TLR4 is the major driver of pro-inflammatory response against Gram-negative infection but is also known to be a mediator of oxidative stress-driven [3,4] and viral-protein-induced [5,6] inflammation. In general, an inflammatory response triggered by TLR4 activation includes the downstream production of cytokines, prostaglandins, adhesion proteins, and reactive oxygen species, and is aims to facilitate natural healing in the immunocompetent host. However, dysfunction in the tightly coordinated mechanisms of TLR4-mediated signaling can result in the uncontrolled upregulation of local and systemic inflammatory responses, leading to acute or chronic diseases, the most devastating of which are sepsis syndrome and acute lung injury [7,8,9].

Over the past two decades, the downregulation of TLR4 signaling has been shown to be potentially beneficial in the treatment of arthritis [10], viral infection-related pathology [6,11], and sepsis syndrome [12,13]. Exposure to lipopolysaccharide (LPS) has been confirmed to play a central role in the development of allergies and asthma, and the LPS-sensing protein TLR4 has been investigated for its role as a critical contributor to asthma-related pathophysiology [14,15,16,17,18].

However, the development of therapies that are capable of controlling TLR4-mediated inflammation has stalled following the failure of the antisepsis drug candidate Eritoran to improve survival in sepsis patients in a Phase 3 clinical trial [19,20,21]. Thus, despite significant progress and advances in the development of anti-sepsis strategies, the medical options for lethal endotoxemia remain limited, and sepsis syndrome and septic shock remain the leading cause of mortality in intensive care units [22,23].

**Figure 1:**
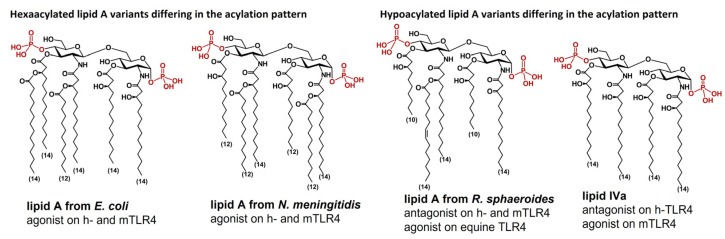
Chemical structure of the endotoxic part of LPS, lipid A.

TLR4 is a type I transmembrane protein comprising an extracellular leucine-rich repeat ectodomain that is physically associated with an accessory molecule, MD-2, which is responsible for LPS recognition and binding, a transmembrane domain, and a cytosolic toll/interleukin-1 receptor (TIR) domain that mediates the induction of downstream signaling [24,25]. The binding of a natural TLR4 ligand LPS promotes the dimerisation of two TLR4/MD-2/LPS complexes, resulting in the activation of intracellular adaptor proteins and the formation of an intracellular supramolecular organising centre (SMOC) that directs the induction of pro-inflammatory signaling cascades [26,27,28]. The TLR4/MD-2 complex is particularly sensitive to Gram-negative bacterial endotoxin (lipopolysaccharide, LPS) and can respond to picomolar doses of LPS. The innermost terminal part of LPS which is recognised by the TLR4/MD-2 complex is termed lipid A and consists of a β(1→6)-linked diglucosamine equipped with multiple lipid chains (either long-chain (*R*)-3-hydroxyacyl or (*R*)-3-acyloxyacyl residues attached to C3/C3′-hydroxyl groups and C2/C2′-amino groups) and usually two phosphate groups, one in the anomeric position, and one in position 4′ (Figure 1).

The spacious Leu- and Phe-rich hydrophobic binding pocket of MD-2 is large enough to accommodate four to five lipid tails of about C_12_–C_14_ length (as in the case of hexaacylated *E. coli* lipid A), while the sixth lipid chain is pushed out of the pocket and exposed on the exterior side of MD-2, where it is engaged in the dimerisation process with the second TLR4* complex [24]. The binding of LPS leads to structural rearrangements in MD-2, which enables the dimerisation of two receptor complexes and, thus, represents a key event in the ligand-induced TLR4 complex activation [24,25]. In agreement with crystallographic and biochemical studies, the binding of MD-2 ligands is largely mediated by hydrophobic interactions [29,30], while the salt bridges play a key role in the recognition and positioning of LPS/lipid A in the binding pocket of human (h)-MD-2 as well as in driving dimerisation [31,32].

Several LPS/lipid A variants, such as LPS from *R. sphaeroides*, the synthetic compound Eritoran, and a biosynthetic precursor of *E. coli* LPS, lipid IVa, have been shown to bind selectively to the human MD-2/TLR4 complex without inducing dimerisation [33,34,35]. The lipid A portion of the *R. sphaeroides* LPS contains five lipid chains, and the lipid VIa contains four lipid chains and two phosphate groups attached at positions 4′ and 1 of the β(1→6)-linked diglucosamine backbone (Figure 1). In the protein-bound state, all lipid chains of a TLR4 antagonist are fully interpolated into the deep hydrophobic pocket of MD-2 [33,34]. Thus, the binding of hypoacylated LPS/lipid A with the appropriate lipid chain length in the binding pocket of MD-2 can prevent interaction with the endotoxic LPS, and these lipid A variants are considered TLR4 antagonists. These compounds have been systematically investigated as potential antiseptic drug candidates but, unfortunately, none have been found to be effective in preventing or therapeutically inhibiting the destructive endotoxic effects caused by LPS-induced TLR4 activation [21,36].

Thus, the only molecular requirements for a TLR4 antagonist resulting from the LPS/TLR4 structure–activity relationships are the number and length of lipid chains attached at positions 2,2′ and 3,3′ of the glucosamine backbone, which must be fully intercalated into the deep binding pocket of MD-2 and the presence of at least one phosphate group. However, these structural prerequisites do not account for possible species-specific recognition.

One of the less understood phenomena in the interaction between TLR4 complex and its ligands is species specificity [37]. The molecular mechanisms underlying the structural determinants of the species-specific recognition of underacylated LPS/lipid A variants, such as tetraacylated lipid IVa (a biosynthetic precursor of *E. coli* lipid A), which antagonises the human but activates the mouse TLR4 complex [25,38], or pentaacylated LPS from *Rhodobacter sphaeroides*, which acts as an antagonist on human TLR4/MD-2 but as an agonist of the equine receptor complex [39], have been extensively studied [25]. Specific side chains on both MD-2 and TLR4 were found to be involved in the species-specific recognition of hypoacylated LPS variants. In particular, the electrostatic surface charges near the entrance of the hydrophobic binding pocket, which differ between human, mouse, and equine MD-2, were shown to influence species specificity. For example, Lys122 and Lys125 are thought to control the correct positioning of the lipid A molecule in the binding pocket (which was found to differ by 180° for agonist and antagonist ligands), such that the replacement of the positively charged K122/K125 on human MD-2 with either negatively charged or hydrophobic residues in mouse and equine MD-2 (E122/L125 for mouse MD-2 and R122/R125 for equine MD-2) was shown to confer responsiveness to lipid IVa in mice [40,41]. Similarly, mutations of hydrophobic side chains in MD-2 as well as specific mutations on TLR4 conferred differential levels of responsiveness to underacylated endotoxin [42].

Considering the inherent plasticity of the LPS-binding co-receptor MD-2, which can adopt different conformational states in a ligand structure-dependent manner [43], as well as its species-specificity in ligand recognition (e.g., human vs. mouse) [25], the development of strategies to inhibit MD-2/TLR4-mediated signaling by designing appropriate MD-2 antagonists is predestined to lack accuracy and predictability. The fact that agonist and antagonist lipid A, regardless of its acylation status, was found to be bound in two opposite orientations (+/− 180°) in the hydrophobic cavity of MD-2 also adds to the confusion in predicting the biological properties of lipid A-like TLR4 ligands.

Since the native TLR4 antagonists, which are typically the underacylated lipid A portions of bacterial LPS, are also intrinsically flexible molecules based on the βGlcN(1→6)GlcN backbone which can easily adjust its molecular shape upon protein binding, limiting the flexibility of TLR4/MD-2 specific lipid A-mimicking ligands by rigidifying their carbohydrate backbone may provide improvements.

## 2. Results and Discussion

**Figure 2:**
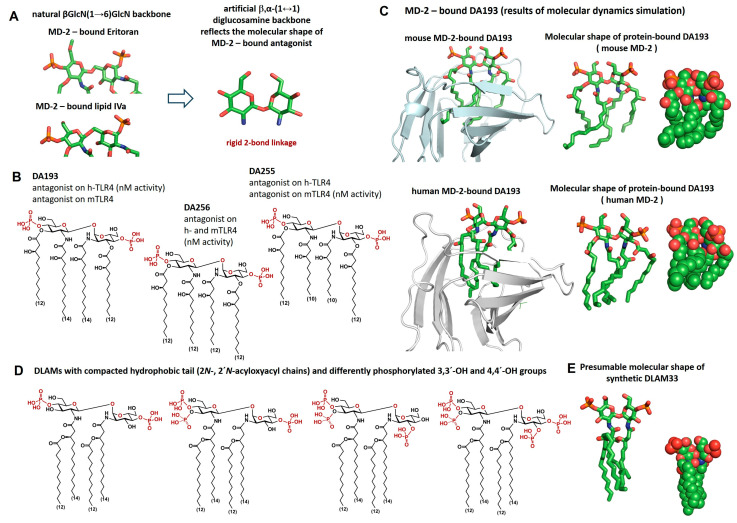
The rational for the synthesis of a novel series of disaccharide-based TLR4 antagonists: co-crystal structure-based design. (**A**) Structural basis for TLR4 antagonism of glycolipids based on the β,α-1,1-linked diglucosamine backbone. The co-planar orientation of two GlcN rings in the βGlcN(1↔1)αGlcN scaffold reflects the tertiary structure of the βGlcN(1→6)GlcN backbone of the protein-bound lipid A. (**B**) Chemical structure of TLR4 antagonists based on the non-reducing βGlcN(1↔1)αGlcN scaffold. (**C**) Mouse MD-2- and human MD-2-bound synthetic TLR4 antagonist **DA193** (according to molecular dynamics simulation study [45]) and its molecular shape (stick-and sphere models). Images were generated using PyMol. (**D**) Chemical structure of synthetic poly-phosphorylated glycolipids. (**E**) Proposed molecular shape of *2N,2′N*-acylated DLAM.

We have previously demonstrated that restricting the flexibility of the disaccharide backbone of lipid IVa byreplacing it with an inherently rigid β,α-1,1-linked non-reducing disaccharide scaffold, abolishes the inherent species specificity, turning conformationally constrained lipid A mimetics (disaccharide lipid A mimetics, DLAMs) into powerful antagonists on both h- and mTLR4/MD-2 (Figure 2A) [44]. We have also shown that lipid A mimetics based on the βGlcN(1↔1)αGlcN backbone can both inhibit LPS binding to MD-2 and displace LPS from the binding pocket of the co-receptor protein. Furthermore, both possible binding orientations of the molecule (+/− 180°) in the pocket of MD-2 were equally effective at inhibiting the pro-inflammatory responses to LPS [45].

However, the anti-endotoxic activity of antagonistic DLAMs was dependent on the length of the β-hydroxyacyl residues (C_10_–C_14_) attached at positions 2,2′ and 3,3′ of the β,α-1,1-linked diglucosamine backbone (Figure 2B). While DLAM DA193 (acylation pattern C_12_C_14_C_14_C_12_) showed excellent antagonist activity on hTLR4, it was slightly less efficient in the mouse system, whereas DLAM DA254 (acylation pattern C_12_C_10_C_10_C_12_) was the most effective TLR4 inhibitor in mice, although somewhat less potent on hTLR4. DLAM with an “intermediate” lipid chain length (4 × C_12_) was highlighted as an “all-rounder” for anti-endotoxic activity on both human and murine TLR4 [46]. An analysis of the 3-D molecular shape of the human and mouse MD-2-bound DA193 with two co-planar arranged GlcN rings revealed that the β-hydroxyacyl lipid chains form a voluminous hydrophobic cluster (Figure 2C), but the positioning of the ligands within the binding pocket is different for human and mouse MD-2. We hypothesised that narrowing the hydrophobic region by shifting the lipid chains in the center of the molecule (as shown in Figure 2D) would change the shape of the hydrophobic portion of the glycolipid and make it less bulky (as shown in Figure 2E), which could enhance its affinity to mMD-2 and, thus, reduce the species-specificity in ligand recognition. Such an acylation pattern also seems attractive because of the sufficient chemical stability of the acyl bond linking the secondary fatty acid to the β-hydroxy group on the *N*-acyl chain. This linkage should also be more resistant to potential degradation by acyloxyacyl hydrolases, which can cleave secondary acyl chains from LPS [47,48].

The phosphate groups attached to the 4′ and 1- positions of lipid A were confirmed to be essential for the biological activity of LPS, as the binding capacity of partially dephosphorylated variants (e.g., monophosphates) decreases many-fold compared to that of diphosphates [31,32]. Importantly, the inner core of LPS provides additional negative charges (Kdo residues, phosphorylated heptoses) for interaction with the receptor complex, which makes rough LPS strains and truncated *Re* -LPS variants at least 10-fold more active than lipid A. With the aim of enhancing the affinity to both human and mouse MD-2 and of probing the influence of additional phosphate groups on that activity, we performed the synthesis of lipid A-mimicking glycolipids with branched acyloxyacyl chains attached to the 2- and 2′-amino groups of the artificial βGlcN(1↔1)αGlcN backbone and multiple phosphate groups (Figure 2D).

The self-organisation of amphiphilic glycolipids in aqueous media is strongly dependent on their primary chemical structure, in particular on their hydrophilic–hydrophobic balance and the 3D-molecular shape of the amphiphile [49]. Since TLR4/MD-2 ligands must first be extracted from surfaces (membranes, liposomes, etc.) by the LPS-binding protein (LBP) and then sequentially transferred to the accessory molecule cluster of differentiation 14 (CD-14), which shuttles the glycolipid ligand to the binding pocket of MD-2 [50], the supramolecular structure of glycolipid aggregates in aqueous media plays a key role in the recognition process [51,52]. In order to maintain the hydrophilic–hydrophobic balance in βα-DLAMs and to retain the potential hydrogen bond-donating functional groups required for ligand–protein interactions, a replacement for 3-OH groups on the lipid chains (now substituted by secondary acyl chains) was necessary. Therefore, the C3-OH and C3′-OH groups on both glucosamines had to remain unsubstituted or be phosphorylated (Figure 2D). An additional phosphate group attached to C3-OH and/or C3′-OH could be important for an appropriate positioning and fitting orientation of the ligand in the binding pocket of MD-2, which is driven by the positively charged side chains at the pocket edge.

### 2.1. Chemical Synthesis

**Scheme 1:**
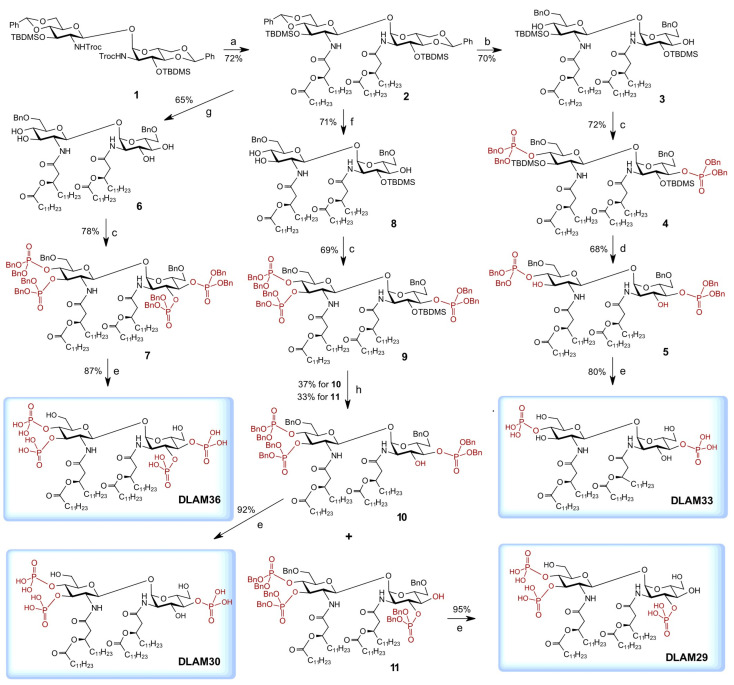
Synthesis of 2,2′-*N,N*-acyloxyacyl disaccharide lipid A mimetics *βα*-DLAMs. Reagents and conditions: (**a**) Zn, AcOH, then C_11_H_23_O(C_11_H_23_CO)CH_2_COOH [53], DIC; (**b**) TfOH (2.5 eq), Et_3_SiH (2.5 eq), DCM, −85 °C; (**c**) (BnO)_2_PN(*i*Pr)_2_, 1*H*-tetrazole, DCM-CH_3_CN, r.t., then *m*-CPBA, −78° C; (**d**) HF∙Py, DCM, 0 °C; (**e**) Pd-black, H_2_ toluene-MeOH, 1:1; (**f**) TfOH (2.5 eq), Et_3_SiH (2.5 eq), DCM, −65 °C; (**g**) TfOH (8 eq), Et_3_SiH (8 eq), DCM, −70 °C; (**h**) TBAF, THF, 0 °C.

An orthogonally protected β,α-1,1-linked diglucosamine **1** was used for the synthesis of variably phosphorylated, conformationally constrained glycolipids [44]. The 2,2′-*N*-Troc groups in **1** were reductively cleaved and the liberated amino groups were acylated with a branched (*R*)-3-(dodecanoyloxy)tetradecanoic acid to obtain a key intermediate—fully protected tetraacylated **2** (Scheme 1). The reductive opening of two benzylidene acetal protecting groups in **2** using trifluoromethanesulfonic acid (TfOH) and triethylsilane (Et_3_SiH) in dichloromethane (DCM) at −78 °C proved to be challenging and resulted in 3→4 migration and the partial hydrolysis of 3,3′-*O*-*tert*-butyldimethylsilyl (TBDMS) groups. The conditions were carefully optimised and the regioselective reductive opening was conducted using TfOH (2.5 equiv.) as a Lewis acid and Et_3_SiH (2.5 equiv.) as a reducing reagent in DCM at a strictly controlled temperature of −85 °C, yielding a 6,6′-*O*-benzylated compound **3** with two free OH groups at positions 4 and 4′. Negligent deviations from the established reaction conditions (e.g., raising the reaction temperature to −75 °C or increasing the amount of reducing reagent/Lewis acid) resulted in either partial hydrolysis or the migration of TBDMS groups. The 4- and 4′- hydroxyl groups were then phosphitylated by reaction with bis(benzyloxy)(diisopropylamino)phosphine in the presence of 1*H*-tetrazole followed by oxidation with *meta*-chloroperoxybenzoic acid (*m*CPBA) at −78° C to give the diphosphate **4**. A stepwise deprotection procedure consisting of the cleavage of 3,3′-*O*-TBDMS groups with pyridinium hydrofluoride at 0 °C to obtain **5** and the hydrogenolysis of benzyl protecting groups with H_2_ over Pd-black afforded the target tetraacylated 4,4′-diphosphate **DLAM33**.

To achieve the synthesis of 3,3′-4,4′- tetraphosphorylated **DLAM36**, we investigated the possibility of the reductive opening of benzylidene acetal in **2** with the simultaneous removal of 3,3′-*O*-TBDMS groups (Scheme 1). Using an excess of both the reductive reagent and Lewis acid (TfOH, 8 equiv.; Et_3_SiH, 8 equiv.) in DCM and raising the reaction temperature from −85 °C to −70 °C, the benzylidene acetal in **2** was regioselectively opened with the simultaneous cleavage of both TBDMS groups to afford 6,6′-benzylated tetraol **6**. Phosphitylation, using the phosphoramidite approach, afforded tetraphosphorylated **7**, which was readily deprotected by the hydrogenolysis of benzyl protecting groups on phosphorus to obtain the target **DLAM36**.

With the aim of obtaining tri-phosphorylated lipid A mimetics, a desymmetrisation of **1** or its tetraacylated derivative **6** was attempted by differentiating the positions 4,6 and 4′,6′ using several orthogonal protecting groups. However, the synthesis was low-yielding, as the differentiation of the hydroxyl groups of almost similar reactivity (C4-OH vs. C4′-OH and C6-OH vs. C6′-OH on the β-and α-GlcN moieties) using orthogonal protecting groups resulted in complex mixtures and required tedious chromatographic separations. This prompted further investigation into the possibility of a reductive opening of the 4,6-benzylidene acetal in **2** with simultaneous regioselective hydrolysis of one of the TBDMS groups. Accordingly, using a modified procedure, a regioselective reductive opening of benzylidene acetal with concomitant regioselective hydrolysis of the 3′-*O*-TBDMS group was achieved to afford the 6,6′-benzylated 3-*O*-TBDMS derivative **8** (Scheme 1). The phosphorylation of three hydroxyl groups in **8** using the phosphoramidite approach by first reacting with bis(benzyloxy)(diisopropylamino)phosphine in the presence of 1*H*-tetrazole as a weak acid catalyst followed by oxidation with *meta*-chloroperoxybenzoic acid (*m*CPBA) at −78 °C gave the triphosphate **9**. The positions of attachment of the TBDMS protecting groups and phosphate groups were confirmed by ^29^Si-^1^H HMBC- and ^31^P-^1^H HMBC-NMR experiments, respectively.

In general, fluoride-ion-mediated deprotection of the TBDMS group can be carried out under mild conditions at a slightly acidic pH (HF‧Py, pH 3–4), near neutral conditions (TREAT-HF, 3HF‧Et_3_N, pH 5–6) or under more hash conditions (TBAF), while the outcome of the reaction and the stability of other protective groups in the molecule additionally depend on the concentration of fluoride reagent in the reaction solution [54]. Thus, the 3-*O*-TBDMS group in **9** could be readily cleaved using a concentrated solution of HF‧Py (as for **4** → **5**) or TREAT to obtain **10**. In our previous work, we often had to deal with the unwanted migration of dibenzyl phosphates when deprotecting the adjacent TBDMS group with TBAF. Here, however, we were able to take advantage of the concomitant migration of the TBDMS protecting group to produce an additional positional isomer of the tri-phosphorylated disaccharide. The application of the carefully adjusted conditions (TBAF, THF, 0 °C) to compound **9** resulted in TBDMS deprotection with concomitant migration of the 4-*O*-phosphate group to position 3, providing us with a synthetic precursor with an altered phosphorylation pattern. Tri-phosphorylated regioisomers were readily isolated in pure form to give 3′,4′,4-phosphate **10** and 3′,4′,3-phosphate **11**. Global deprotection under hydrogenolytic conditions (H_2_/Pd-black) afforded tetraacylated triphosphates **DLAM30** and **DLAM29**, respectively.

Thus, four variably phosphorylated glycolipids were efficiently prepared from a single tetraacylated precursor with minimal manipulation of the protecting groups, exploiting the notorious (and usually undesirable) ability of the TBDMS and phosphate groups to migrate.

### 2.2. Immunobiology

The in vitro assays that we typically used to screen our synthetic TLR4 antagonists for anti-endotoxic activity were usually performed using a constant concentration of LPS (the one that induces the maximal response in the respective cell type, e.g., 10 ng/mL *E. coli* LPS for human mononuclear cells (MNC), 100 ng/mL *E. coli* LPS for the human leukemia monocytic (THP-1) cell line, etc.) and an increasing concentration of TLR4/MD-2 antagonist (10 ng/mL to 1000 ng/mL). A compound was considered antagonistic if LPS-induced cytokine release was fully suppressed by the application of 100 nM of antagonist. However, in order to more accurately reproduce the effect of a potential TLR4 antagonist in the in vivo setting, where the concentration of LPS in the tissue or bloodstream cannot be controlled, it may be useful to challenge the cell culture with increasing concentrations of LPS while keeping the concentration of TLR4 antagonist at a constant level.

**Figure 3:**
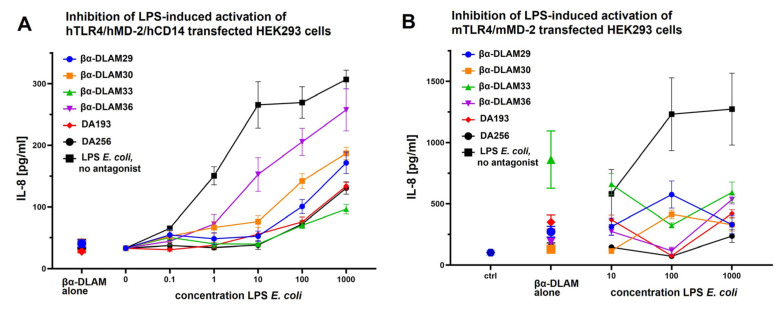
Dose-dependent inhibition of LPS-induced TLR4 signaling by βα-DLAMs. (**A**) Inhibition of *E. coli* O111:B4 LPS-induced IL-8 release in hTLR4/hMD-2/hCD14—transfected HEK293 cells comparted to TLR4 antagonists DA193 and DA256; (**B**) inhibition of *E. coli* O111:B4 LPS-induced IL-8 release in mTLR4/mMD-2—transfected HEK293 cells comparted to TLR4 antagonists DA193 and DA256. Data were combined from n = 3 independent experiments, error bars indicate standard error of the mean.

First, the ability of DLAMs to inhibit the induction of pro-inflammatory signaling was assessed in h- and mTLR4-transfected human embryonic kidney 293 cells (HEK293) (Figure 3). The cells were pre-treated with synthetic antagonists (10 µg/mL) for 60 min and subsequently challenged with increasing concentrations of *E. coli* LPS (0.1–1000 ng/mL). The TLR4-dependent activation of the cells was analysed by assessing the induction of interleukin-8 (IL-8) and compared with the responses obtained after inhibition with the previously developed TLR4 antagonists DA193 and DA256 [44] (Figure 2).

Inhibition of IL-8 release in hTLR4/MD-2/CD14 transfected HEK293 cells (co-transfection with CD14 significantly increases the sensitivity of the TLR4/MD-2 complex to LPS by transferring the monomeric endotoxin from the LPS-binding protein to MD-2 [50,55]) was strongly dependent on the chemical structure of the antagonist and the concentration of LPS in the culture (Figure 3A). When the cells were challenged with low doses of LPS (below 10 ng/mL), all compounds except for tetra-phosphorylated **DLAM36** efficiently suppressed IL-8 release. At higher LPS concentrations (10–1000 ng/mL), tetra-phosphorylated **DLAM36** showed the lowest antagonistic activity, whereas **DLAM33** showed the strongest inhibitory potential, even overpowering DA193’s antagonistic capacity.

Tri-phosphorylated **DLAM29** and **DLAM30** also deserve attention as potential TLR4 inhibitors, as these two compounds were able to suppress IL-8 release to half of the initial level even at extremely high LPS concentrations of 1000 ng/mL. None of the compounds had an activating effect in the hTLR4-transfected HEK293 cells.

The situation was quite different in the mTLR4/mMD-2 transfected HEK293 cell line, where the most potent hTLR4 antagonist **DLAM33** showed significant agonist potential at a concentration of 1 µg/mL (Figure 3B). However, in complex with increasing concentrations of LPS, the release of IL-8 was significantly lower compared to the initial levels induced by **DLAM33** alone. Other compounds were able to inhibit the production of IL-8 to at least a third of the original cytokine level, with the highest level of suppression being achieved at a specific LPS concentration of 100 ng/mL. Such a pronounced concentration-dependent behavior could be related to different aggregation states at different DLAM/LPS ratios. While at lower doses of LPS (1–100 ng/mL) the concentration of free LPS should be negligible as it is consumed by binding to TLR4/MD-2 (confirmed by the steadily increasing levels of IL-8), receptor saturation appears to already be reached at an LPS concentration of 100 ng/mL (the level of IL-8 release reaches a plateau). Thus, at higher doses of LPS (above 100 ng/mL), the concentration of free LPS in the cell culture increases, which may lead to the formation of mixed aggregation forms of DLAMs with LPS and affect the delivery of both LPS and DLAMs to the TLR4 complex, especially in the absence of membrane (m)CD14.

**Figure 4:**
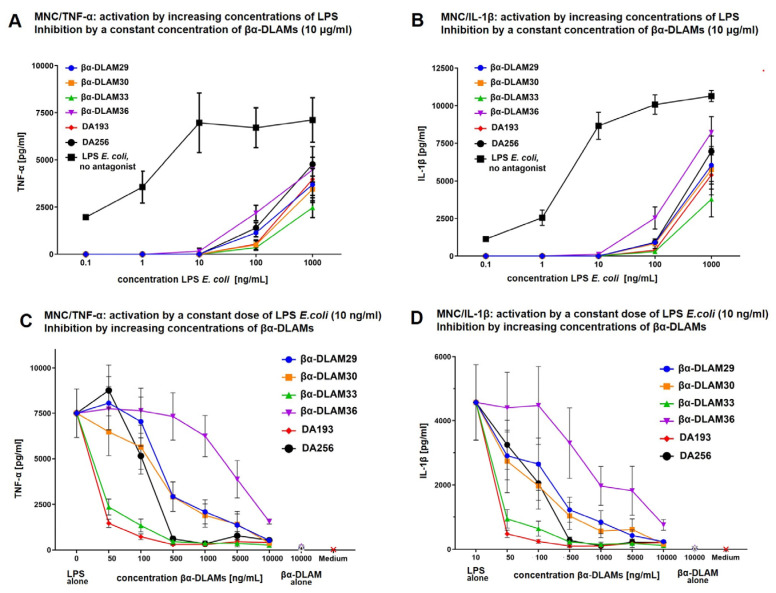
Dose-dependent inhibition of LPS-induced cytokine release in mononuclear cells (MNC) by βα-DLAMs. (**A**) Suppression of *E. coli* O111:B4 LPS-induced TNF-α release by βα-DLAMs (10 µg/mL). MNC were pre-incubated with 10 µg/mL βα-DLAMs and challenged with increasing concentrations of *E. coli* O111:B4 LPS (1–1000 ng/mL); (**B**) inhibition of *E. coli* O111:B4 LPS-induced IL-1β release by βα-DLAMs (10 µg/mL). MNC were pre-incubated with 10 µg/mL βα-DLAMs and challenged with increasing concentrations of *E. coli* O111:B4 LPS (1–1000 ng/mL); (**C**) suppression of TNF-α release by βα-DLAMs. MNC were pre-incubated with increasing concentrations of DLAMs and challenged with *E. coli* O111:B4 LPS (10 ng/mL); (**D**) inhibition of IL-1β production by βα-DLAMs. MNC were pre-incubated with increasing concentrations of DLAMs and challenged with *E. coli* O111:B4 LPS (10 ng/mL). Data shown are combined from n = 3 independent experiments, error bars indicate standard error of the mean.

To test the effect of DLAM antagonists on the LPS-induced pro-inflammatory signaling in primary immune cells, human mononuclear cells (MNC) were pre-incubated with DLAMs for 60 min. and subsequently treated with increasing concentrations of *E. coli LPS* (0.1–1000 ng/mL) (Figure 4A,B). In this experimental setting, all antagonists (applied at a concentration 10 µg/mL) demonstrated significant inhibitory potential at LPS concentrations up to 100 ng/mL. Considering that receptor saturation must have been attained at 10 ng/mL LPS (the concentration at which the release of tumor necrosis factor-α (TNF-α) reaches a plateau), the process of LPS recognition at concentrations above 100 ng/mL may involve other or additional molecular mechanisms which are closely related to the aggregation behavior of LPS and interaction with other proteins of the LPS transfer cascade [50]. However, even at an LPS concentration of 1000 ng/mL, **DLAM33** was extraordinarily effective in suppressing the release of both TNF-α and IL-1β to one third of the original levels.

In the next experiment, MNC were preincubated with increasing concentrations of DLAMs (1–10,000 ng/mL), while the concentration of LPS used to challenge the cells was set at 10 ng/mL (the average concentration sufficient to occupy all available TLR4/MD-2 complexes) (Figure 4C,D). Under this experimental setup, the diphosphate **DLAM33** substantially outperformed its multi-phosphorylated counterparts and was as efficient as DA193 in suppressing the release of both TNF-α and IL-1β. The tri-phosphorylated regio-isomers **DLAM29** and **DLAM30** showed similar inhibition profiles but were somewhat less potent than the previously developed DA193 and DA256. The inhibitory activity of the tetraphosphate **DLAM36** was very low.

**Figure 5:**
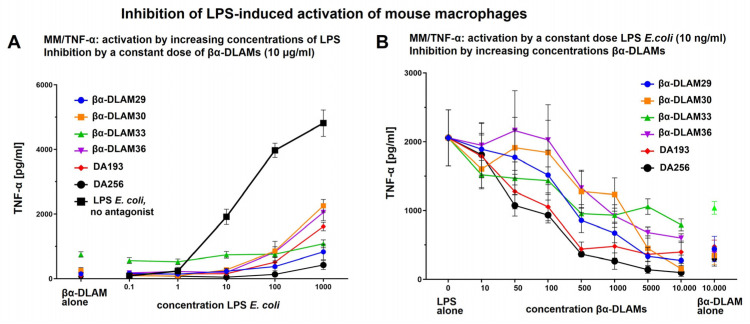
Dose-dependent inhibition of LPS-induced expression of TNF-α in mouse macrophages with antagonist DLAMs. (**A**) Inhibition of the release of TNF-α induced by increasing concentrations of *E. coli* O111:B4 LPS with 10 µg/mL DLAMs; (**B**) dose-dependent inhibitory effect of antagonist DLAMs on the expression of TNF-α induced by 10 ng/mL *E. coli* O111:B4 LPS. Data shown are combined from n = 2 independent experiments, error bars indicate standard error of the mean.

Considering the unusual behavior of **DLAM33** in mTLR4/mMD-2-transfected HEK293 cells, which was attributed to a very high concentration of TLR4 ligand used, we tested the inhibitory ability of synthetic compounds in mouse macrophages using different experimental settings (Figure 5). After the application of high concentrations of DLAMs (10 µg/mL), all compounds suppressed TNF-α release in an LPS concentration-dependent manner, with **DLAM29** being the most effective (compared to DA193 and DA256). **DLAM33**, on the other hand, could be qualified as a “partial agonist”, since it supported a low degree of activation and simultaneously suppressed the cytokine release induced by LPS. In an opposite setting (when cells were challenged with 10 ng/mL LPS and TNF-α production was inhibited by increasing concentrations of antagonists), about a 50% reduction in TNF-α release was achieved at a concentration of 500 ng/mL for **DLAM33** and **DLAM29**, whereas further increases in concentration did not result in any improvement for **DLAM33**, supporting its “partial agonist” properties.

**Figure 6:**
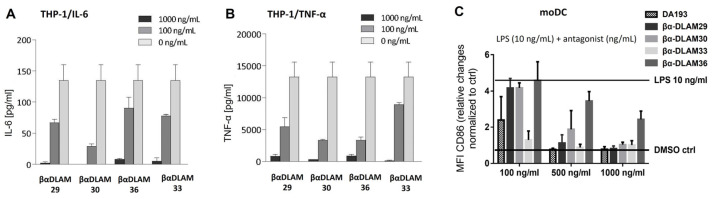
Inhibition of the *E. coli* O111:B4 LPS-induced signaling in THP-1 cells and DC by antagonist DLAMs. (**A**) Inhibition of release of IL-6 in THP-1 cell line; (**B**) inhibition of induction of TNF-α in THP-1 cells; data are the mean of n = 3 samples and are representative of n = 3 independent experiments. Error bars indicate standard deviation. (**C**) Inhibitory effects of TLR4 antagonists on LPS-induced DC maturation. Bars represent median fluorescent intensities (MFIs) of DC activation/maturation marker CD86 by gated CD1a^+^CD11b^+^ moDCs normalized to untreated controls (n = 3). Lower horizontal line represents values of DMSO control; upper horizontal line represents values of 10 ng/mL LPS without TLR antagonists. Data are the mean of n = 3 independent experiments. Error bars indicate standard deviation of the mean.

To confirm the biological activity on hTLR4, we further investigated the antagonistic potential of differently phosphorylated DLAMs in the LPS-challenged human monocytic cell line THP-1 and dendritic cells (DC) (Figure 6). The TPA-(12-O-tetradecanoylphorbol-13-acetate)-primed THP-1 macrophage cell line was pre-incubated with DLAMs and subsequently stimulated with *E. coli* LPS. The THP-1 cell line was less sensitive to the differences in the phosphorylation pattern, so that all four DLAMs could completely suppress the release of IL-6 and TNF-α at a concentration of 1000 ng/mL, whereas about 50% inhibition was achieved at a concentration of 100 ng/mL.

DCs are highly specialised in sensing microbial signals and are known as the sentinels of the immune system. We chose ex vivo-generated human monocyte-derived DCs (moDCs) for the functional evaluation of TLR4 inhibitors because these cells are the most extensively studied model DCs for in vitro studies. They phenotypically resemble both inflammation-associated DCs, which are known to appear rapidly at sites of inflammation, and tissue-resident DCs, which populate peripheral tissues in the steady state. moDCs are known to express high levels of TLR4 and to respond rapidly to LPS challenge by upregulating the T cell co-stimulatory molecules such as cluster of differentiation 80 (CD80) and cluster of differentiation 86 (CD86), a phenotypic hallmark of DC activation and maturation. Therefore, the ability of DLAMs to inhibit LPS-induced inflammatory pathways by down-regulating the specific surface marker CD86 in dendritic cells was investigated. Monocyte-derived immature DCs were treated with LPS with or without the addition of four variably phosphorylated DLAMs. LPS-treated DCs acquired a characteristic morphological phenotype and displayed specific markers of mature DCs when analysed by flow cytometry. A short pre-incubation with di- and tri-phosphorylated DLAMs (1000 ng/mL) completely blocked the LPS-induced up-regulation of the co-stimulatory molecule CD86. When the concentration was reduced to the nanomolar range, **DLAM33** was confirmed as the most potent inhibitor (suppression of CD86 to background levels), whereas the tri-phosphate **DLAM29** showed full potency at a concentration of 500 ng/mL. The reduced activity of the tetra-phosphate **DLAM36** in DCs is consistent with data obtained for other cell types.

**Figure 7:**
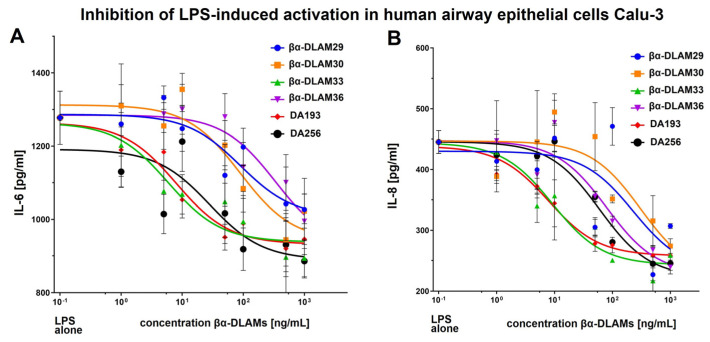
Inhibition of *E. coli* O111:B4 LPS-induced expression of IL-6 and IL-8 in human epithelial cell line Calu-3 compared to TLR4 antagonists DA193 and DA256. (**A**) Suppression of IL-6 production by antagonist DLAMs; (**B**) inhibition of LPS-induced production of IL-8 by antagonist DLAMs. Data shown are combined from n = 3 (IL-6)/n = 2 (IL-8) independent experiments, error bars indicate standard error of the mean.

Since Gram-negative airway inflammation is strongly associated with the sensitisation of the airway epithelium to LPS, we investigated the ability of DLAMs to inhibit LPS-induced cytokine release in the human airway epithelial cell line Calu-3 (Figure 7). **DLAM33** was able to suppress IL-6 and IL-8 production as efficiently as DA193 (nanomolar activity), whereas hyperphosphorylation rendered the molecules less effective in airway epithelial cells. Interestingly, the differences between tetra- and tri-phosphates were less pronounced compared to other cell types, and the tetra-phosphate **DLAM36** even showed similar activity to DA256 in inhibiting IL-8 release in Calu-3 (Figure 7B).

## 3. Materials and Methods

### 3.1. Chemical Synthesis

#### General Synthetic Methods

Reagents and solvents were purchased from commercial suppliers and used without further purification unless otherwise stated. Toluene was dried by distillation first over phosphorus pentoxide then over calcium hydride and was then stored over activated 4 Å molecular sieves (MS). Solvents were dried by storage over activated MS for at least 24 h prior to use (dichloromethane 4 Å, acetonitrile, and DMF 3 Å). Residual moisture was determined by colorimetric titration on a Mitsubishi CA21 Karl Fischer apparatus and did not exceed 20 ppm. Reactions were monitored by TLC performed on silica gel 60 F254 HPTLC precoated glass plates with a 25 mm concentration zone (Merck, Darmstadt, Germany). Spots were visualized by dipping into a sulfuric acid—*p*-anisaldehyde solution and subsequent charring at 250 °C. Solvents were removed under reduced pressure at ≤40 °C. Column chromatography was performed using silica gel 60 (0.040–0.063 mm). Size exclusion chromatography was performed using Bio-Beads S-X1 support (BioRad, Darmstadt, Germany). NMR spectra were recorded on a Bruker Avance III 600 spectrometer (^1^H at 600.22 MHz; ^13^C at 150.93 MHz; ^31^P at 242.97 MHz) using standard Bruker NMR software. Chemical shifts are reported in ppm, where ^1^H NMR spectra recorded from samples in CDCl_3_ were referenced to internal TMS and ^13^C spectra were referenced to the corresponding solvent signal (77.16 ppm for CDCl_3_). NMR spectra recorded from samples in other solvents were referenced to residual solvent signals (for CD_3_OD 3.31 and 49.00 ppm; for CD_2_Cl_2_ 5.32 and 53.84 ppm; for DMSO-d_6_ 2.50 and 39.52 ppm; for ^1^H- and ^13^C-NMR, respectively). NMR spectra recorded in CDCl_3_-MeOD (4:1, *v*/*v*) were referenced to residual solvent signals of CDCl_3_ (7.26 ppm and 77.16 ppm; ^1^H- and ^13^C-NMR, respectively). NMR spectra recorded in CDCl_3_:MeOD (1:1 to 4:1, *v*/*v*) were referenced to residual solvent signals of MeOD (3.31 and 49.00 ppm, ^1^H and ^13^C NMR, respectively). ^31^P-NMR spectra were referenced according to IUPAC recommendations from a referenced ^1^H-NMR spectrum. The NMR signals of the β-configured GlcN ring are indicated by primes. The NMR-spectra of all synthetic compounds can be found in the Supporting Information file. High-resolution mass spectrometry (HRMS) was carried out on acetonitrile or acetonitrile-dichloromethane solutions via LC-TOF MS (Agilent 1200SL HPLC and Agilent 6210 ESI-TOF, Agilent Technologies, Santa Clara, CA, USA). Datasets were analysed using Agilent Mass Hunter Software. MALDI-TOF MS was performed in negative-ion mode using a Bruker Autoflex Speed instrument with 6-aza-2-thiothymine (ATT) as matrix and ammonium sulfate as additive. Optical rotation was measured on a PerkinElmer 243B polarimeter (PerkinElmer equipped with a Haake water circulation bath and a Haake D1 immersion circulator for temperature control or an Anton Paar MCP 100 polarimeter (Anton Paar, Graz, Austria) featuring integrated Peltier temperature control. All α20D values are reported in units of deg*dm^−1^*cm^3^*g^−1^, the corresponding concentrations are reported in g/100 mL.

**4,6-*O*-Benzylidene-3-*O*-*tert-*butyldimethylsilyl-2-deoxy-2-[(*R*)-3-(dodecanoyloxy)-tetradecanoylamino]-β-d-glucopyranosyl-(1↔1)-4,6-*O*-benzylidene-3-*O*-*tert*-butyldimethylsilyl-2-deoxy-2-[(*R*)-3-(dodecanoyloxy)tetradecanoylamino]-α-d-glucopyranoside (2).** To a cooled (0 °C) stirred solution of **1** (156 mg, 140 µmol) in DCM (2 mL), acetic acid (3 mL) and Zn powder (330 mg, 5 mmol) were added and the mixture was stirred for 20 h at 0 °C then diluted with DCM (10 mL), the solids were removed by filtration over a pad of Celite, then the filtrate was diluted with toluene (10 mL) and concentrated. The residue was repeatedly co-evaporated with toluene (3 × 10 mL), redissolved in chloroform (50 mL), and washed with satd. aq. NaHCO_3_ (2 × 20 mL), water (20 mL), and brine (20 mL). The organic layer was dried over Na_2_SO_4_, filtered over cotton, and concentrated. The residue was taken up in dry DCM (5 mL) and solutions of (*R*)-3-(dodecanoyloxy)tetradecanoic acid C_11_H_23_O(C_11_H_23_CO)CH_2_COOH [53] (149 mg, 350 µmol) in DCM (4 mL) and DIC (44 mg, 55 µL, 350 µmol) were added under stirring in the atmosphere of Ar. The reaction mixture was stirred for 20 h, then diluted with EtOAc (50 mL) and washed with satd. aq. NaHCO_3_ (2 × 30 mL), water (30 mL), and brine (30 mL). The organic layer was dried over Na_2_SO_4_, filtered over cotton, and concentrated. The residue was purified by column chromatography on silica gel (toluene-EtOAc, 6:1) to give **2** (158 mg, 72%). R_f_ = 0.5 (toluene-EtOAc, 4:1); α25D = 9.0 (*c* = 1, CHCl_3_). ^1^H NMR (600 MHz, CDCl_3_): δ 7.49–7.45 (m, 4H, Ph), 7.37–7.34 (m, 6H, Ph), 6.71 (d, 1H, *J*_2-NH_ = 7.9 Hz, NH′), 5.97 (d, 1H, NH), 5.48, 5.47 (2s, 2H, C*H*Ph), 5.11 (m, 1H, βC*H*^Myr^), 5.03 (d, 1H, *J*_1′-2′_ = 8.7 Hz, H-1′), 5.02 (d, 1H, *J*_1-2_ = 1.5 Hz, H-1), 5.0 (m, 1H, βC*H*^Myr^), 4.26 (dd, 1H, *J*_5′-6′b_ = 4.8 Hz, H-6′b), 4.21 (ddd, 1H, *J*_2-3_ = 3.7, *J*_2-NH_= 9.6 Hz, H-2), 4.19 (t, 1H, *J*_2′-3′_ = *J*_3′-4′_ = 8.7 Hz, H-3′), 4.15 (dd, 1H, *J*_6a-6b_ = 10.4 Hz, H-6b), 4.05 (ddd, 1H, *J*_4-5_ = *J*_5-6a_ = 9.9 Hz, *J*_5-6b_ = 5.0 Hz, H-5), 3.86 (t, 1H, H-3), 3.75 (t, 1H, *J*_6′b-6′a_ = *J*_5′-6′a_= 10.0 Hz, H-6′a), 3.69 (t, 1H, H-6a), 3.52 (dd, 1H, *J*_3-4_ = 9.2 Hz, H-4), 3.51–3.47 (m, 2H, H-5′, H-2′), 3.45 (dd, 1H, *J*_4′-5′_ = 8.9 Hz, H-4′), 2.60–2.25 (m, 8H, 2×αC*H*_2_^Myr^_,_ 2×αC*H*_2_^Lau^), 1.68–1.57 (m, 8H, 2×βC*H*_2_^Lau^_,_ 2×γC*H*_2_^Myr^), 1.36–1.20 (m, 68H, C*H*_2_^Myr,Lau^), 0.89–0.87 (m, 12H, C*H*_3_^Myr,Lau^), 0.84 and 0.80 (2s, 18H, *t*BuSi), 0.02, 0.00, −0.04, and −0.05 (4s, 12H, SiMe). ^13^C NMR (150.9 MHz, CDCl_3_): δ 174.60, 173.81, 170.61, 169.75 (4C, 4×*C*=O), 137.50, 137.44 (2C, *C*_q_-Ph), 129.33, 129.26, 128.38, 128.35, 126.62 (10C, Ph), 102.21, 102.17 (2C, 2Ph*C*H), 100.18 (1C, C-1′), 99.15 (1C, C-1), 82.51 (1C, C-4), 82.24 (1C, C-4′), 72.49 (1C, *C*H^Myr^), 71.90 (1C, C-3′), 71.32 (1C, *C*H^Myr^), 71.07 (1C, C-3), 68.98 (2C, C-6, C-6′), 66.26 (1C, C-5′), 63.61 (1C, C-5), 59.68 (1C, C-2′), 54.41 (1C, C-2), 41.79 (4C, 2×α*C*^Myr^, 2×α*C*^Lau^), 35.06, 35.02, 34.97, 34.86, 32.18, 29.95, 29.90, 29,87, 29.81, 29.79, 29.74, 29.67, 29.62, 29.52, 29.50 (*C*H_2_^Myr,Lau^)_,_ 26.05, 25.97 (6C, *t*BuSi), 25.73, 25.64, 25.37, 25.27, 22.94, 22.51 (*C*H_2_^Myr,Lau^), 14.35 (4C, *C*H_3_^Myr,Lau^), −3.71, −3.77, −4.52, −4.57 (4C, SiMe). HRMS (ESI-TOF) *m*/*z*: found 1562.3742, calc. for [M + H]^+^ C_90_H_156_N_2_O_15_Si_2_ + H^+^: *m*/*z* = 1562.3744.

**6-*O*-Benzyl-3-*O*-*tert*-butyldimethylsilyl-2-deoxy-2-[(*R*)-3-(dodecanoyloxy)tetradecanoylamino]-β-d-glucopyranosyl-(1↔1)-6-*O*-benzyl-3-*O*-*tert*-butyldimethylsilyl-2-deoxy-2-[(*R*)-3-(dodecanoyloxy)tetradecanoylamino]-α-d-glucopyranoside (3).** To a stirred solution of **2** (97 mg, 62 µmol) in dry DCM (15 mL), molecular sieves 4 Å (100 mg) were added and the suspension was stirred for 2 h. at r.t. in an atmosphere of Ar. The mixture was cooled to −85 °C (hexane/liquid N_2_) and a solution of triethylsilane (25 µL, 150 µmol, 2.5 eq) in dry DCM (5% solution) was added under stirring. Next, a solution of trifluoromethanesulfonic acid (13 µL, 150 µmol, 2.5 eq) in dry DCM (5% solution) was added, and the stirring was continued for 4 h at −85 °C under atmosphere of Ar. The reaction mixture was quenched by addition of a solution of Et_3_N (30 µL, 220 µmol, 3.5 eq) in DCM (5% solution). The mixture was stirred for 15 min at −85 °C, then brought up to r.t. and diluted with DCM (50 mL). The solids were removed by filtration over a pad of Celite, the filtrate was washed with satd. aq. NaHCO_3_ (30 mL), water (30 mL), and brine (30 mL), dried over Na_2_SO_4_, filtered over cotton, and concentrated. The residue was purified by column chromatography on silica gel (toluene-EtOAc, 10:1 → 4:1) which gave **3** (68 mg, 70%). R_f_ = 0.3 (toluene-EtOAc, 3:1); α25D = 15 (*c* = 1, CHCl_3_). ^1^H NMR (600 MHz, CDCl_3_): δ 7.36–7.28 (m, 10H, Ph), 6.99 (d, 1H, NH′), 5.73 (d, 1H, NH), 5.27 (d, 1H, *J*_1′-2′_ = 9.0 Hz, H-1′), 5.21 (m, 1H, βC*H*^Myr^), 5.16 (d, 1H, *J*_1-2_ = 3.5 Hz, H-1), 5.07 (m, 1H, βC*H*^Myr^), 4.57–4.50 (m, 4H, C*H*_2_-Bn), 4.35 (t, 1H, *J*_3-4_ = 9.0 Hz, H-3′), 4.11 (ddd, 1H, *J*_4-5_ = 10.1 Hz, *J*_5-6b_ =2.4 Hz, *J*_5-6a_ = 7.7 Hz, H-5), 4.08 (dt, 1H, *J*_2-NH_ = *J*_2-3_ = 9.9 Hz, H-2), 3.85 (dd, 1H, H-6b), 3.71 (dd, 1H, *J*_6a′-6b′_ = 5.6 Hz, H-6a′), 3.66 (t, 1H, H-6b′), 3.65 (dd, 1H, *J*_3-4_ = 4.3 Hz, H-3), 3.55 (dt, 1H, *J*_5′-6a′_ = *J*_4′-5′_ = 9.9 Hz, *J*_5′-6b′_ = 5.1 Hz, H-5′), 3.47 (dd, 1H, *J*_6a-6b_ = 9.8 Hz, H-6a), 3.38 (t, 1H, H-4′), 3.27 (ddd, 1H, H-4), 3.16 (dt, 1H, *J*_2′-3′_ = 9.0 Hz, *J*_2′-NH′_ = 7.2 Hz, H-2′), 2.56–2.20 (m, 8H, 2×αC*H*_2_^Myr^_,_ 2×αC*H*_2_^Lau^), 1.64–1.52 (m, 8H, 2×βCH_2_^Lau^, 2×γCH_2_^Myr^), 1.30–1.21 (m, 68 H, C*H*_2_^Myr,Lau^), 0.91–0.83 (m, 30H, C*H*_3_^Myr,Lau^, *t*BuSi), 0.10, 0.09, 0.07, 0.05 (4s, 12H, SiMe). ^13^C NMR (150.9 MHz, CDCl_3_): δ 173.58, 173.29, 170.32, 169.55 (4C, 4×*C*=O), 137.79, 137.07 (2C, *C*_q_-Ph), 128.62, 128.49, 128.10, 128.01, 127.81, 127.64 (10C, Ph), 96.50 (1C, C-1′), 93.50 (1C, C-1), 73.95 (1C, C-4′), 73.72 (1C, C-3), 73.7, 73.61 (2C, *C*H_2_-Bn), 73.59 (1C, C-5), 73.25 (1C, C-3′), 72.71 (1C, C-4), 71.40 (1C, *C*H^Myr^), 71.28 (1C, C-5), 70.89 (1C, *C*H^Myr^), 70.80 (1C, C-6′), 70.60 (1C, C-6), 58.90 (1C, C-2′), 52.95 (1C, C-2), 41.78, 41.08 (4C, 2×α*C*^Myr^, 2×α*C*^Lau^), 34.95, 34.65, 34.61, 34.30, 31.92, 29.71, 29.67, 29-65, 29.64, 29.58, 29.56, 29.54, 29.49, 29.47, 29.36, 29.23, 29.21 (*C*H_2_^Myr,Lau^)_,_ 25.92, 25.78 (6C, *t*BuSi), 25.34, 25.22, 25.09, 25.07, 22.68 (*C*H_2_^Myr,Lau^)_,_ 14.10 (4C, *C*H_3_^Myr,Lau^), −4.02, −4.17, −4.32, −4.57 (4C, SiMe). HRMS (ESI-TOF) *m*/*z*: found 1566.1460, calc. for [M + H]^+^ C_90_H_160_N_2_O_15_Si_2_ + H^+^: *m*/*z* = 1566.1432.

**6-*O*-Benzyl-4-*O*-[bis(benzyloxy)phosphoryl]-3-*O*-*tert*-butyldimethylsilyl-2-deoxy-2-[(*R*)-3-(dodecanoyloxy)tetradecanoylamino]-β-d-glucopyranosyl-(1↔1)-6-*O*-benzyl-4-*O*-[bis(benzyloxy)phosphoryl]-3-*O*-*tert*-butyldimethylsilyl-2-deoxy-2-[(*R*)-3-(dodecanoyloxy)tetradecanoylamino]-α-d-glucopyranoside (4)**. To a stirred solution of 3 (40 mg, 26 µmol) in dry DCM (4 mL), bisbenzyloxy(diisopropylamino)phosphine (27 mg, 26 µL, 77 µmol) and a solution of 1*H*-tetrazol (16 mg, 155 µmol, 0.45 M in CH_3_CN) were added successively and the stirring was continued for 1 h at r.t. in an atmosphere of Ar. The reaction mixture was cooled to −78 °C, a solution of *m*-chloroperoxybenzoic acid (*m*CPBA) (33 mg, 116 µmol) in DCM (0.6 mL) was added, and the stirring was continued for 2 h. The reaction was quenched by addition of a solution of Et_3_N (50 µL) in DCM (0.5 mL) and the mixture was stirred for 10 min at −78 °C, then allowed to warm up to r.t. The mixture was diluted with DCM (50 mL) and washed with satd. aq. NaHCO_3_ (2 × 20 mL), water (20 mL), and brine (20 mL). The organic phase was dried over Na_2_SO_4_, filtered over cotton, and concentrated. The residue was purified by column chromatography on silica gel (toluene – EtOAc, 6:1 → 4:1) to give 4 (39 mg, 72%). R_f_ = 0.6 (toluene – EtOAc, 3:1); α20D = 18 (*c* = 1, CHCl_3_). ^1^H-NMR (600 MHz, CDCl_3_): δ 7.32–7.19 (m, 30H, Ph), 6.92 (d, 1H, *J*_NH′-2′_ = 7.8 Hz, NH′), 6.04 (d, 1H, *J*_NH-2_ = 9.6 Hz, NH), 5.21–5.14 (m, 2H, 2×βC*H*^Myr^), 4.98 (d, 1H, *J*_1-2_ = 3.1 Hz, H-1), 4.97 (d, 1H, *J*_1′-2′_ = 8.3 Hz, H-1′), 4.95–4.81 (m, 8H, 2×OP(O)(OC*H*_2_Ph)_2_), 4.52–4.35 (m, 4H, 2×C*H*_2_-Ph), 4.33 (m, 1H, H-4′), 4.32 (m, 1H, H-3′), 4.23 (ddd, 1H, *J*_3-4_ = *J*_4-5_ = 9.5 Hz, *J*_4-P_ = 9.0 Hz, H-4), 4.19 (ddd, 1H, *J*_1-2_ = 3.1 Hz, *J*_2-3_ = 6.7 Hz, *J*_2-NH_ = 9.6 Hz, H-2), 4.12 (ddd, 1H, *J*_5-6a_ = 5.5 Hz, *J*_5-6b_ = 2.0 Hz, H-5), 3.90 (dd, 1H, H-3), 3.88 (ddd, 1H, *J*_5′-6′a_ = 5.7 Hz, *J*_5′-6′b_ = 2.0 Hz, *J*_5′-4′_= 9.1 Hz, H-5′), 3.80 (dd, 1H, H-6′a), 3.78 (dd, 1H, H-6a), 3.70 (dd, 1H, H-6b), 3.69 (dd, 1H, H-6′b), 3.58 (m, 1H, H-2′), 2.63–2.18 (m, 8H, 2×αC*H*_2_^Myr^, 2×αC*H*_2_^Lau^), 1.61–1.53 (m, 8H, 2×βC*H*_2_^Lau^, 2×γC*H*_2_^Myr^), 1.30–1.22 (m, 68 H, C*H*_2_^Myr,Lau^), 0.89–0.86 (m, 12H, C*H*_3_^Myr,Lau^), 0.84 and 0.82 (2s, 18H, *t*BuSi), 0.09, 0.07, 0.06 and 0.03 (4s, 12H, SiMe). ^13^C-NMR (150.9 MHz, CDCl_3_): δ 173.17, 172.94, 170.21, 169.79 (4C, 4×*C*=O), 138.26, 138.09, 135.88, 135.84, 135.48, 135.42 (6C, *C*_q_-Ph), 128.68, 128.61, 128.59, 128.47, 128.41, 128.37, 128.28, 128.12, 128.10, 127.91, 127.49, 127.42 (30C, Ph), 98.68 (1C, C-1′), 96.77 (1C, C-1), 76.80 (C-3), 76.18 (C-4) 74.82 (1C, C-4′), 72.98, 72.89 (2C, 2×*C*H_2_-Ph), 71.56 (1C, C-3′), 71.29 (1C, C-5). 71.26 (1C, C-5′), 71.01 (1C, *C*H^Myr^), 70.60 (1C, *C*H^Myr^), 69.88, 69.85, 69.78, 69.75, 69.66, 69.62, 69.20, 69.16 (8C, 2×*C*H_2_Ph, 2×OP(O)(O*C*H*_2_*Ph)_2_, C-6, C-6′), 56.53 (1C, C-2), 53.15 (1C, C-2′), 41.49, 40.65 (4C, 2×α*C*^Myr^, 2×α*C*^Lau^), 34.58, 34.52, 34.47, 34.42, 31.93, 29.72, 29.68, 29.65, 29.62, 29.57, 29.55, 29.50, 29.37, 29.28, 29.24 (*C*H_2_^Myr,Lau^), 25.91, 25.88 (6C, *t*BuSi), 25.39, 25.33, 25.06, 25.05, 22.68 (*C*H_2_^Myr,Lau^)_,_ 14.09 (4C, *C*H_3_^Myr,Lau^), −3.07, −4.02, −4.50, and −4.62 (4C, SiMe). ^31^P NMR (243 MHz, CDCl_3_): δ −1.70 −2.27. HRMS (ESI-TOF) *m*/*z*: found 2086.2548, calc. for [M + H + NH_4_]^2+^ C_118_H_186_N_2_O_21_P_2_Si_2_ + NH_4_^+^ + H^+^: *m*/*z* = 2086.2527.

**6-*O*-Benzyl-4-*O*-[bis(benzyloxy)phosphoryl]-2-deoxy-2-[(*R*)-3-(dodecanoyloxy)-tetradecanoylamino]-β-d-glucopyranosyl-(1↔1)-6-*O*-benzyl-4-*O*-[bis(benzyloxy)-phosphoryl]-2-deoxy-2-[(*R*)-3-(dodecanoyloxy)tetradecanoylamino]-α-d-glucopyranoside (5).** To a cooled (0 °C) stirred solution of 4 (25 mg, 12 µmol) in dry THF (2 mL) in a Teflon tube, a solution of 3HF∙Py (25 µL, 120 µmol) in THF (70%) was added and the reaction mixture was stirred for 72 h at 0 °C. The mixture was then diluted with DCM (20 mL) and washed with satd. aq. NaHCO_3_ (pH 8, 3 × 5 mL) and water (2 × 5 mL). The organic phase was dried over Na_2_SO_4_, filtered over cotton, and concentrated. The residue was purified by HPLC (toluene–EtOAc, 2:1 → 1:4) to give 5 (15 mg, 68%). R_f_ = 0.4 (hexane-EtOAc, 2:1); α20D = 18 (*c* = 1, CHCl_3_). ^1^H NMR (600 MHz, CDCl_3_): δ 7.34–7.15 (m, 30H, Ph), 6.43 (d, *J*_NH′-2′_ = 7.2 Hz, NH′), 6.42 (d, 1H, *J*_NH-2_ = 8.3 Hz, NH), 5.19 (m, 1H, βC*H*^Myr^), 5.13 (m, 1H, βC*H*^Myr^), 5.07 (d,1H, *J*_1-2_ = 3.8 Hz, H-1), 5.04–4.95 (m, 8H, 2×OP(O)(OC*H_2_*Ph)_2_), 4.79 (d, 1H, *J*_1′-2′_ = 8.6 Hz, H-1′), 4.70 (bs, 0.7H, OH-3,3′), 4.39–4.30 (m, 5H, 2×C*H*_2_-Ph, H-4), 4.22 (ddd, 1H, *J*_4′-P′_ = *J*_3′-4′_ = 8.6 Hz, *J*_4′-5′_ = 8.3 Hz, H-4′), 4.14 (ddd, 1H, *J*_4-5_ = 10.1 Hz, *J*_5-6a_ = 5.2 Hz, *J*_5-6b_ = 1.6 Hz, H-5), 4.09 (ddd, 1H, *J*_2-3_ = 10.4 Hz, H-2), 4.05 (t, 1H, *J*_2′-3′_ = 9.4 Hz, H-3′), 3.92 (dd, 1H, *J*_3′-4′_ = 8.9 Hz, H-3), 3.62 (dd, 1H, *J*_6a-6b_ = 10.7 Hz, H-6b), 3.58–3.55 (m, 1H, H-2′), 3.55 (dd, 1H, *J*_6a′-6b′_ = 11.0 Hz, *J*_5′-6b′_ = 1.7 Hz, H-6b′), 3.51–3.50 (m, 1H, H-5′), 3.48 (dd, 1H, H-6a), 3.43 (dd, 1H, *J*_5′-6a′_ = 4.9 Hz, H-6a′), 2.56–2.36 (m, 4H, 2×αC*H*_2_^Myr^_,_), 2.26 (m, 4H, 2×αC*H*_2_^Lau^), 1.62–1.53 (m, 8H, βC*H*_2_^Lau^, γC*H*_2_^Myr^), 1.30–1.22 (m, 68H, C*H*_2_^Myr,Lau^), 0.88–0.86 (m, 12H, C*H*_3_^Myr;Lau^). ^13^C-NMR (150.9 MHz, CDCl_3_): δ 174.07, 173.77, 171.28, 171.23 (4C, 4×*C*=O), 137.86, 137.80, 135.84, 135.80, 135.38, 135.36 (6C, *C*_q_-Ph), 128.74, 128.68, 128.64, 128.60, 128.49, 128.42, 128.36, 128.30, 128.03, 127.99, 127.63, 127.57, 127.47 (30C, Ph), 99.37 (1C, C-1′), 96.80 (1C, C-1), 76.98 (1C, C-4), 76.83 (1C, C-4′), 74.04 (1C, C-5′), 73.40, 73.34 (2C, CH_2_-Bn), 72.32 (1C, C-3), 71.94 (1C, C-3′), 71.33 (1C, *C*H^Myr^), 70.99 (1C, *C*H^Myr^), 70.31 (1C, C-5), 70.14, 70.10, 69.97, 69.93, 69.76, 69.72 (6C, 2×*C*H_2_Ph, 2×OP(O)(O*C*H*_2_*Ph)_2_), 68.54 (1C, C-6), 68.32 (1C, C-6′), 57.03 (1C, C-2′), 53.35 (1C, C-2), 42.23, 41.33 (4C, 2×α*C*^Myr^, 2×α*C*^Lau^), 34.58, 34.41, 34.22, 31.92, 29.65, 29.54, 29.43, 29.35, 29.21, 25.37, 25.00, 24.96, 22.68 (*C*H_2_^Myr,Lau^), and 14.09 (4C, *C*H_3_^Myr,Lau^). ^31^P NMR (243 MHz, CDCl_3_): 0.03 0.77. HRMS (ESI-TOF) m/z: found 1858.0924, calc. for [M + H]^+^ C_106_H_158_N_2_O_21_P_2_ + H^+^: *m*/*z* = 1858.0905.

**6-*O*-Benzyl-2-deoxy-2-[(*R*)-3-(dodecanoyloxy)tetradecanoylamino]-β-d-glucopyranosyl-(1↔1)-6-*O*-benzyl-2-deoxy-2-[(*R*)-3-(dodecanoyloxy)tetradecanoylamino]-α-d-glucopyranoside (6).** To a stirred solution of 2 (90 mg, 60 µmol) in dry DCM (5 mL), 4 Å molecular sieves were added and the suspension was stirred for 2 h at r.t. under atmosphere of Ar. The mixture was then cooled to −70 °C and a solution of triethylsilane (74 µL, 460 µmol, 8 eq) in DCM (5% solution) and a solution of trifluoromethanesulfonic acid (41 µL, 460 µmol, 8 eq) in DCM (5%) were added successively. The stirring was continued for 3 h, the reaction was quenched by addition of a solution of Et_3_N in DCM (5%, 70 µL, 460 µmol, 8 eq), and the mixture was stirred for 15 min at −70 °C. The reaction mixture was brought up to r.t., then diluted with DCM (50 mL), and the solids were removed by filtration over a pad of Celite. The filtrate was washed with satd. aq. NaHCO_3_ (2 × 20 mL), water (20 mL), and brine (20 mL), dried over Na_2_SO_4_, filtered over cotton, and concentrated. The residue was purified by column chromatography on silica gel (toluene - EtOAc, 10:1 → 0:1) which gave 6 (52 mg, 65%). R_f_ = 0.3 (toluene-EtOAc, 1:1), α20D = 14 (*c* = 1, CHCl_3_). ^1^H NMR (600 MHz, CDCl_3_): δ 7.34–7.26 (m, 10H, Ph), 6.69 (d, 1H, NH′, *J*_2′,N′_ = 6.7 Hz), 6.49 (d, 1H, NH, *J*_2,N_ = 8.3 Hz), 5.21 (m, 1H, βC*H* ^Myr^), 5.15 (m, 1H, βC*H* ^Myr^), 5.07 (d, 1H, H-1, *J*_1,2_ = 3.5 Hz), 4.90 (d, 1H, H-1′, *J*_1,2_ = 8.4 Hz), 4.55–4.47 (m, 4H, C*H*_2_Ph), 4.02 (m, 3H, H-2, H-5, H-4′), 3.78 (dd, 1H, *J*_6a,6b_ = 2.1 Hz, *J*_5,6a_ = 10.3 Hz, H-6a), 3.69 (m, 2H, H-3, H-6a′), 3.63 (dd, 1H, *J*_5′,6a′_ = 10.4 Hz and *J*_6a′,6b′_ = 5.6 Hz, H-6b′), 3.54 (m, 2H, H-6b, H-5′), 3.45 (m, 3H, H-4, H-2′, H-3′), 2.89 (bs, 4H, 4×OH), 2.55-2.22 (m, 8H, m, 8H, 2×αC*H*_2_^Myr^, 2×αC*H*_2_^Lau^), 1.55 (m, 8H, βC*H*_2_^Lau^, γC*H*_2_^Myr^), 1.58–1.25 (m, 68H, C*H*_2_^Myr,Lau^), 0.88 (m, 12H, C*H*_3_^Myr,Lau^). ^13^C-NMR (150.9 MHz, CDCl_3_): δ 174.77, 174.36, 171.82 and 171.68 (4C, 4×*C*=O), 137.73 and 137.60 (2C, *C*_q_-Ph), 128.50, 127.88, 127.84, 127.66 and 127.61 (10C, Ph), 98.46 (1C, C-1′), 95.53 (1C, C-1), 74.44 (1C, H-4′), 74.20 (1C, H-4), 73.59 and 73.45 (2C, *C*H_2_Ph), 72.69 (1C, C-3′), 72.08 (1C, C-3), 71.78 and 71.48 (2C, *C*H_FA_), 71.44 (1C, C-5′), 71.38 (1C, C-5), 70.32 (1C, C-6′), 69.86 (1C, C-6), 57.38 (1C, C-2′), 53.28 (1C, C-2), 42.78, 41.68, 34.81, 34.59, 34.43, 31.93, 29.68, 29.66, 29.63, 29.60, 29.58, 29.57, 29.54, 29.53, 29.41, 29.37, 29.35, 29.33, 29.27, 29.20, 29.16, 25.37, 25.36, 24.96, 24.94 22.69 (40C, *C*H_2_^Myr,Lau^), and 14.10 (4C, *C*H_3_^Myr,Lau^). HRMS (ESI-TOF) *m*/*z*: found 1337.9700, calc. for [M + H]^+^ C_78_H_132_N_2_O_15_P_2_ + H^+^: *m*/*z* = 1337.9614.

**6-O-Benzyl-4-*O*-[bis(benzyloxy)phosphoryl]-3-*O*-[bis(benzyloxy)phosphoryl]-2-[(*R*)-3-(dodecanoyloxy)tetradecanoylamino]-2-deoxy-β-d-glucopyranosyl-(1↔1)-6-*O*-benzyl-4-*O*-[bis(benzyloxy)phosphoryl]-3-*O*-[bis(benzyloxy)phosphoryl]-2-[(*R*)-3-(dodecanoyloxy)tetradecanoylamino]-2-deoxy-β-d-glucopyranoside (7).** To a stirred solution of 6 (40 mg, 30 µmol) in dry DCM (4 mL) bisbenzyloxy(diisopropylamino)phosphine (52 mg, 51 µL, 150 µmol) and 1*H*-tetrazole (11 mg, 150 µmol, 0.45M in CH_3_CN) were added successively under atmosphere of Ar. The mixture was stirred for 1 h at r.t., then cooled to −78 °C, and a solution of *m*CPBA (21 mg, 122 µmol, 0.1 M in dry CH_2_Cl_2_) was added. The reaction mixture was stirred for 1 h at −78 °C, then quenched by addition of a solution of Et_3_N (30 µL) in MeOH (300 µL) and brought to r.t. The mixture was diluted with CH_2_Cl_2_ (30 mL) and washed with sat. aq. NaHCO_3_ (2 × 10 mL), water (10 mL), and brine (10 mL). The organic layer was dried over Na_2_SO_4_, filtered over cotton, and concentrated. The residue was purified by chromatography on silica gel (toluene – EtOAc, 2:3 → 0:1) and by gel permeation chromatography on Bio-Beads^®^ S-X1 support (600 × 1.5 mm, toluene—CHCl_3_, 3:1) to give 7 (55 mg, 78%). α20D = 11 (*c* = 1.2, CHCl_3_); R_f_ = 0.7 (hexane-EtOAc, 2:1). ^1^H NMR (600 MHz, CDCl_3_): δ 7.28–7.11 (m, 50H, Ph), 7.03 (d, 1H, NH′, *J*_2′,N′_ = 6.7 Hz), 6.75 (d, 1H, NH, *J*_2,N_ = 8.5 Hz), 5.25 (m, 1H, βC*H*^Myr^), 5.13 (m, 1H, βC*H*^Myr^), 5.10 (d, 1H, *J*_1,2_ = 3.5 Hz, H-1,), 5.07–4.85 (m, 8H, C*H*_2_OP(O)(OBn)), 4.80 (d, 1H, H-1′, *J*_1′,2′_ = 8.2 Hz), 4.74 (m, 2H, H-3, H-3′), 4.60 (q, 1H, *J*_3,4_ = *J*_4,5_ = *J*_4,P_ = 9.5 Hz, H-4), 4.52 (q, 1H, *J*_3′,4′_ = *J*_4′,5′_ = *J*_4′,P′_ = 9.4 Hz, H-4′), 4.43, 4.36, 4.30, 4.24 (d(x4), 4H, 2×C*H*_2_Ph), 4.30 (ddd, 1H, H-2), 4.25 (m, 1H, H-5), 3.73 (m, 1H, H-2′), 3.68 (m, 2H, H-6a and H-6a′), 3.64 (dd, 1H, *J*_5,6b_ = 1.7 Hz, *J*_6a,6b_ = 11.0 Hz, H-6b), 3.64 (dd, 1H, *J*_5,6b_ = 1.7 Hz, *J*_6a,6b_ = 11.0 Hz, H-6b), 3.59 (dd, 1H, *J*_5′,6b′_ = 4.9 Hz, *J*_6a′,6b′_ = 10.9 Hz, H-6′b), 3.53 (m, 1H, H-5), 2.58 (dd, 1H, αC*H*_2_^Myr^), 2.44 (dd, 1H, αC*H*_2_^Myr^), 2.33 (dd, 1H, αC*H*_2_^Myr^), 2.28–2.16 (m, 5HH, αC*H*_2_^Myr^, 2×αC*H*_2_^Lau^), 1.68–1.39 (m, 8H, γC*H_2_*^Myr^, βC*H_2_*^Lau^), 1.55–1.17 (m, 68H, C*H*_2_^Myr,Lau^), 0.87 (m, 12H, C*H*_3_^Myr,Lau^). ^13^C NMR (150.9 MHz, CDCl_3_): δ 173.18, 173.00, 171.62 and 170.43 (4C, 4xC=O), 138.29 and 138.11 (2C, *C*_q_-Ph), 130.14, 129.79, 128.55, 128.48, 128.46, 128.42, 128.40, 128.36, 128.34, 128.31, 128.25, 128.23, 128.18, 128.13, 128.06, 128.04, 127.98, 127.95, 127.54 and 127.32 (30C, Ph), 99.68 (1C, C-1′), 98.12 (1C, C-1), 77.49 (1C, C-3), 77.05 (1C, C-3′), 74.20(1C, H-4′), 74.05 (1C, H-4), 73.81 (1C, C-5′), 73.13 and 72.99 (2C, *C*H_2_Ph), 71.08 (1C, C-5), 70.88 and 70.47 (2C, *C*H_FA_), 69.97, 69.77, 69.72 and 69.57 (4C, *C*H_2_PhOP(O)(OBn)), 68.19 (1C, C-6′), 67.86 (1C, C-6), 56.51 (1C, C-2′), 52.31 (1C, H-2), 34.56, 34.46, 31.94, 31.93, 29.73, 29.70, 29.66, 29.59, 29.50, 29.42, 29.39, 29.37, 29.30, 29.28, 25.30, 25.07, 25.04, 22.69 (40C, *C*H_2_^Myr,Lau^), 14.11 (4C, *C*H_3_^Myr,Lau^). ^31^P NMR (243 MHz, CDCl_3_): −0.30, −0.33, −1.89 and −1.99. HRMS (ESI-TOF) *m*/*z*: found 2378.2011, calc. for [M + H]^+^ C_134_H_184_N_2_O_27_P_4_ + H^+^: *m*/*z* = 2378.2007.

**6-*O*-Benzyl-2-deoxy-2-[(*R*)-3-(dodecanoyloxy)tetradecanoylamino]-β-d-glucopyranosyl-(1↔1)-6-*O*-benzyl-3-*O*-*tert*-butyldimethylsilyl-2-deoxy-2-[(*R*)-3-(dodecanoyloxy)tetradecanoylamino]-α-d-glucopyranoside (8).** To a stirred solution of 2 (105 mg, 67 µmol) in dry DCM (15 mL), molecular sieves 4 Å were added and the suspension was stirred for 2 h in an atmosphere of Ar. The mixture was then cooled to −65 °C and a solution of triethylsilane (20 mg, 27 µL, 170 µmol, 2.5 eq) in dry DCM (5% solution) followed by a solution of trifluoromethanesulfonic acid (25 mg, 15 µL, 170 µmol, 2.5 eq) in dry DCM (5% solution) were added. The reaction mixture was stirred at −65 °C for 3 h under atmosphere of Ar, and then quenched by addition of a solution of Et_3_N (30 µL, 200 µmol, 3 eq) in DCM (5% solution), and the stirring was continued for 15 min at −65 °C. The mixture was diluted with DCM (30 mL), the solids were removed by filtration over a pad of Celite, the filtrate was washed with satd. aq. NaHCO_3_ (30 mL), water (30 mL), and brine (30 mL), dried over Na_2_SO_4_, filtered, and concentrated. The residue was purified by column chromatography on silica gel (toluene-EtOAc, 10:1 → 4:1) which gave 8 (68 mg, 71%). R_f_ = 0.4 (toluene - EtOAc, 1:1), α25D = 12 (*c* = 1, CHCl_3_). ^1^H NMR (600 MHz, CDCl_3_): δ 7.35–7.27 (m, 10H, Ph), 6.69 (d, 1H, NH′), 5.83 (d, 1H, NH), 5.19–5.14 (m, 1H, βC*H*^Myr^), 5.12 (d, 1H, H-1), 5.11–5.06 (m, 1H, βC*H*^Myr^), 4.97 (d, 1H, H-1′), 4.54–4.47 (m, 4H, C*H*_2_-Bn), 4.11 (t, 1H, *J*_3′-4′_ = 9.7 Hz, H-3′), 4.08 (dt, 1H, *J*_2-3_ = *J_2-NH_*= 9.6 Hz, *J*_1-2_ = 3.7 Hz, H-2), 4.04 (ddd, 1H, *J*_4-5_ = 9.6 Hz, *J*_5-6b_ = 2.8 Hz, *J*_5-6a_ = 6.4 Hz, H-5), 3.76 (dd, 1H, *J*_5′-6a′_ = 10.3 Hz, *J*_6a′-6b′_ = 3.0 Hz, H-6a′), 3.69–3.64 (m, 3H, H-6b′, H-6a, H-3), 3.56–3.54 (m, 2H, H-6b, H-5′), 3.45 (t, 1H, *J*_4′-5′_ = 9.2 Hz, H-4′), 3.40 (dd,1H, *J*_4-3_ = 8.9 Hz, H-4), 3.31 (ddd, 1H, *J*_2′-3′_ = 9.9 Hz, *J*_2′-NH′_ = 6.7 Hz, *J*_1′-2′_ = 8.6 Hz, H-2′), 2.55–2.20 (m, 8H, 2×αC*H*_2_^Myr^, 2×αC*H*_2_^Lau^), 1.69–1.52 (m, 8H, 2×βC*H*_2_^Lau^, 2×γC*H*_2_^Myr^), 1.32–1.16 (m, 68 H, C*H*_2_^Myr,Lau^), 0.91–0.84 (m, 21H, CH_3_^Myr,Lau^, *t*BuSi), 0.10, 0.06 (2s, 6H, SiMe). ^13^C NMR (150.9 MHz, CDCl_3_): δ 174.78, 173.29, 171.45, 169.60 (4C, 4×*C*=O), 137.85, 137.48 (2C, *C*_q_-Ph), 128.56, 128.48, 128.00, 127.83, 127.76, 127.69, 127.62 (10C, Ph), 98.20 (1C, C-1′), 95.72 (1C, C-1), 74.32 (1C, C-5′), 73.62, 73.56 (2C, *C*H_2_-Bn), 72.62 (1C, C-3′), 72.56 (1C, C-4), 72.22 (1C, C-4′), 71.92 (1C, *C*H^Myr^), 71.42 (1C, *C*H^Myr^), 71.22 (1C, C-5), 70.42 (1C, C-6′), 70.34 (1C, C-6), 58.04 (1C, C-2′), 53.40 (1C, C-2), 42.83, 41.08 (4C, 2×α*C*^Myr^, 2×α*C*^Lau^), 34.96, 34.63, 34.61, 34.37, 31.92, 29.62, 29.59, 29.58, 29.56, 29.54, 29.52, 29.45, 29.36, 29.35, 29.27, 29.24, 29.16 (*C*H_2_^FA^)_,_25.86, 25.79 (3C, *t*BuSi), 25.35, 25.28, 25.08, 22.69 (*C*H_2_^Myr,Lau^)_,_ 14.09 (4C, *C*H_3_^Myr,Lau^), −4.00, −4.32 (2C, SiMe). HRMS (ESI-TOF) *m*/*z*: found 1452.0485, calc. for [M + H]^+^ C_84_H_146_N_2_O_15_Si + H^+^: *m*/*z* = 1452.0477.

**6-*O*-benzyl-3,4-*O*-[bis(benzyloxy)phosphoryl]-2-deoxy-2-[(*R*)-3-(dodecanoyloxy)-tetradecanoylamino]-β-d-glucopyranosyl-(1↔1)-6-*O*-benzyl-4-*O*-[bis(benzyloxy)phosphoryl]-3-*O*-*tert*-butyldimethylsilyl-2-deoxy-2-[(*R*)-3-(dodecanoyloxy)tetradecanoylamino]-α-d-glucopyranoside (9)**. To a stirred solution of 8 (28 mg, 20 µmol) in dry DCM (5 mL), bisbenzyloxy(diisopropylamino)phosphine (32 mg, 31 µL, 90 µmol) and a solution of 1*H*-tetrazol (16 µL, 116 µmol, 0.45M in CH_3_CN) were added successively and the stirring was continued for 2 h at r.t. in an atmosphere of Ar. The reaction mixture was cooled to −78 °C, a solution of *m*CPBA (15 mg, 80 µmol) in DCM (0.6 mL) was added, and the stirring was continued for 2h at −78 °C. The reaction mixture was neutralized by addition of Et_3_N (50 µL, 36 µmol, 18 eq), stirred for 10 min at −78 °C, then brought to r.t. The mixture was diluted with DCM (20 mL) and washed with satd. aq. NaHCO_3_ (2 × 5 mL), water (10 mL), and brine (10 mL). The organic phase was dried over Na_2_SO_4_, filtered over cotton, and concentrated. The residue was purified by column chromatography on silica gel (toluene/EtOAc, 8:1 → 1:1) to give 9 (30 mg, 69%). R_f_ = 0.7 (toluene-EtOAc, 1:1); α25D = 16 (*c* = 1, CHCl_3_). ^1^H NMR (600 MHz, CDCl_3_): δ 7.28–7.11 (m, 40H, Ph), 7.10 (d, 1H, *J*_2′-NH′_ = 7.5 Hz, NH′), 6.17 (d, 1H, *J*_2-NH_ = 10.2 Hz, NH), 5.27–5.21 (m, 2H, 2×βC*H*^Myr^), 5.05–4.82 (m, 12 H, 3×OP(O)(OC*H_2_*Ph)_2_), 4.90 (m, 1H, H-1), 4.58 (d, 1H, *J*_1′-2′_= 8.3 Hz, H-1′), 4.51 (m, 1H, H-3′), 4.4 (m, 1H, H-4′), 4.45 (d, 1H, OC*H_2_*Ph), 4.43 (d, 1H, OC*H_2_*Ph), 4.35 (dd, 1H, *J*_3-4_ = *J*_4-5_= 9.3 Hz, *J*_4-P_ = 9.5 Hz, H-4), 4.30 (d, 1H, OC*H_2_*Ph),4.28 (d, 1H, OC*H_2_*Ph), 4.23 (ddd, 1H, *J*_2-NH_ = *J*_2-3_ = 9.9 Hz, *J*_1-2_ = 3.1 Hz, H-2), 4.18 (ddd, 1H, *J*_5-6a_ = 4.2 Hz, *J*_5-6b_ = 2.0 Hz, H-5), 3.94 (dd, 1H, H-3), 3.85 (ddd, 1H, *J*_2′-NH′_ = 10.3 Hz, *J*_2′-3′_ = 6.8 Hz, H-2′), 3.79 (dd, 1H, *J_6a-6b_* = 10.7 Hz, H-6a), 3.70 (dd, 1H, *J_6a_*_′*-6b*′_ = 10.9 Hz, H-6b′), 3.68 (dd, 1H, H-6b), 3.61 (dd, 1H, H-6a′), 3.49 (ddd, 1H, *J*_4′-5′_ = 9.1 Hz, *J*_5′-6b′_ = 2.1 Hz, *J*_5′-6a′_ = 4.6 Hz, H-5′), 2.61–2.17 (m, 8H, 2×αC*H*_2_^Myr^, 2×αC*H*_2_^Lau^), 1.68–1.47 (m, 8H, 2×βC*H*_2_^Lau^, 2×γC*H*_2_^Myr^), 1.30–1.22 (m, 68H, C*H*_2_^Myr,Lau^), 0.89–0.86 (m, 12H, C*H*_3_^Myr,Lau^), 0.81 (s, 9H, *t*BuSi), 0.11, 0.08 (2s, 6H, SiMe). ^13^C NMR (150.9 MHz, CDCl_3_): δ 172.95, 172.86, 172.05, 169.50 (4C, 4×*C*=O), 138.43, 138.06, 135.94, 135.89, 135.62, 135.58, 135.36, 135.31 (8C, *C*_q_-Ph), 128.60, 128.56, 128.51, 128.47, 128.45, 128.37, 128.35, 128.32, 128.18, 128.15, 128.06, 128.02, 127.96, 127.93, 127.56, 127.51, 127.44, 127.23 (40C, Ph), 100.53 (1C, C-1′), 98.86 (1C, C-1), 76.93 (1C, C-4′), 76.89 (1C, C-4), 74.00 (1C, C-5′), 73.86 (1C, C-3′), 73.14, 72.76 (2C, 2×*C*H_2_-Ph), 71.59 (1C, C-5), 71.32 (1C, C-3), 70.45 (2C, *C*H^Myr^), 70.11, 70.08, 70.05, 69.79, 69.76, 69.72, 69.68, 69.64, 69.19, 69.15 (6C, 3×OP(O)(O*C*H*_2_*Ph)_2_), 68.37, 68.25 (2C, C-6, C-6′), 56.05 (1C, C-2′), 52.80 (1C, C-2), 41.39, 40.68 (4C, 2×α*C*^Myr^, 2×α*C*^Lau^), 34.78, 34.65, 34.59, 34.41, 31.93, 29.70, 29.68, 29.69, 29.62, 29.58, 29.54, 29.43, 29.37, 29.30 (*C*H_2_^Myr,Lau^)_,_ 25.92 (3C, *t*BuSi), 25.48, 25.38, 25.05, 22.68 (*C*H_2_^Myr,Lau^)_,_ 14.10 (4C, *C*H_3_^Myr,Lau^),−3,66, −4.04 (2C, SiMe). ^31^P NMR (243 MHz, CDCl_3_): δ 0.49, −1.80 and −1.84. HRMS (ESI-TOF) *m*/*z*: found 2232.2340, calc. for [M + H]^+^ C_126_H_185_N_2_O_24_P_3_Si + H^+^: *m*/*z* = 2232.2372.

**6-*O*-Benzyl-3,4-di-*O*-[bis(benzyloxy)phosphoryl]-2-deoxy-2-[(*R*)-3-(dodecanoyloxy)tetradecanoylamino]-β-d-glucopyranosyl-(1↔1)-6-*O*-benzyl-4-*O*-[bis(benzyloxy)phosphoryl]-2-deoxy-2-[(*R*)-3-(dodecanoyloxy)tetradecanoylamino]-α-d-glucopyranoside (10)** and **6-*O*-Benzyl-3,4-di-*O*-[bis(benzyloxy)phosphoryl]-2-deoxy-2-[(*R*)-3-(dodecanoyloxy)tetradecanoylamino]-β-d-glucopyranosyl-(1↔1)-6-*O*-benzyl-3-*O*-[bis(benzyloxy)phosphoryl]-2-deoxy-2-[(*R*)-3-(dodecanoyloxy)tetradecanoylamino]-α-d-glucopyranoside (11)**. To a cooled (0 °C) stirred solution of **9** (30 mg, 13 µmol) in dry THF (2 mL) in a Teflon tube, a solution of TBAF in THF (1 M, 15 µL, 14 µmol) was added at 0 °C and the mixture was stirred for 12 at 0 °C. The mixture was diluted with DCM (10 mL) and washed with satd. aq. NaHCO_3_ (3 × 5 mL), water (5 mL), and brine (5 mL). The organic phase was dried over Na_2_SO_4_, filtered over cotton, and concentrated. The residue was purified by column chromatography on silica gel (toluene/EtOAc, 2:1 → 1:1) and by gel permeation chromatography on Bio-Beads^®^ S-X1 support (toluene-chloroform, 3:1) to give **10** (10 mg, 37%) and **11** (9 mg, 33%). **10**: R_f_ = 0.4 (toluene-EtOAc, 1:1); α20D = 7 (*c* = 1, CHCl_3_); ^1^H NMR (600 MHz, CDCl_3_): δ 7.31–7.10 (m, 40H, Ph), 7.06 (d, 1H, *J*_NH′-2′_ = 7.6 Hz, NH′), 6.81 (d, 1H, NH), 5.30 (m, 1H, βC*H*^Myr^), 5.07 (m, 1H, βC*H*^Myr^), 5.07–4.08 (m, 6 H, OP(O)(OC*H_2_*Ph)_2_), 4.95 (d, 1H, *J*_1-2_ = 3.6 Hz, H-1), 4.93–4.84 (m, 6 H, OP(O)(OC*H_2_*Ph)_2_), 4.49 (dd, 1H, *J_4_*_′*-3*′_ = 9.0 Hz, H-4′), 4.42 (d, 1H, *J*_1′-2′_ = 8.2 Hz, H-1′), 4.41–4.20 (m, 4H, 2×C*H*_2_-Ph), 4.37 (dd, 1H, *J*_2′-3′_ = 11.3 Hz, *J*_3′-4′_ = 9.0 Hz, H-3′), 4.18 (ddd, 1H, *J*_4-5_ = 10.2 Hz, *J*_5-6a_ = 4.0 Hz, *J*_5-6b_ = 1.8 Hz, H-5), 4.12 (ddd, 1H, *J*_1-2_ = 3.6 Hz, *J*_2-3_ = 10.3 Hz, *J*_2-NH_ = 6.8 Hz, H-2), 3.99–3.94 (m, 1H, H-2′, H-3), 3.62 (dd, 1H, *J*_6′a-6′b_ = 10.9 Hz, H-6′b), 3.57 (dd, 1H, *J*_6a-6b_ = 11.0 Hz, H-6b), 3.53 (dd, 1H, H-6a′), 3.51 (dd, 1H, H-6a), 3.43 (ddd, 1H, *J*_4′-5′_ = 9.4 Hz, *J*_5′-6a′_ = 4.7 Hz, *J*_5′-6b′_ = 2.0 Hz, H-5′), 2.69–2.20 (m, 8H, 2×αC*H*_2_^Myr^, 2×αC*H*_2_^Lau^), 1.66–1.44 (m, 8H, 2×βC*H*_2_^Lau^, 2×γC*H*_2_^Myr^), 1.30–1.13 (m, 68H, C*H*_2_^Myr,Lau^), 0.89–0.86 (m, 12H, C*H*_3_^Myr,Lau^). ^13^C NMR (150.9 MHz, CDCl_3_): δ 173.62, 172.90, 172.17, 172.08 (4C, 4×*C*=O),138.18, 138.00, 136.15, 136.10, 135.99, 135.53, 135.13, 135.08 (8C, *C*_q_-Ph), 128.66, 128.59, 128.55, 128.46, 128.44, 128.37, 128.33, 128.27, 128.19, 128.17, 128.06, 128.01, 127.98, 127.96, 127.60, 127.58, 127.51, 127.36 (40C, Ph), 100.53 (1C, C-1′), 98.61 (1C, C-1), 77.60 (1C, C-3′), 76.93 (1C, C-4′), 74.17 (1C, C-5′), 73.56 (1C, C-4), 73.29, 73.09 (2C, 2×CH_2_-Ph), 73.16 (1C, C-3), 71.33 (1C, C*H*^Myr^), 70.73 (1C, C*H*^Myr^), 70.50 (1C, C-5), 70.16, 70.17, 69.80, 69.77, 69.74, 69.66, 69.62, 69.49, 69.46 (6, 3xOP(O)(O*C*H*_2_*Ph)_2_, 68.18 (1C, C-6′), 67.91 (1C, C-6), 55.59 (1C, C-2′), 53.36 (1C, C-2), 41.91, 41.08 (4C, 2×α*C*^Myr^, 2×α*C*^Lau^), 34.39, 34.56, 34.52, 34.25, 31.94, 29.72, 29.68, 29.65, 29.63, 29.58, 29.55, 29.43, 29.37, 29.27, 25.41, 25.30, 25.10, 25.01, 22.69 (*C*H_2_^Myr,Lau^), 14.11 (4C, *C*H_3_^Myr,Lau^). ^31^P NMR (243 MHz, CDCl_3_): δ 0.68, −1.21, −1.89. HRMS (ESI-TOF) *m*/*z*: found 2140.1301, calc. for [M + Na]^+^ C_120_H_171_N_2_O_24_P_3_ + Na^+^: *m*/*z* = 2140.1327.

**11**. R_f_ = 0.5 (toluene-EtOAc, 1:1); α20D = 8.0 (*c* = 1, CHCl_3_); ^1^H NMR (600 MHz, CDCl_3_): δ 7.36–7.13 (m, 40H, Ph), 7.13 (d, 1H, NH′), 6.49 (d, 1H, *J*_NH-2_ = 9.2 Hz, NH), 5.21–5.15 (m, 2H, 2×βC*H*^Myr^), 5.11–4.84 (m, 12 H, 3×OP(O)(OC*H_2_*Ph)_2_), 5.00 (d, 1H, H-1), 4.59 (d, 1H, *J*_1′-2′_ = 8.2 Hz, H-1′), 4.54 (m, 1H, H-3′), 4.51 (m, 1H, H-4′), 4.45–4.29 (m, 5H, 2×C*H*_2_-Ph, H-3), 4.34 (ddd, 1H, *J*_NH-2_ = 9.2 Hz, *J*_2-3_ = 9.6 Hz, *J*_1-2_ = 3.6 Hz, H-2), 4.12 (m, 1H, H-5), 3.86 (ddd, 1H, *J*_1′-2′_ = 8.2 Hz, *J*_2′-3′_ = 9.0 Hz, *J*_2′-NH′_ = 7.6 Hz, H-2′), 3.80 (dd, 1H, *J*_3-4_ = 9.0 Hz, *J*_4-5_ = 9.8 Hz, H-4), 3.70 (dd, 1H, *J*_5-6a_ = 10.9 Hz, *J*_6a-6b_ = 1.9 Hz, H-6a), 3.62 (dd, 1H, *J_5_*_′*-6*′*a*_ = 10.8 Hz, *J*_6′a-6′b_ = 2.3 Hz, H-6′a), 3.59 (dd, 1H, *J*_5-6b_ = 4.8 Hz, H-6b), 3.58 (dd, 1H, *J*_5′-6b′_ = 4.7 Hz, H-6b′), 3.51–3.49 (m, 1H, H-5′), 2.56–2.15 (m, 8H, 2×αC*H*_2_^Myr^, 2×αC*H*_2_^Lau^), 1.66–1.48 (m, 8H, 2×βC*H*_2_^Lau^_,_ 2×γC*H*_2_^Myr^), 1.33–1.11 (m, 68H, C*H*_2_^Myr,Lau^), 0.89–0.86 (m, 12H, C*H*_3_^Myr,Lau^). ^13^C NMR (150.9 MHz, CDCl_3_): δ 173.01, 172.98, 172.00, 169.98 (4C, 4×*C*=O), 138.10, 138.08, 135.64, 135.62, 135.60, 135.38, 135.34 (8C, *C*_q_-Ph), 128.69, 128.63, 128.61, 128.58, 128.52, 128.50, 128.42, 128.39, 128.28, 128.20, 128.12, 128.05, 128.00, 127.98, 127.96, 127.60, 127.54, 127.51 (40C, Ph), 100.00 (1C, C-1′), 98.36 (1C, C-1), 81.20 (1C, C-3), 77.18 (1C, C-3′), 74.12 (1C, C-5′), 73.77 (1C, C-4′), 73.48, 73.15 (2C, 2×*C*H_2_-Ph), 71.96 (1C, C-5), 70.86, 70.65 (2C, *C*H^Myr^), 70.11, 70.07, 70.02, 69.98, 69.79, 69.76, 69.72, 69.69 (6C, 3×OP(O)(O*C*H*_2_*Ph)_2_), 69.55 (1C, C-4), 68.81 (1C, C-6′), 68.17 (1C, C-6), 55.94 (1C, C-2′), 50.92 (1C, C-2), 41.87, 40.43 (4C, 2×α*C*^Myr^, 2×α*C*^Lau^), 34.67, 34.58, 34.53, 33.33, 31.93, 29.74, 29.69, 29.58, 29.53, 29.46, 29.42, 29.41, 29.39, 29.37, 29.31, 29.28 25.39, 25.28, 25.09, 25.03, 22.69 (*C*H_2_^Myr,Lau^), 14.12 (4C, *C*H_3_^Myr,Lau^). ^31^P NMR (243 MHz, CDCl_3_): δ 0.59, 0.41, −1.87. HRMS (ESI-TOF) *m*/*z*: found 2140.1335, calc. for [M + Na]^+^ C_120_H_171_N_2_O_24_P_3_ + Na^+^: *m*/*z* = 2140.1327.

**2-Deoxy-2-[(*R*)-3-(dodecanoyloxy)tetradecanoylamino]-4,3-di-*O*-(phosphoryl)-β-d-glucopyranosyl-(1↔1)-2-[(*R*)-3-(dodecanoyloxy)tetradecanoylamino]-3-*O*-(phosphoryl)-α-d-glucopyranoside (DLAM29)**. To a stirred solution of 11 (6 mg, 3 µmol) in toluene, MeOH (1:1, 3 mL) Pd black (10 mg) was added. The vessel was purged with Ar, the atmosphere was exchanged to hydrogen (3×), and the vessel was filled with hydrogen. The mixture was stirred for 22 h at r.t., then diluted with toluene-MeOH (1:1, 5 mL), and the solids were removed by filtration over a pad of Celite. The filtrate was concentrated and the residue was purified by gel permeation chromatography on Bio-Beads^®^ S-X1 support (toluene–chloroform-MeOH, 1:4:2) to give DLAM29 (4 mg, 95%). ^1^H NMR (600 MHz, CDCl_3_-MeOD, 2:1): δ 5.28 (m, 1H, βC*H*^Myr^), 5.20 (m, 1H, βC*H*^Myr^), 4.94 (d, 1H, *J*_1,2_ = 3.2 Hz, H-1), 4.64 (d, 1H, *J*_1′,2′_ = 8.2 Hz, H-1′), 4.43 (m, 1H, H-3′), 4.28 (m, 1H, H-3), 4.12 (m, 1H, H-4′), 4.08 (m, 2H, H-5, H-2), 3.85–3.70 (m, 5H, H-2′, H-6a,b, H-6′a,b), 3.60 (m, 1H, H-5′), 2.63-2.52 (m, 3H, αC*H_2_*^Myr^), 2.42 (dd, 1H, αC*H_2_*^Myr^), 2.30–2.22 (m, 4H, αC*H_2_*^Lau^), 1.63–1.53 (m, 8H, γC*H_2_*^Myr^, βC*H_2_*^Lau^), 1.63–1.55 (m, 68H, C*H*_2_^Myr,Lau^), 0.84 (m, 12H, C*H_3_*^Myr,Lau^). 2.63–2.52 (m, (m, 8H, αC*H*_2_^Myr,Lau^), 1.57–1.23 (m, 72H, C*H*_2_^Myr,Lau^), 0.84 (m, 12H, C*H_3_*^Myr,Lau^). ^31^P NMR (243 MHz, CDCl_3_/MeOD 2:1): δ 1.06, 0.79, 0.81. MALDI-TOF-MS: found *m*/*z* [M − H]^−^ 1395.7664, calc. for C_64_H_123_N_2_O_24_P_3_ − H^−^ [M − H]^−^ 1395.7656.

**2-Deoxy-2-[(*R*)-3-(dodecanoyloxy)tetradecanoylamino]-β-d-glucopyranosyl-(1↔1)-2-deoxy-[(*R*)-3-(dodecanoyloxy)tetradecanoylamino]-α-d-glucopyranoside 3′, 4′, 4-trisphosphate (DLAM30)**. To a stirred solution of 10 (7 mg, 4 µmol) in dry toluene-MeOH (1:1, 3 mL), Pd black (10 mg) was added. The vessel was purged with Ar (3×) and then filled with hydrogen. The mixture was stirred for 20 h at r.t., diluted with toluene-MeOH, 1:1, the solids were removed by filtration through a syringe filter (regenerated cellulose, 0.45 µm), and the solution was concentrated. The residue was purified by gel permeation chromatography on Bio-Beads^®^ S-X1 support (toluene-chloroform-MeOH, 1:4:2), which afforded DLAM30 (5 mg, 92%). ^1^H NMR (600 MHz, CDCl_3_-MeOD, 2:1): δ 5.26 (m, 1H, βC*H*^Myr^), 5.14 (m, 1H, βC*H*^Myr^), 4.91 (d, 1H, *J*_1,2_ = 3.4 Hz, H-1), 4.52 (d, 1H, *J*_1′,2′_ = 8.1 Hz, H-1′), 4.33 (dd, 1H, H-3′), 4.14 (dd, 1H, *J*_3′,4′_ = *J*_4′,5′_ = *J*_4′,P′_ = 9.5 Hz, H-4′), 4.10 (m, 1H, H-5), 3.98 (t, 1H, *J*_2,3_ = *J*_3,4_ = 9.4 Hz, H-4), 3.95 (dd, 1H, H-2), 3.86 (dd, 1H, H-2′), 3.80 (m, 1H, H-3), 3.78–3.65 (m, 4H, H-6a,b, H-6′a,b), 3.43 (m, 1H, H-5′), 2.61–2.50 (m, 4H, 2×αC*H_2_*^Myr^), 2.40 (dd, 1H, αC*H_2_*^Lau^), 2.25 (m, 3H, αC*H_2_*^Lau^), 1.56–1.49 (m, 8H, γC*H_2_*^Myr^, βC*H_2_*^Lau^), 1.57–1.23 (m, 68H, C*H*_2_^Myr,Lau^), and 0.84 (m, 12H, C*H_3_*^Myr,Lau^). ^31^P NMR (243 MHz, CDCl_3_/MeOD 2:1): δ 1.12, 0.73, 0.43. MALDI-TOF-MS: found *m*/*z* [M − H]^-^ 1395.6785, calc. for C_64_H_123_N_2_O_24_P_3_ − H^−^ [M − H]^−^ 1395.7656.

**2-Deoxy-2-[(*R*)-3-(dodecanoyloxy)tetradecanoylamino]-β-d-glucopyranosyl-(1↔1)-2-deoxy-2-[(*R*)-3-(dodecanoyloxy)tetradecanoylamino]-α-d-glucopyranoside 4, 4′-bisphosphate (DLAM33)**. To a stirred solution of **5** (10 mg, 5 µmol) in dry toluene-MeOH (2:1, 3 mL), Pd black (10 mg) was added. The vessel was purged with Ar and then filled with hydrogen. The mixture was stirred for 20 h at r.t., diluted with toluene–MeOH, 1:1, the solids were removed by filtration through a syringe filter (regenerated cellulose, 0.45 µm), and the solvent was removed. The residue was purified by gel permeation chromatography on Bio-Beads^®^ S-X1 support (toluene-chloroform-MeOH, 1:4:2), which afforded **DLAM33** (5 mg, 80%). ^1^H NMR (600 MHz, CDCl_3_/MeOD, 3:1): δ 5.24 (m, 1H, C*H*^Myr^), 5.16 (m, 1H, C*H*^Myr^), 4.90 (m, 1H, H-1), 4.53 (m, 1H, H-1′), 4.07 (m, 1H, H-4), 3.95 (m, 3H, H-2, H-3, H-4′), 3.78–3.74 (m, 6H, H-6a,b, H-6′a,b, H-5, H-3′), 3.66 (m, 1H, H-2′), 3.35 (m, 1H, H-5′), 2.51 (m, 2H, αC*H_2_*^Myr^), 2.46 (m, 1H, αC*H_2_*^Myr^), 2.38 (m, 1H, αC*H_2_*^Myr^), 2.15 (m, 4H, αC*H_2_*^Lau^), 1.53 (m, 8H, γC*H_2_*^Myr^, βC*H_2_*^Lau^), 1.57–1.22 (m, 68H, C*H*_2_), and 0.84 (m, 12H, C*H_3_*^Myr,Lau^). ^31^P NMR (243 MHz, CDCl_3_/MeOD 3:1): δ 1.43, 1.24. MALDI-TOF-MS: found *m*/*z* [M − H]^−^ 1315.6474, calc. for C_64_H_122_N_2_O_21_P_2_-H^-^ [M − H]^−^ 1315.7994.

**2-Ddeoxy-2-[(*R*)-3-(dodecanoyloxy)tetradecanoylamino]-β-d-glucopyranosyl-(1↔1)-2-deoxy-2-[(*R*)-3-(dodecanoyloxy)tetradecanoylamino]-α-d-glucopyranoside 3,3′,4,4′-tetraphosphate (DLAM36)**. To a stirred solution of **7** (10 mg, 4 µmol) in dry toluene-MeOH (1:1, 3 mL), Pd black (10 mg) was added. The vessel was purged with Ar and then filled with hydrogen. The mixture was stirred for 36 h at r.t., diluted with toluene-MeOH (1:2, 5 mL), the solids were removed by filtration through a syringe filter (regenerated cellulose, 0.45 µm), and the solvent was removed. The residue was purified by gel permeation chromatography on Bio-Beads^®^ S-X1 support (toluene-chloroform-MeOH, 1:4:2), which afforded **DLAM36** (5 mg, 87%). ^1^H NMR (600 MHz, CDCl_3_/MeOD 3:1): δ 5.32 (m, 1H, βC*H*^Myr^), 5.24 (m, 1H, βC*H*^Myr^), 4.98 (d, 1H, *J*_1,2_ = 3.4 Hz, H-1), 4.60 (d, 1H, *J*_1′,2′_ = 8.5 Hz, H-1′), 4.42 (q, 1H, *J*_2,3_ = *J*_3,4_ = *J*_3,P_ = 9.4 Hz, H-3), 4.36 (q, 1H, *J*_2,3_ = *J*_3,4_ = *J*_3,P_ = 9.8 Hz, H-3′), 4.17 (m, 4H, H-4, H-4′, H-5, H-2), 3.80 (m, 5H, H-2′, H-6a,b, H-6′a,b), 3.41 (m, 1H, H-5′), 2.58–2.53 (m, 3H, αC*H_2_*^Myr^), 2.45 (dd, 1H, αC*H_2_*^Myr^), 2.38 (m, 1H, αC*H_2_*^Myr^), 2.30 (t, 2H, αC*H_2_*^Lau^), 2.25 (t, 2H, αC*H_2_*^Lau^), 1.60 (m, 8H, γC*H_2_*^Myr^, βC*H_2_*^Lau^), 1.60–1.24 (m, 68H, C*H*_2_), and 0.86 (m, 12H, C*H_3_*^Myr,Lau^). ^31^P NMR (243 MHz, CDCl_3_/MeOD 3:1): δ 1.23, 1.01, 1.05, 0.86. MALDI-TOF-MS: found *m*/*z* 1475.5743 [M − H]^−^, calc. for C_64_H_124_N_2_O_27_P_4_ − H^−^ 1475.7318 [M − H]^−^.

### 3.2. Immunobiology

**Inhibition of cytokine induction in LPS-stimulated transient hTLR4/hMD-2/hCD-14 t or mTLR4/mMD-2 transfected HEK293 cells with αβ-DLAMs**.

HEK293 cells (DSMZ, Braunschweig, Germany) were transfected for 24 h with plasmids coding for human TLR4 (kind gift of P. Nelson, Seattle, WA, USA), human MD-2 (kind gift of K. Miyake, Tokyo, Japan), human CD14, mouse TLR4, or mouse MD-2 (kind gift of D. Golenbock, Worcester, MA, USA), using Lipofectamin2000 (Invitrogen GmbH, Karlsruhe, Germany) according to the manufacture’s instruction. Solutions of DLAMs **1**–**7** were prepared from stock solutions in DMSO (1 mg/mL) by dilutions using DMEM cell medium supplemented with 10% FCS. Next, transiently transfected cells were stimulated with increasing concentrations of antagonists or *E. coli* O111:B4 LPS (a kind gift of Otto Holst, Research Center Borstel) for 20 h. Recombinant human TNF-α (kind gift of D. Männel, Regensburg, Germany) served as transfection-independent control. IL-8 production was measured by human IL-8 CytoSet ELISA (Invitrogen) according to the manufacturer’s instruction. Data shown are combined from n = 3 independent experiments, error bars indicate standard error of the mean.

**Suppression of LPS-induced activation of human mononuclear cells (MNC).**

MNC (peripheral human blood mononuclear cells) were prepared from heparinized blood from healthy volunteers by gradient centrifugation (Biocoll, Merck) and were subsequently incubated in 96-well tissue culture plates at a volume of 150 µL and a concentration of 1 × 10^6^/mL using as medium RPMI-1640 supplemented with 100 U/mL penicillin (PAA Laboratories, Pasching, Austria), 100 µg/mL streptomycin (PAA Laboratories GmbH), and 10% FCS (Merck Millipore, Darmstadt, Germany). Solutions of antagonists were prepared from stock solutions in DMSO (1 mg/mL) by dilutions using PRMI cell medium supplemented with 10% FCS. Cells were then pre-incubated with increasing concentrations of antagonists for 60 min and then stimulated with 10 ng/mL *E. coli* O111:B4 LPS (a kind gift of Otto Holst, Research Center Borstel, Borstel, Germany) or, alternatively, cells were pre-incubated for 60 min with a high dose of antagonists (10 µg/mL) and stimulated with increasing concentrations of *E. coli* O111:B4 LPS. hTLR4 antagonist DA193 was used as positive control. After a culture period of 20 h at 37 °C, culture supernatants were harvested and the TNF-α and IL-1β contents were determined using an ELISA according to the manufacturers’ protocol (Thermo Fisher Scientific, Dreieich, Germany). Data shown are combined from n = 3 independent experiments, error bars indicate standard error of the mean.

**Inhibition of LPS-induced TNF-α release in a bone marrow-derived mouse wt macrophage cell line.**

Immortalized C57BL/6 wt mouse macrophage cell lines were kindly provided by D.T. Golenbock (Worcester, MA, USA) and propagated in RPMI medium (PAA, Pasching, Austria) containing 10% FCS, 20 mM HEPES buffer, 2 mM L-Glutamin (PAA, Austria) and 20 µg/mL gentamicin (Sigma-Aldrich GmbH, Taufkirchen, Germany). Cells were pre-treated with DLAMs antagonists or, DA193/DA256 either at 10 µg/mL or with increasing concentrations. After 60 min, the cells were stimulated with increasing concentrations or a fixed concentration of 10 ng/mL of *E. coli* O111:B4 LPS (a kind gift of Otto Holst, Research Center Borstel) for 20 h. TNF-α production was measured by mouse TNF-α CytoSet ELISA (Invitrogen) according to the manufacturer’s instruction. Data shown were combined from n = 2 independent experiments, error bars indicate standard error of the mean.

**Inhibition of LPS-induced cytokine release in TPA-primed THP-1 cells.**

The THP-1 cells (ATCC) were grown in cell-culture medium RPMI-1640 (Thermo Fisher Scientific, Life Technologies, WA, USA) supplemented with 100 U/mL penicillin, 2 mM L-glutamine, 100 µg/mL streptomycin, and 10% FCS. Cells were seeded in a 96-well plate at 10^5^ cells/well in 150 µL complete medium and stimulated by treatment with 200 nM TPA (12-*O*-tetradecanoylphorbol-13-acetate, Merck, Darmstadt, Germany) for 24 h to induce the differentiation into macrophage-like cells. On the next day, the cells were washed twice with complete culture medium to discard the cells that did not adhere, refreshed with 200 µL complete medium, and left for 1 h to recover. Solutions of synthetic antagonists were prepared starting from 1 mg/mL stock solutions in DMSO above using RPMI-1640 cell-culture medium supplemented with 10% FCS to reach the concentrations of 100 ng/mL and 1000 ng/mL. Solutions of antagonists were added to the cells as solutions in 10 µL complete medium at the indicated concentrations simultaneously with *E. coli* 0111:B4 LPS (InvivoGen, Toulouse, France). After stimulation, the final volume of the well reached 220 µL. The cells were incubated for 18 h and the supernatants were analyzed for IL-6 and TNF-α ELISA (BD Biosciences). Data are the mean of n = 3 samples and are representative of n = 3 independent experiments. Error bars indicate standard deviation.

**Inhibitory effects of synthetic TLR4 antagonists on LPS induced DC maturation.**

CD14^+^ monocytes were isolated using CD14 Microbeads (Miltenyi Biotec, Bergisch-Gladbach, Germany) from MNC fractions of buffy coat preparations of healthy individuals, according to the manufacturer’s protocol. Monocyte-derived dendritic cells (moDCs) were generated in cultures supplemented with 100 ng/mL GM-CSF and 35 ng/mL IL-4 for 6 days (obtained from Peprotech). Then, 1 × 10^5^ cells were plated in 500 µL of RPMI media supplemented with 1% Pen/Strep, 10% FBS, and 100 ng/mL GM-CSF and stimulated with 10 ng/mL LPS from *E. coli* 0111:B4 (InvivoGen, Toulouse, France), with or without TLR 4 antagonist (100, 500, or 1000 ng/mL) or using DMSO only. After incubation for 24 h, cells were processed for flow cytometric analysis. Cells positive for CD11b (obtained from BioLegend, Amsterdam, The Netherlands) and CD1a (obtained from BD, Heidelberg, Germany) were gated and analyzed for CD86 (obtained from BD Biosciences). Flow cytometry was performed using LSR Fortessa with Diva 8 Software (BD Bioscience); data analysis was performed using FlowJo v10 and GraphPad Prism V9 Software, error bars indicate standard error of the mean.

**Inhibition of LPS-induced cytokine production in human bronchial epithelial cell line Calu-3.**

Human lung epithelial cell line Calu-3 (ATCC) was seeded in 96-well plates at 10^5^ cells/well in 100 µL of complete medium (RPMI1640 (PAA), 1% PS (PAA), and 10% FCS (Biochrom, Berlin, Germany). On the next day, cells were washed once with complete medium and pre-treated with increasing concentrations of DLAM antagonists or DA193 as positive control. After 60 min, the cells were treated with *E. coli* 0111:B4 LPS (a kind gift of Otto Holst, Research Center Borstel, Borstel, Germany), the cells were incubated for 20 h, and the supernatants were analyzed for cytokines (IL-8 and IL-6) by ELISA (Thermo Fisher Scientific, Dreieich, Germany). Data shown are combined from n = 3 (IL-6)/n = 2 (IL-8) independent experiments, error bars indicate standard error of the mean.

## 4. Conclusions

With the aim of investigating species-specific ligand–protein interactions, we developed synthetic glycophospholipids which mimic the primary and protein-bound tertiary structure of the endotoxic portion of LPS, lipid A. Four differently phosphorylated glycophospholipids, *N,N′*-acylated by the long-chain acyloxyacyl residues, were prepared via a divergent synthetic route and assayed for TLR4/MD-2-specific inhibitory activity in human and mouse cell lines. The affinity of the hyper-phosphorylated compounds for MD-2 was generally reduced, whereas the bis-phosphorylated **DLAM33** showed nanomolar inhibitory activity in the LPS-challenged human cell lines. The ability of **DLAM33** to effectively suppress the release of pro-inflammatory cytokines was confirmed in two different experimental settings. In human immune cells (MNC, DCs) and epithelial cells (Calu-3) stimulated with 10 ng/mL *E. coli* LPS, **DLAM33** was able to block pro-inflammatory signaling to background levels at a concentration of 100–500 ng/mL (in a cytokine-specific manner). Challenging mononuclear cells pre-treated with **DLAM33** with increasing concentrations of LPS (up to 1000 ng/mL) also confirmed its potent antagonist capacity and ability to compete with LPS for the binding site on MD-2, as cytokine production was completely blocked at an LPS concentration of 100 ng/mL (10-fold above the concentration required for receptor saturation) and partially blocked (to one-third of the original level) upon stimulation with 1000 ng/mL LPS.

In contrast to previously developed DLAMs derived from the βGlcN(1↔1)αGlcN backbone, which could not activate murine TLR4, **DLAM33** showed partial agonist activity in mTLR4-transfected HEK293 cells and murine macrophages. The LPS-induced cytokine release was suppressed at nano- to milli-molar concentrations of **DLAM33** (500–10,000 ng/mL), while the glycolipid itself induced cytokine production at levels that were a quarter of those induced by LPS, even at high concentrations.

These findings provide a deeper understanding of ligand–protein interactions in terms of species-specific ligand recognition by the TLR4 complex and revealthe structural requirements for partial TLR4 agonism. Given the evidence for a detrimental effect of the complete suppression of cytokine release in sepsis [20,56,57], compounds that are able to inhibit LPS-induced signaling while maintaining a modest activation of TLR4-mediated responses represent a valuable alternative.

## Data Availability

The data are available on request from the corresponding author.

## Supplementary Materials

The following supporting information can be downloaded at: https://www.mdpi.com/article/10.3390/molecules28165948/s1, NMR spectra of all synthetic compounds.
