# Supplementary material for: Exploring Species-Specificity in TLR4/MD-2 Inhibition with Amphiphilic Lipid A Mimicking Glycolipids
Source: Molecules. 2023 Aug 8;28(16):5948. doi: 10.3390/molecules28165948 (PMC10459247; doi:10.3390/molecules28165948)

Supporting Information

Exploring species-specificity in TLR4/MD-2 inhibition with amphiphilic lipid A mimicking glycolipids

Alessio Borio [1], Aurora Holdago [2], Christina Passegger [3], Herbert Strobl [3], Rudi Beyaert [2], Holger Heine [4] and Alla Zamyatina [1*]

NMR spectra of synthetic compounds and intermediates

2: ^{1}H-NMR, 600 MHz, $CDCl_3$

The presence of conformationally restricted rotamers in solution ($CDCl_3$) was confirmed for **1** and **2**; additional signals in the ^{1}H-NMR spectrum are not impurities but correspond to rotamers around the α,β-1,1 linkage of **2**. As soon as the steric constraint holding the two GlcN rings in the $^{4}C_1$ conformation is removed (by reductive opening of the 4,6-*O*-benzilydene acetal, as in **3**), the phenomenon disappears.

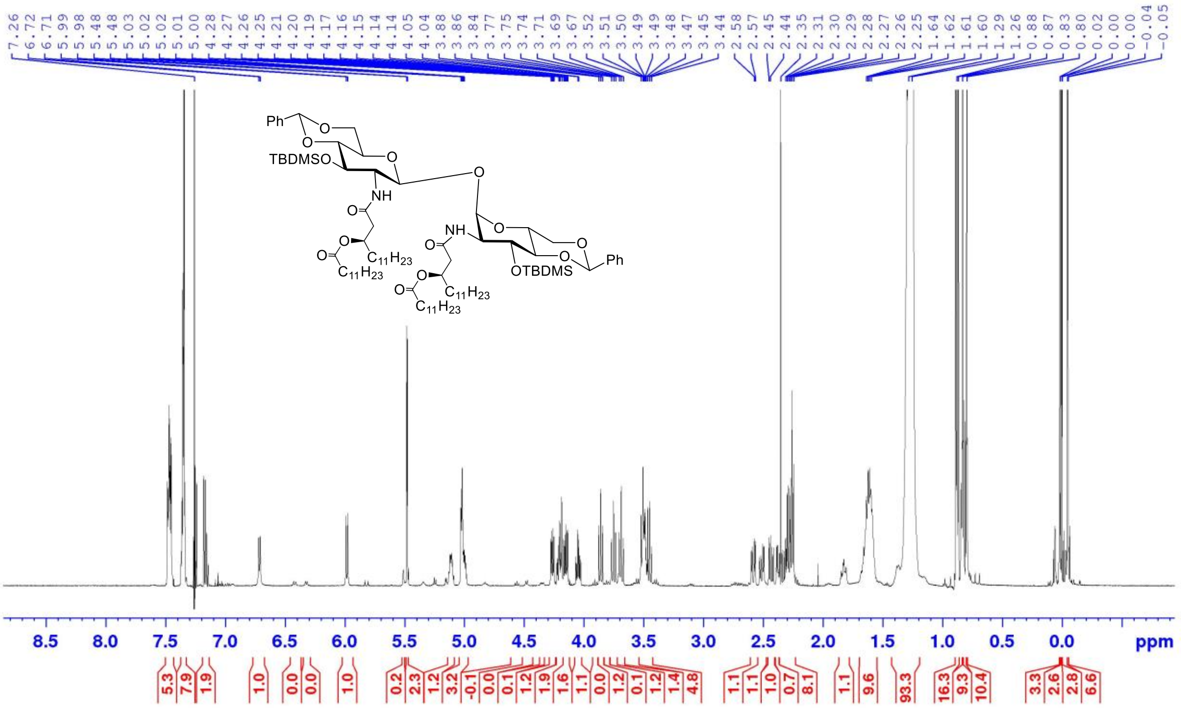

2: ^{13}C-NMR (APT), 150.9 MHz, $CDCl_3$

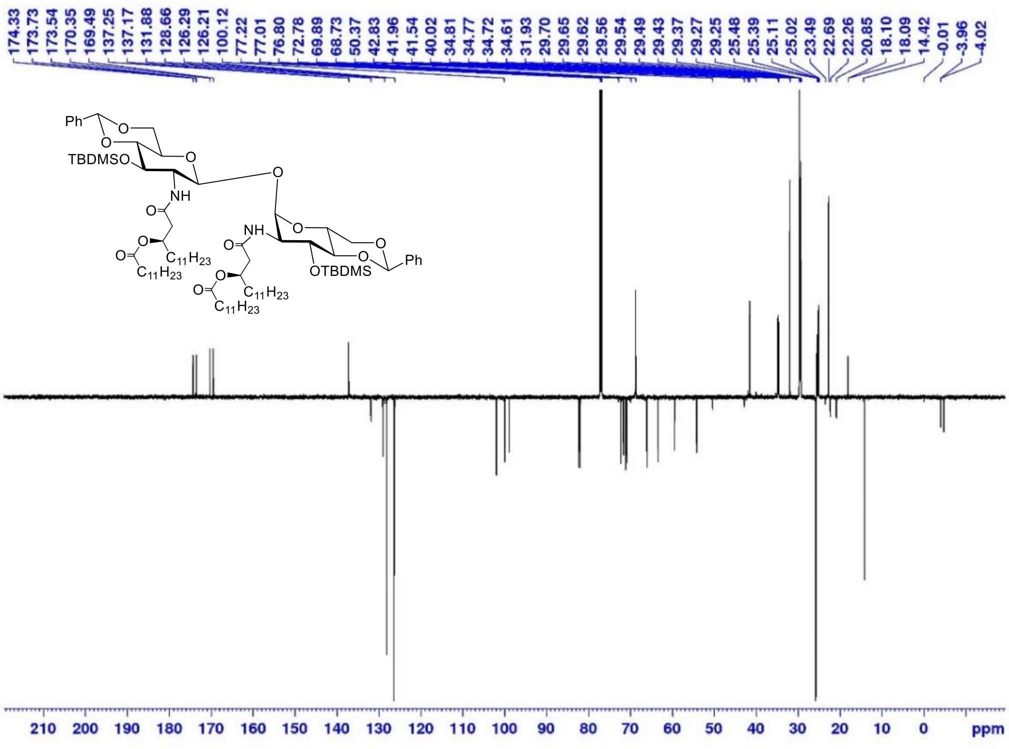

3: ^{1}H-NMR, 600 MHz, $CDCl_3$

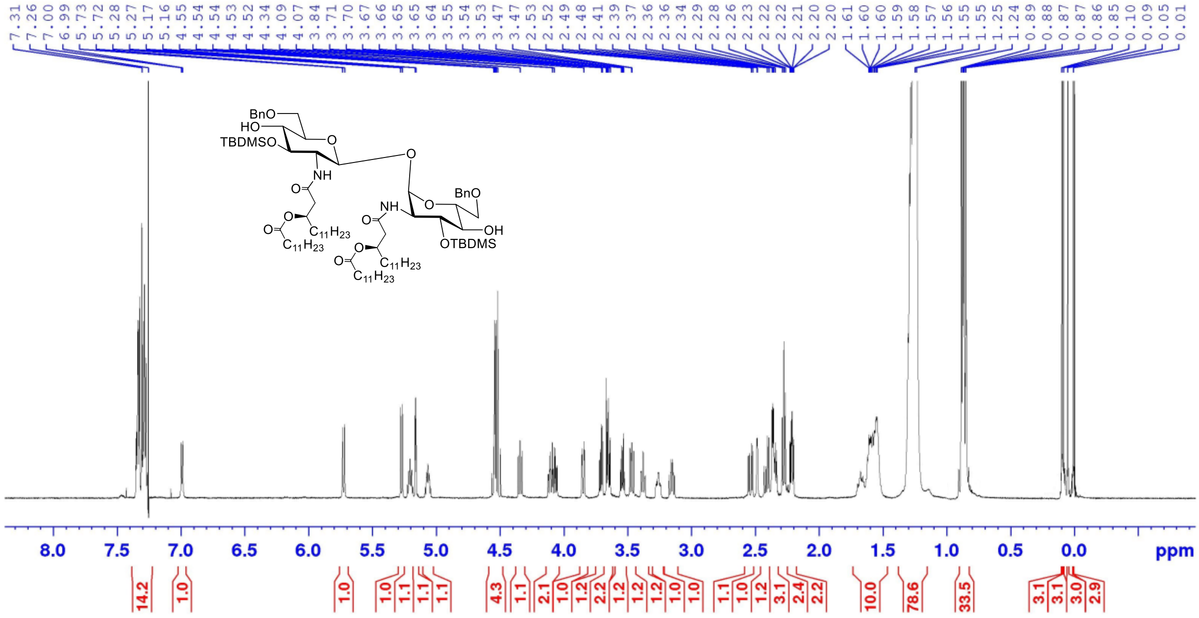

3: ^{13}C-NMR (APT), 150.9 MHz, $CDCl_3$

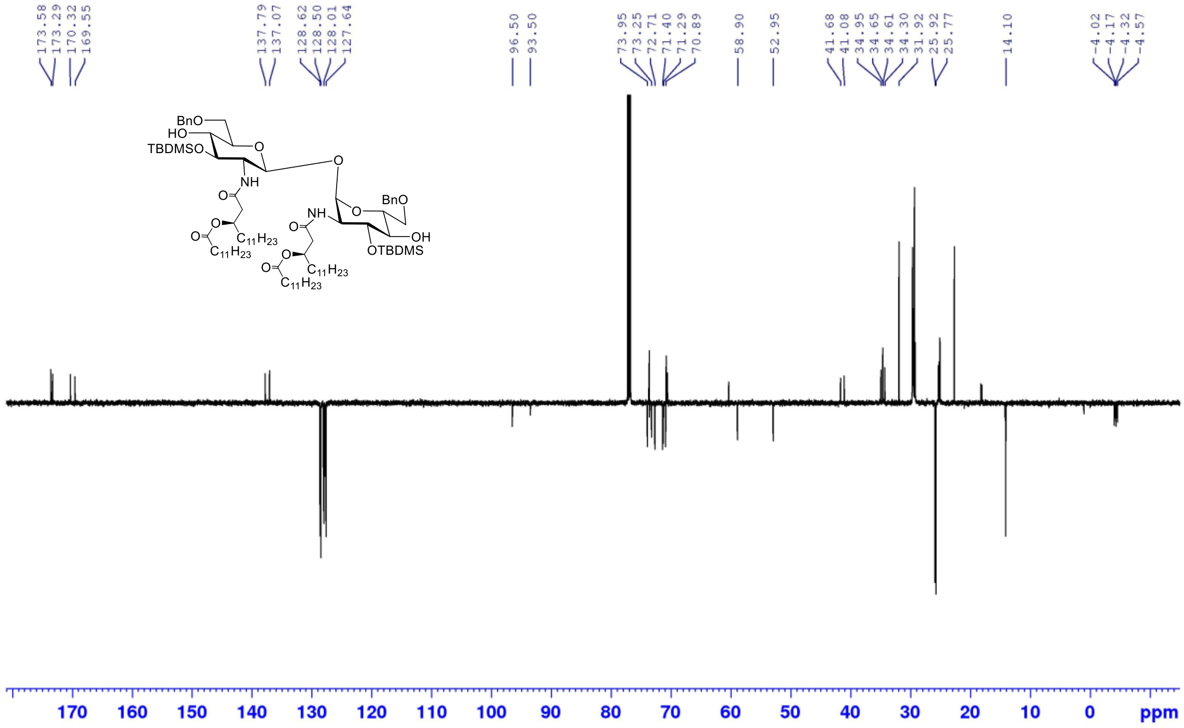

4: ^{1}H-NMR, 600 MHz, $CDCl_3$

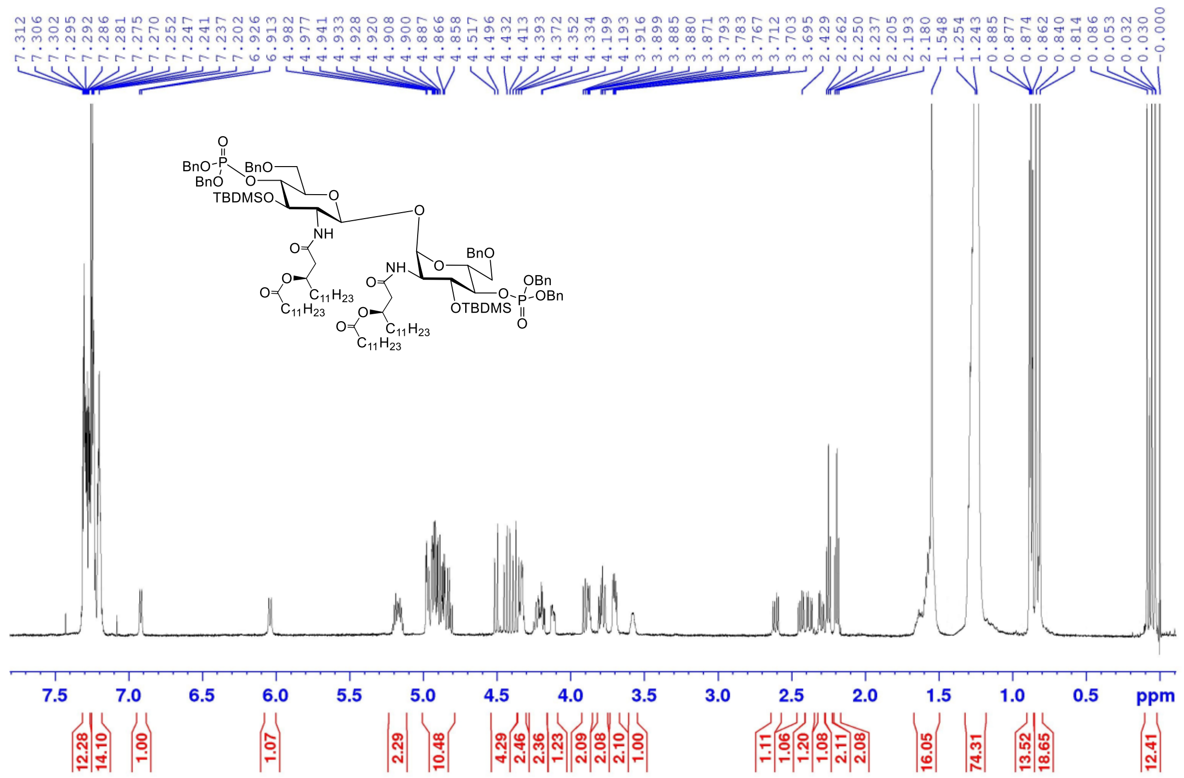

4: ^{13}C-NMR (APT), 150.9 MHz, $CDCl_3$

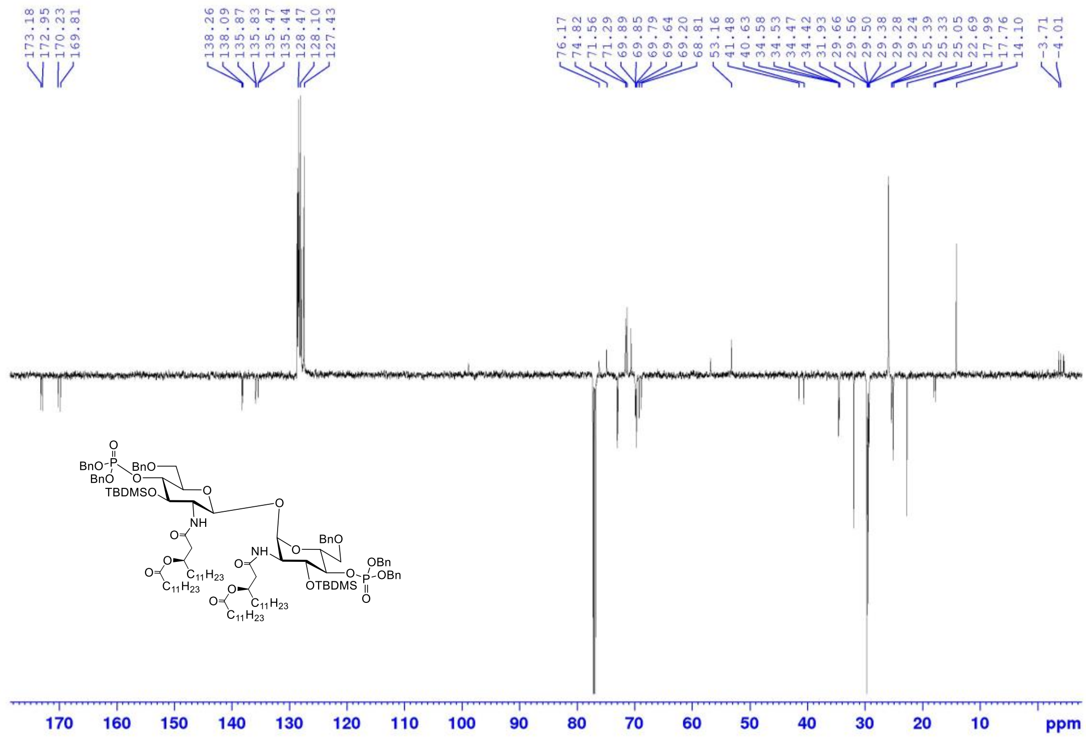

4: ^{13}C-NMR (DEPT-45), 150.9 MHz, $CDCl_3$

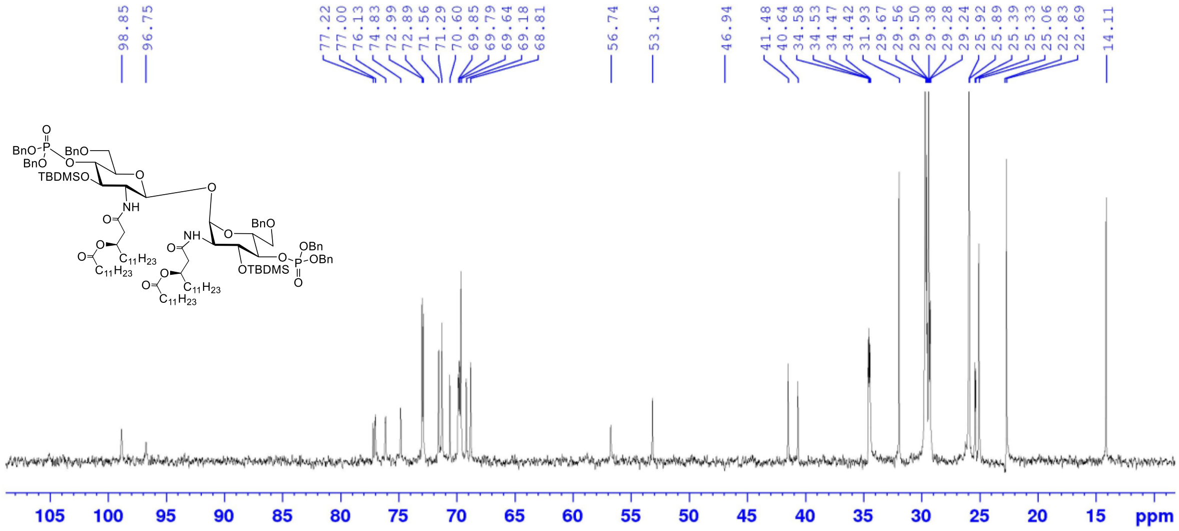

4: ^{31}P-^{1}H HMBC NMR, $CDCl_3$

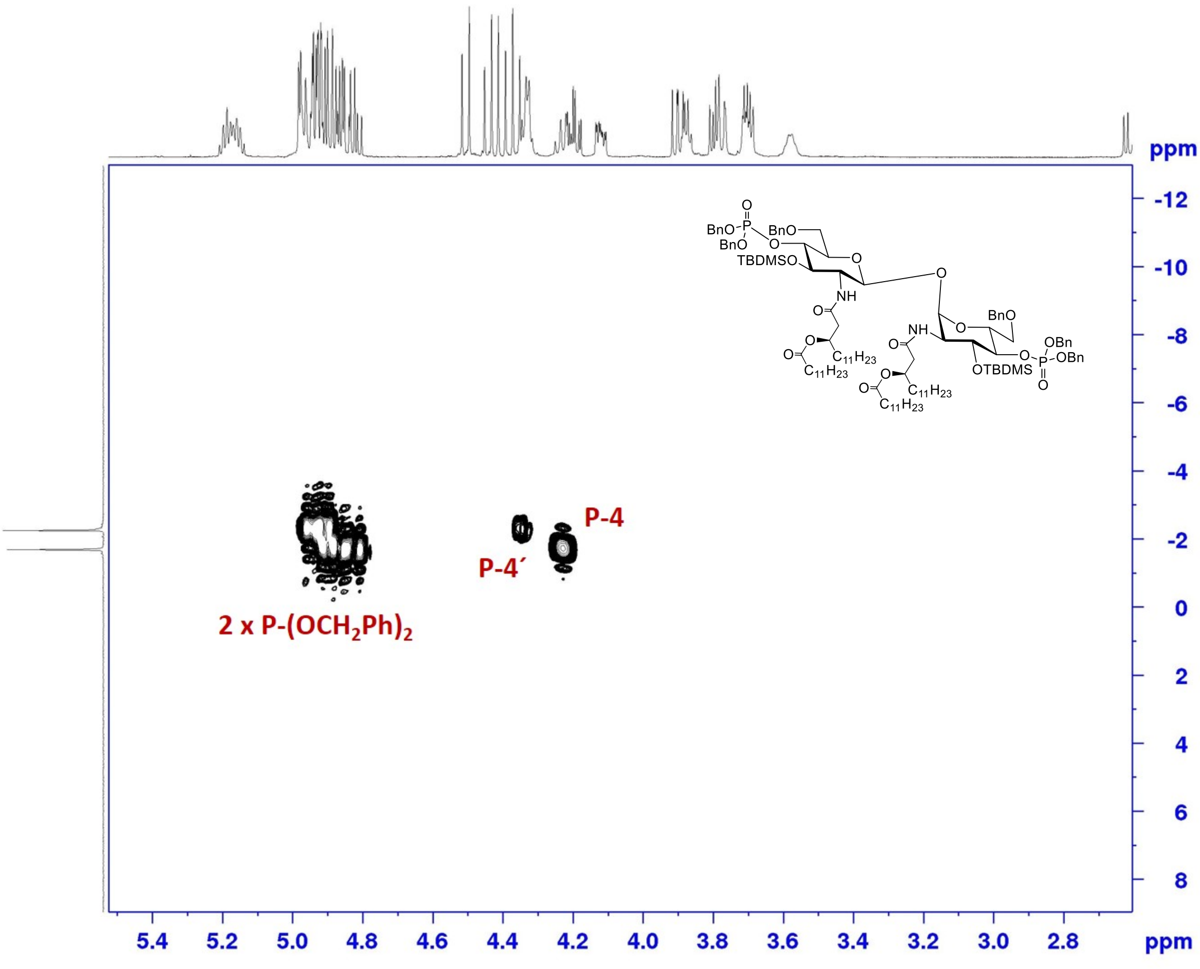

5: ^{1}H-NMR, 600 MHz, $CDCl_3$

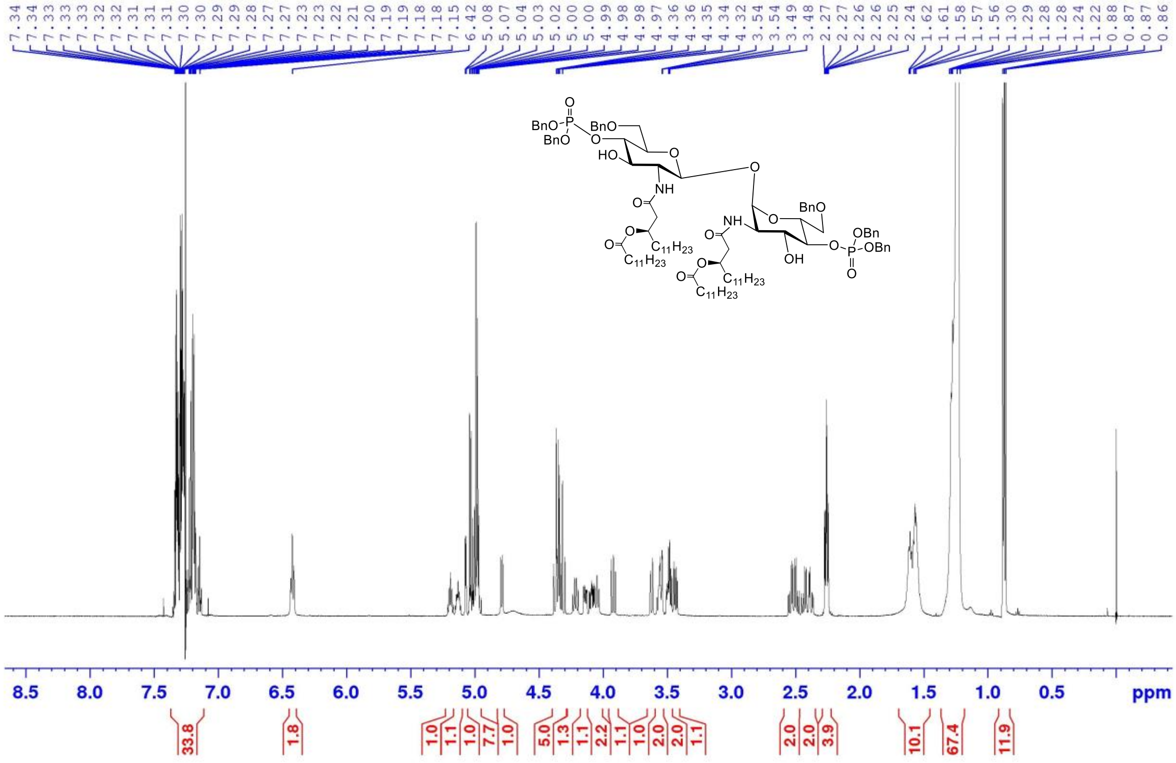

5: ^{13}C-NMR (APT), 150.9 MHz, $CDCl_3$

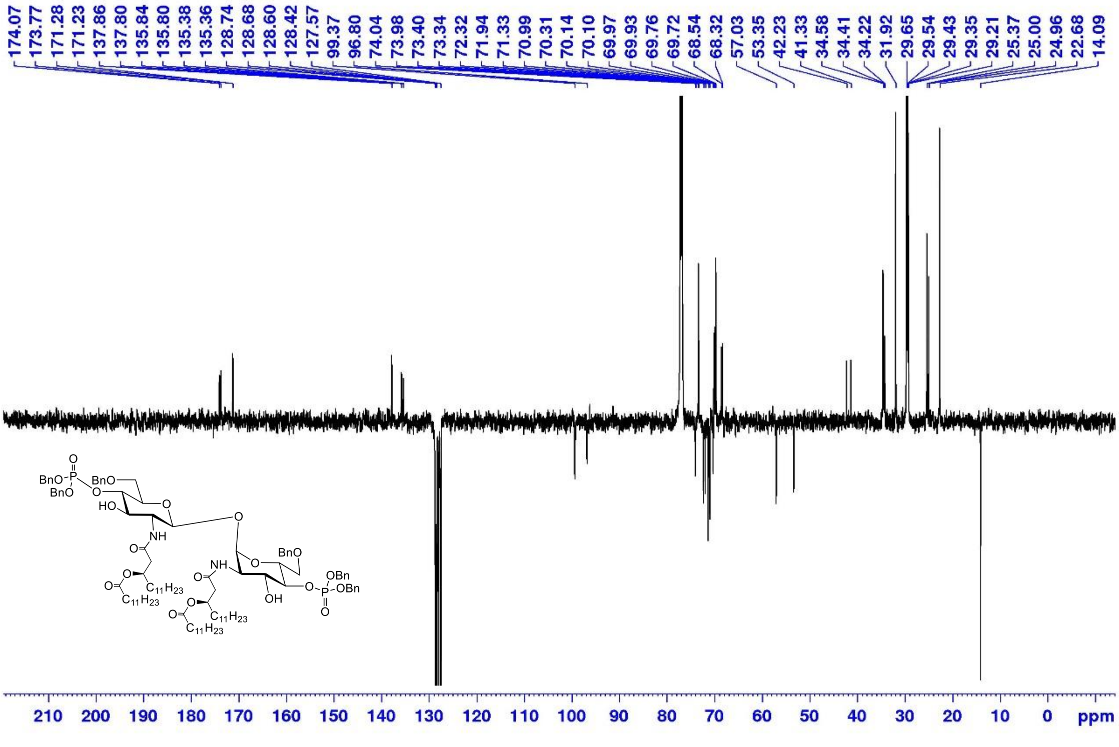

5: ^{31}P-NMR, 243 MHz, $CDCl_3$

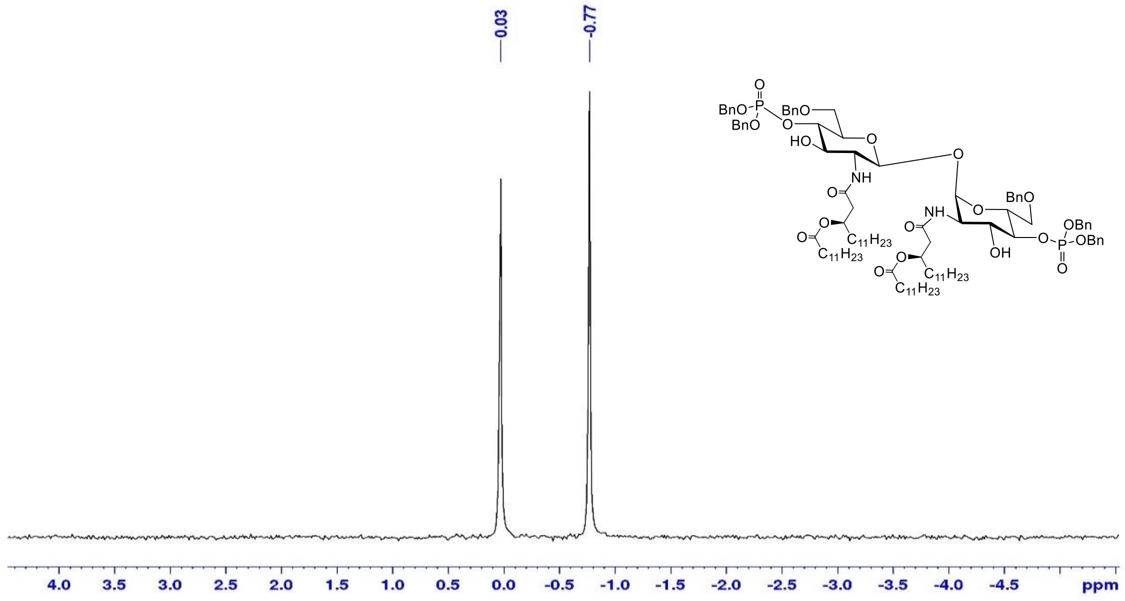

5: ^{1}H-^{31}P HMBC NMR, $CDCl_3$

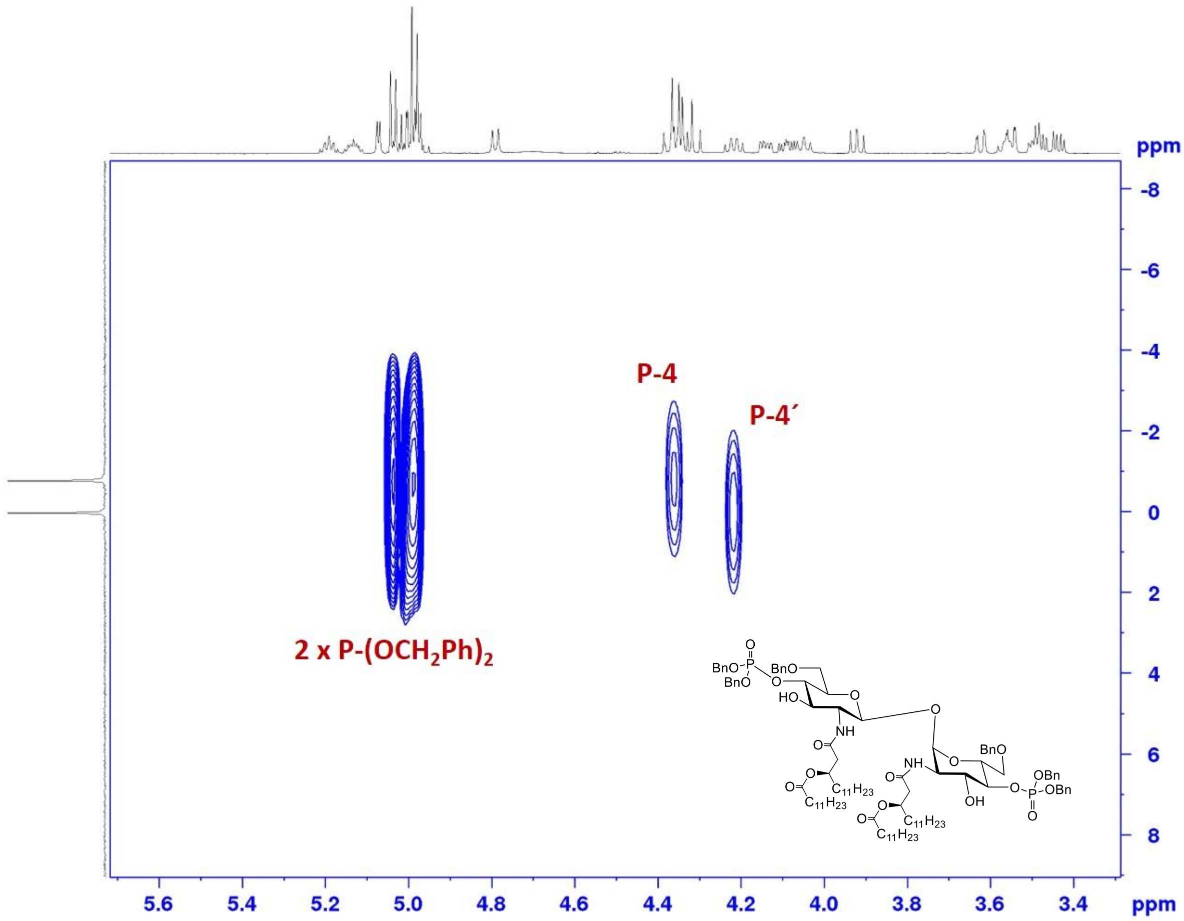

6: ^{1}H-NMR, 600 MHz, CDCl$_3$

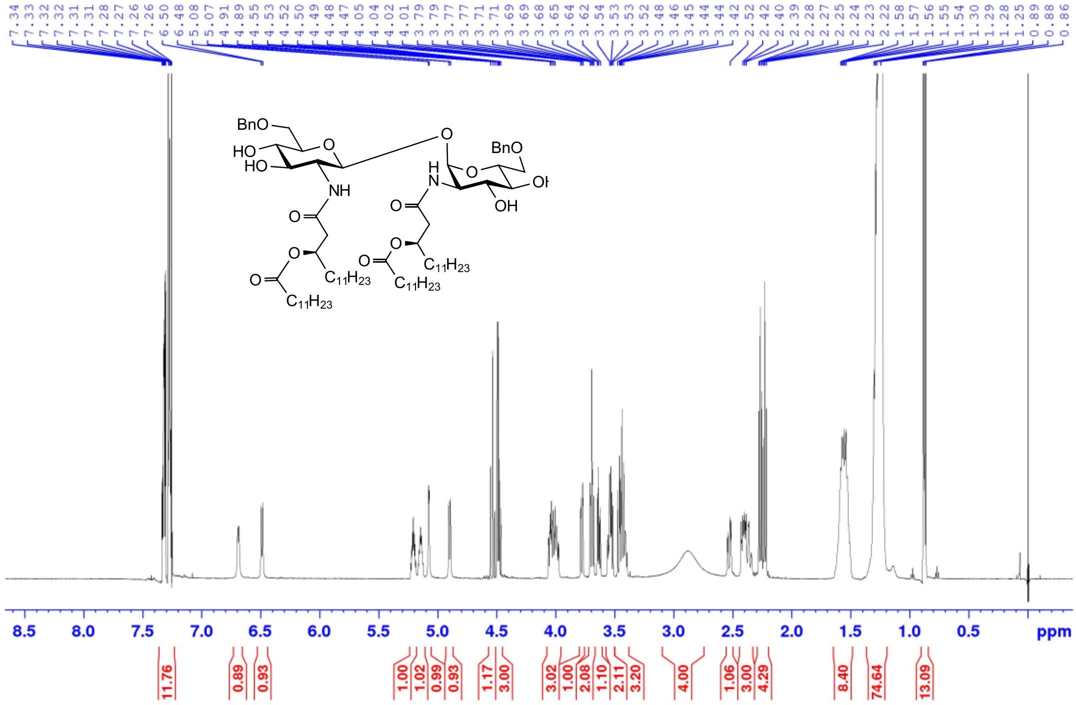

6: ^{13}C-NMR (APT), 150.9 MHz, CDCl$_3$

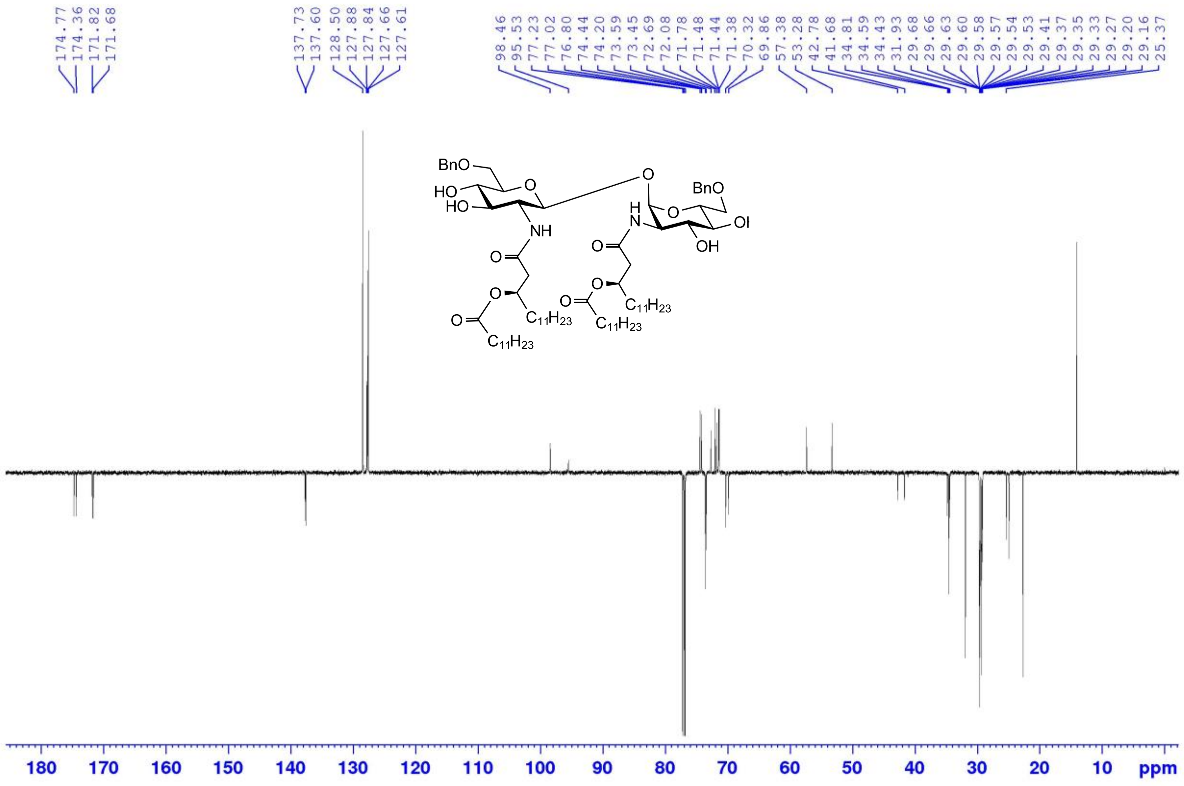

7: ^{1}H-NMR, 600 MHz, $CDCl_3$

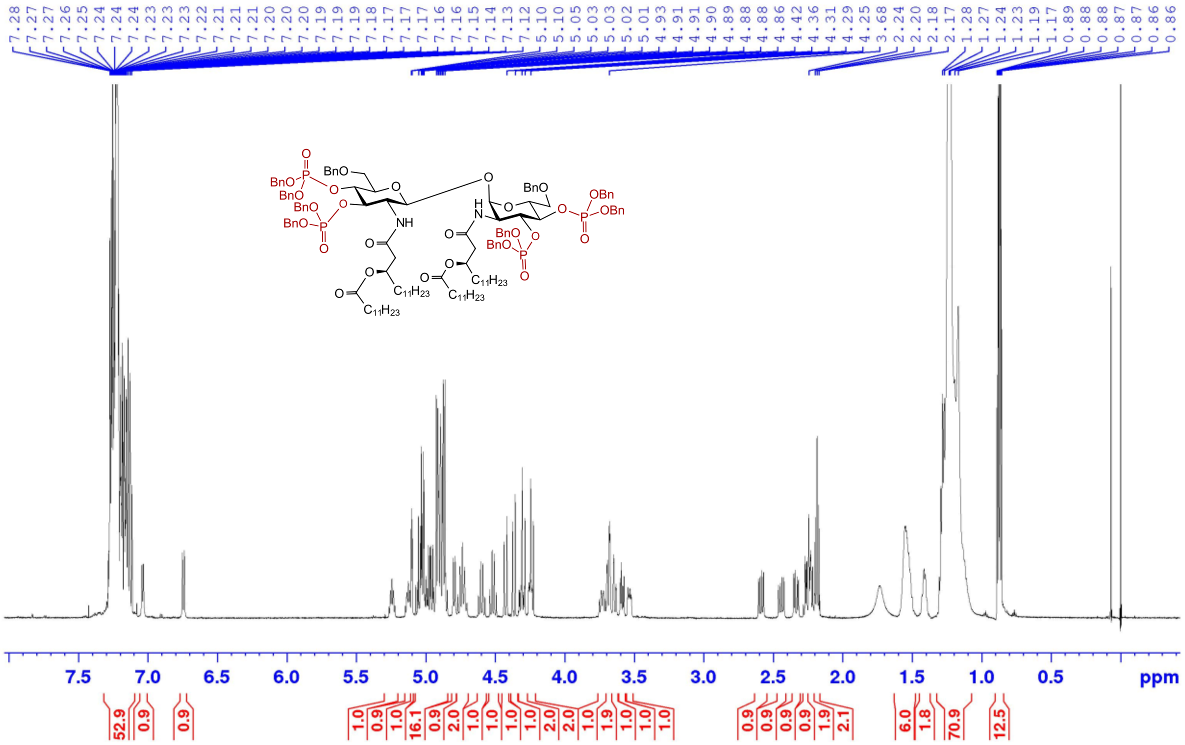

7: ^{1}H-NMR, 600 MHz, $CDCl_3$ (extension 5.5 – 3.0 ppm)

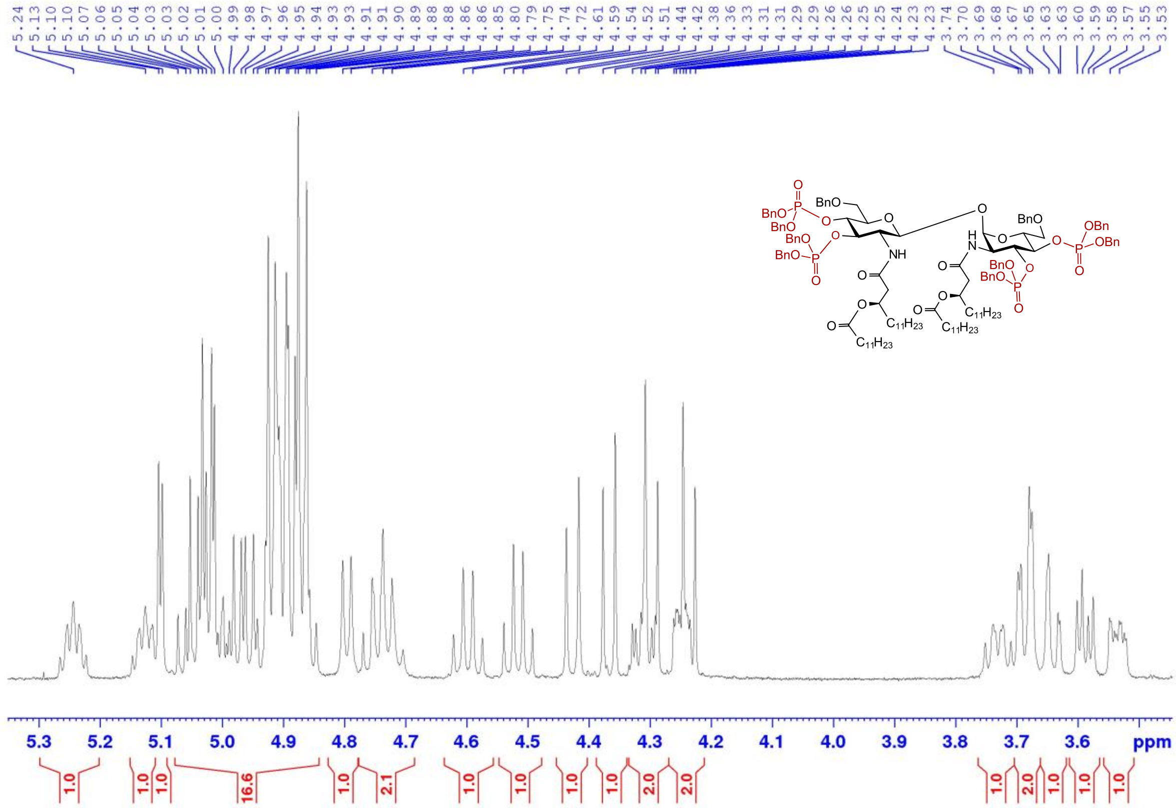

7: ^{1}H-^{31}P HMBC NMR, $CDCl_3$

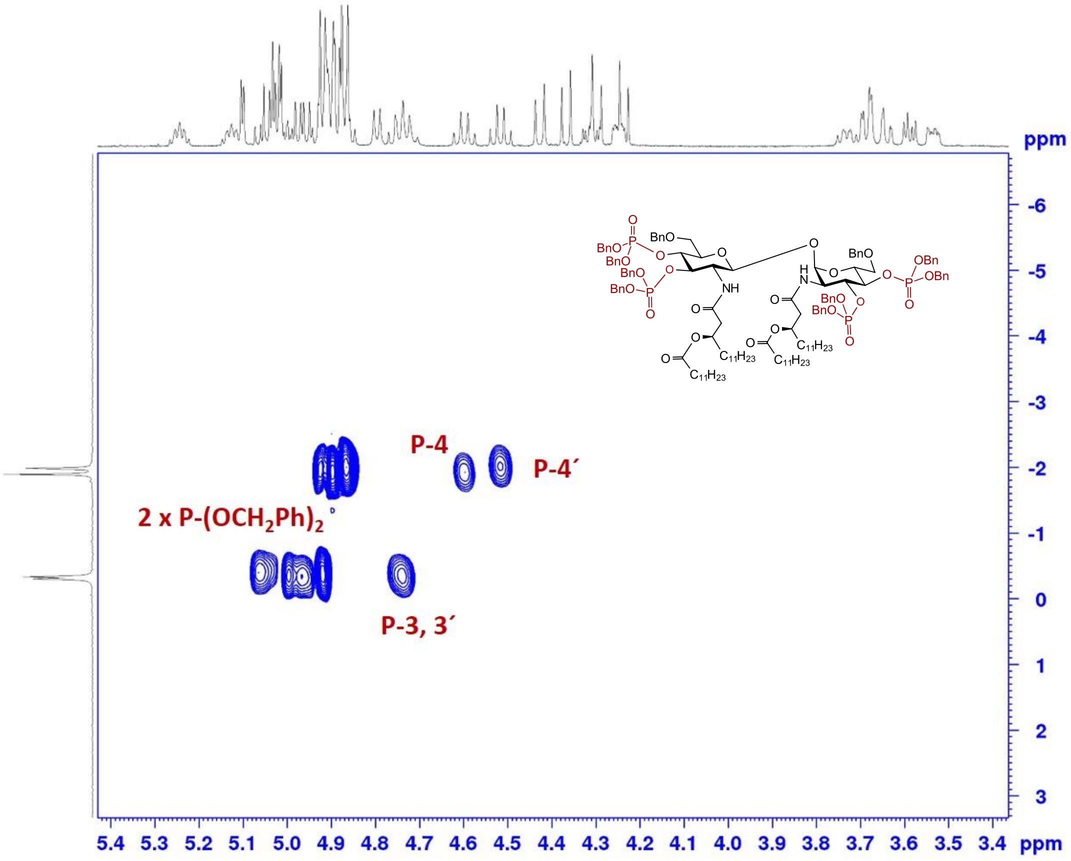

7: ^{1}H-^{13}C HSQC NMR, $CDCl_3$

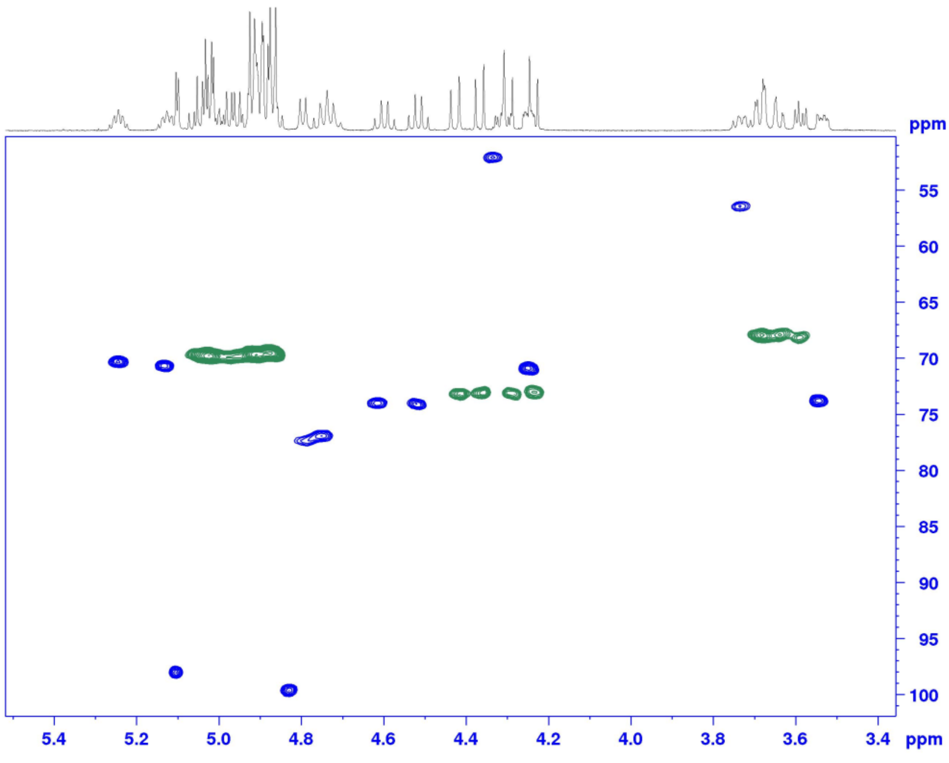

8 ^{1}H-NMR, 600 MHz, CDCl₃

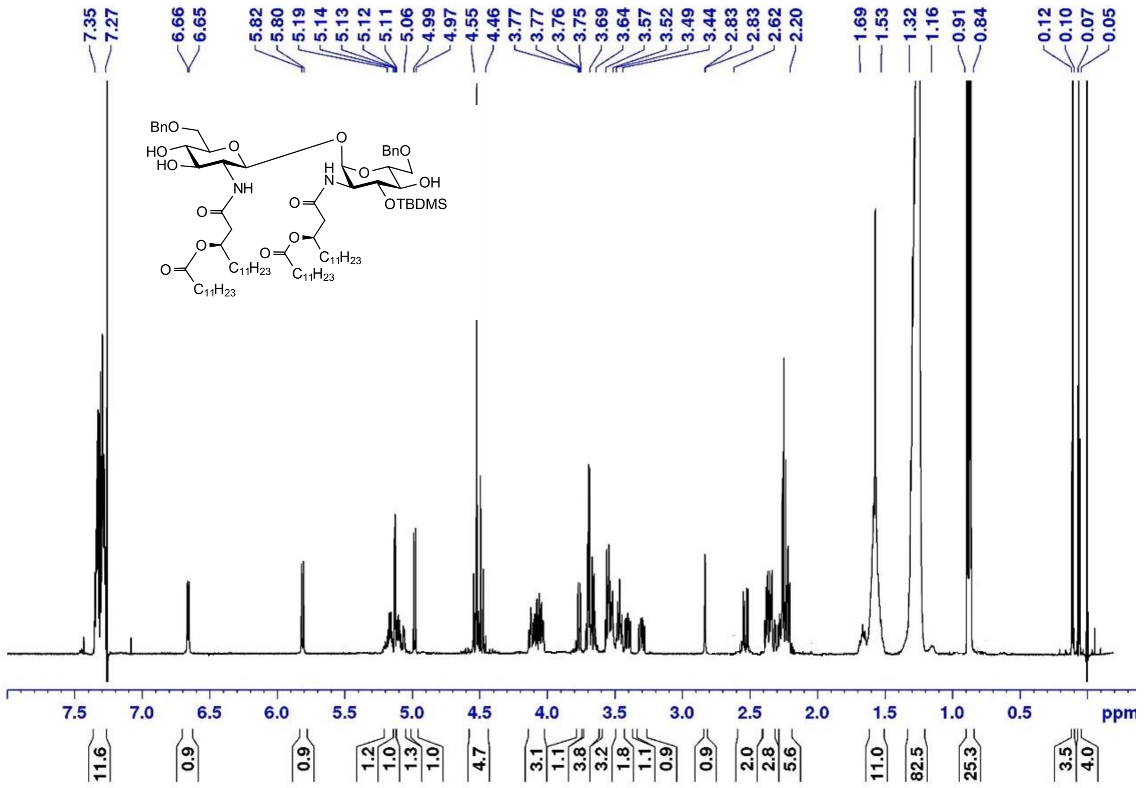

8: ^{13}C-NMR (APT), 150.9 MHz, CDCl$_3$

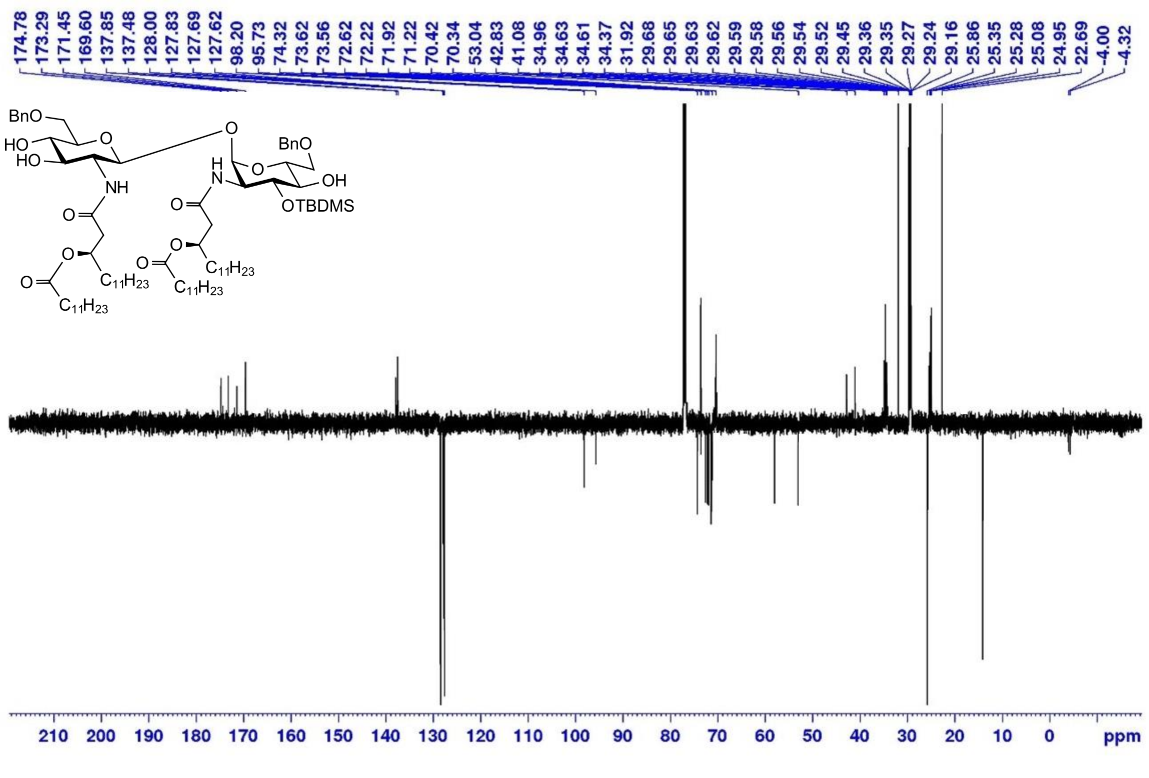

9: ^{1}H-NMR, 600 MHz, $CDCl_3$

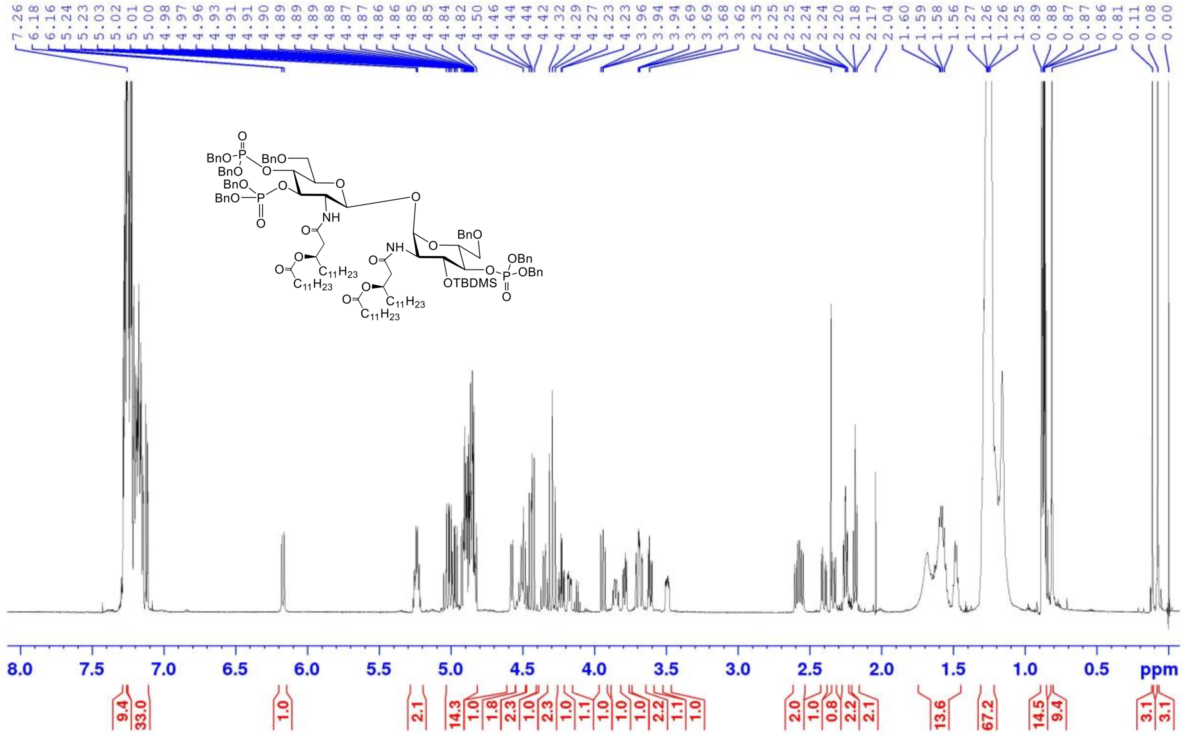

9: ^{13}C-NMR (APT), 150.9 MHz, $CDCl_3$

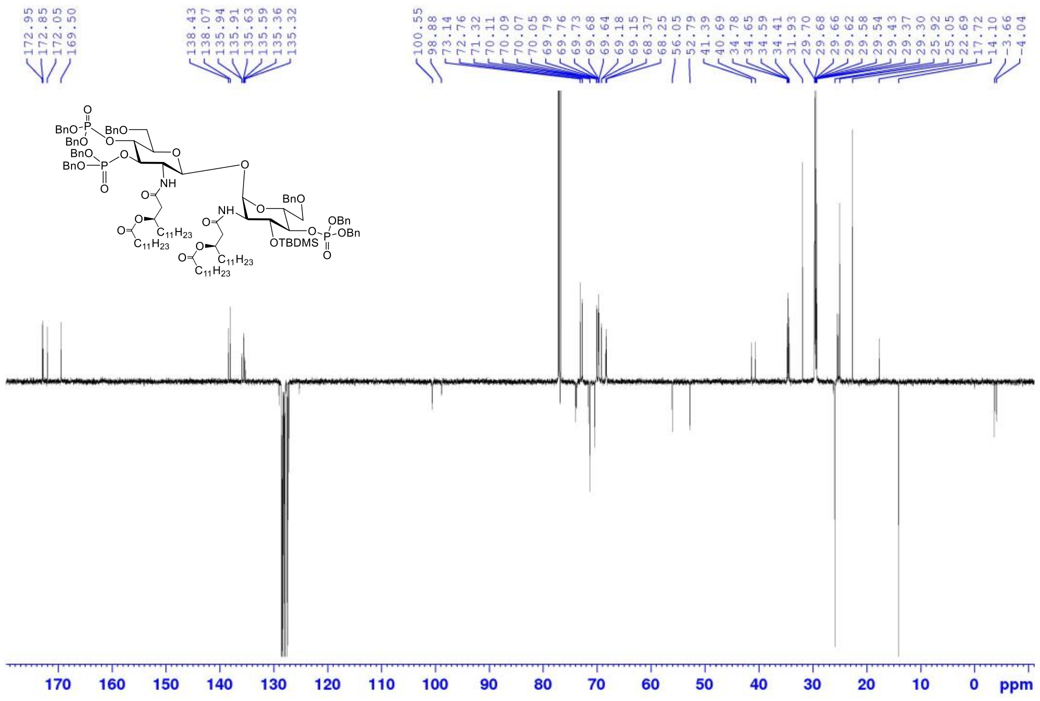

9: ^{1}H- ^{13}C HMBC-NMR, $CDCl_3$

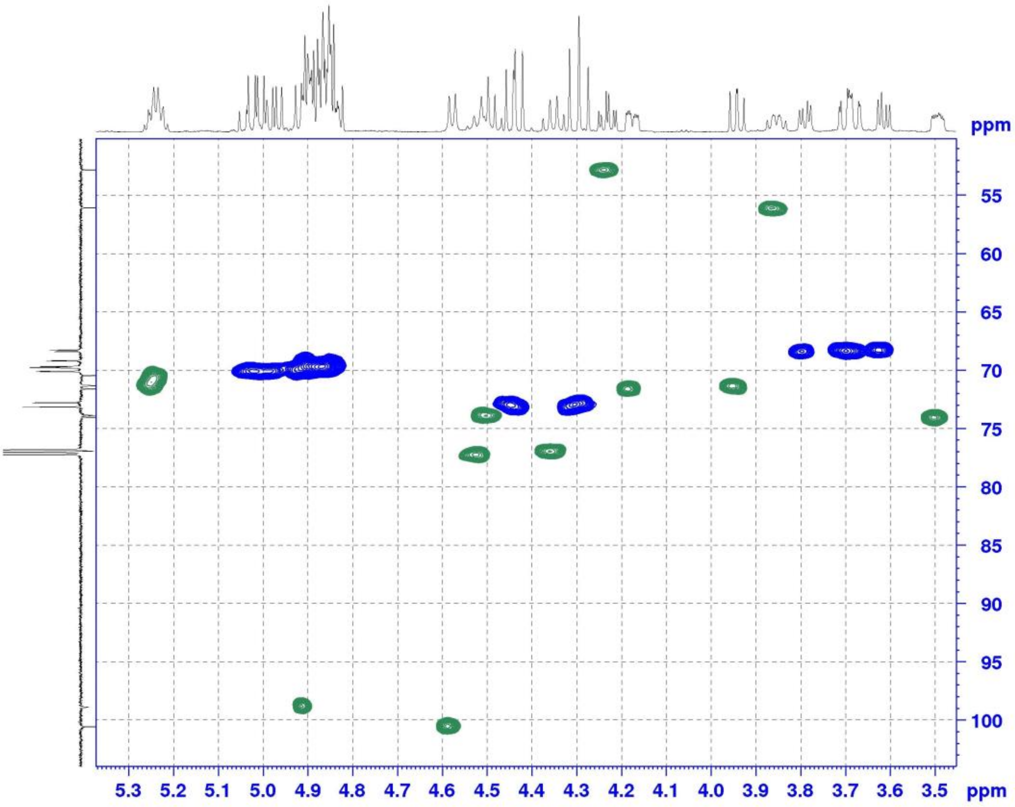

9: ^{1}H-^{31}P HMBC-NMR, $CDCl_3$

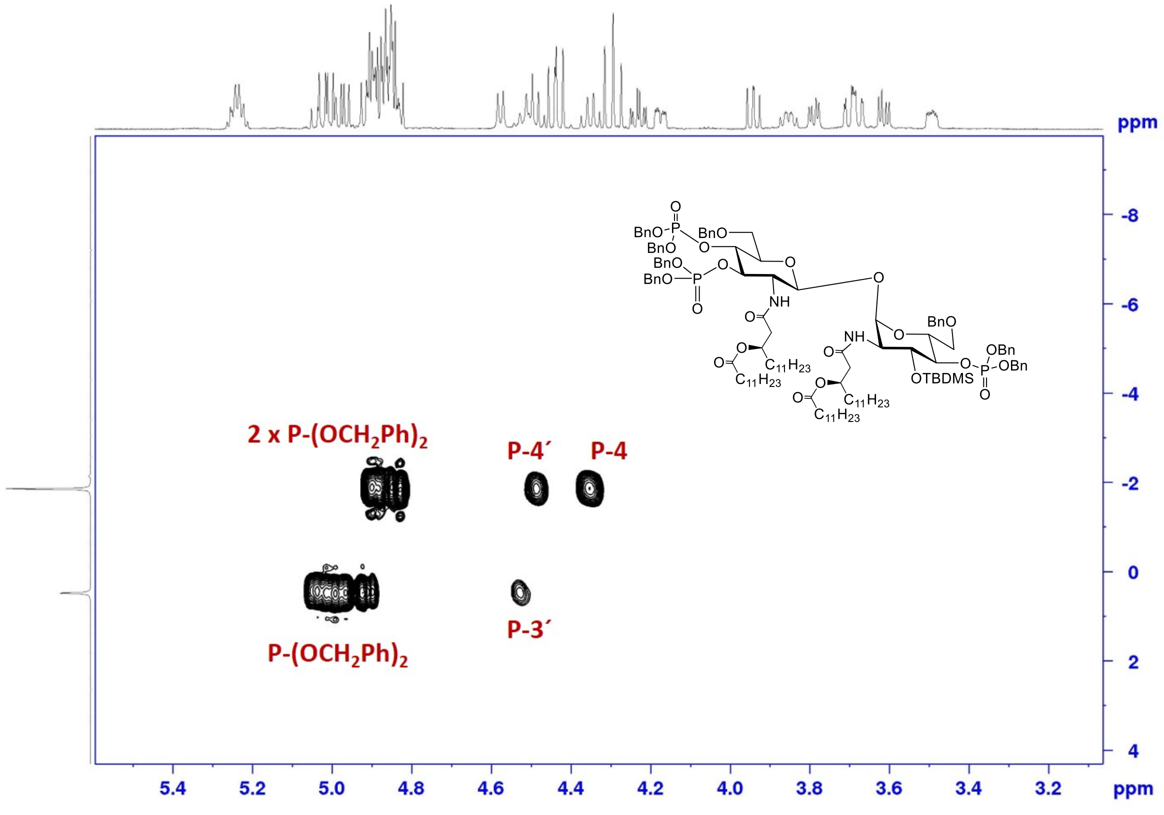

10: ^{1}H-NMR, 600 MHz, $CDCl_3$

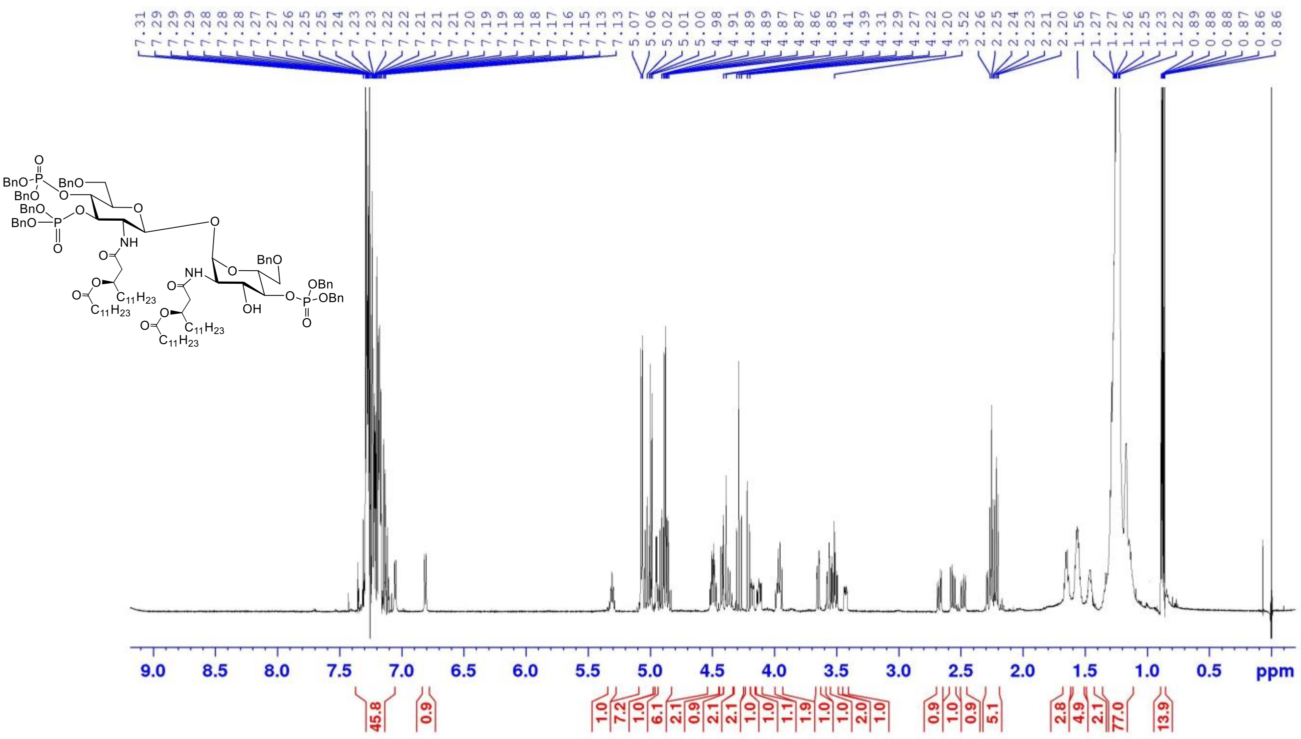

10: ^{13}C-NMR (APT), 150.9 MHz, $CDCl_3$

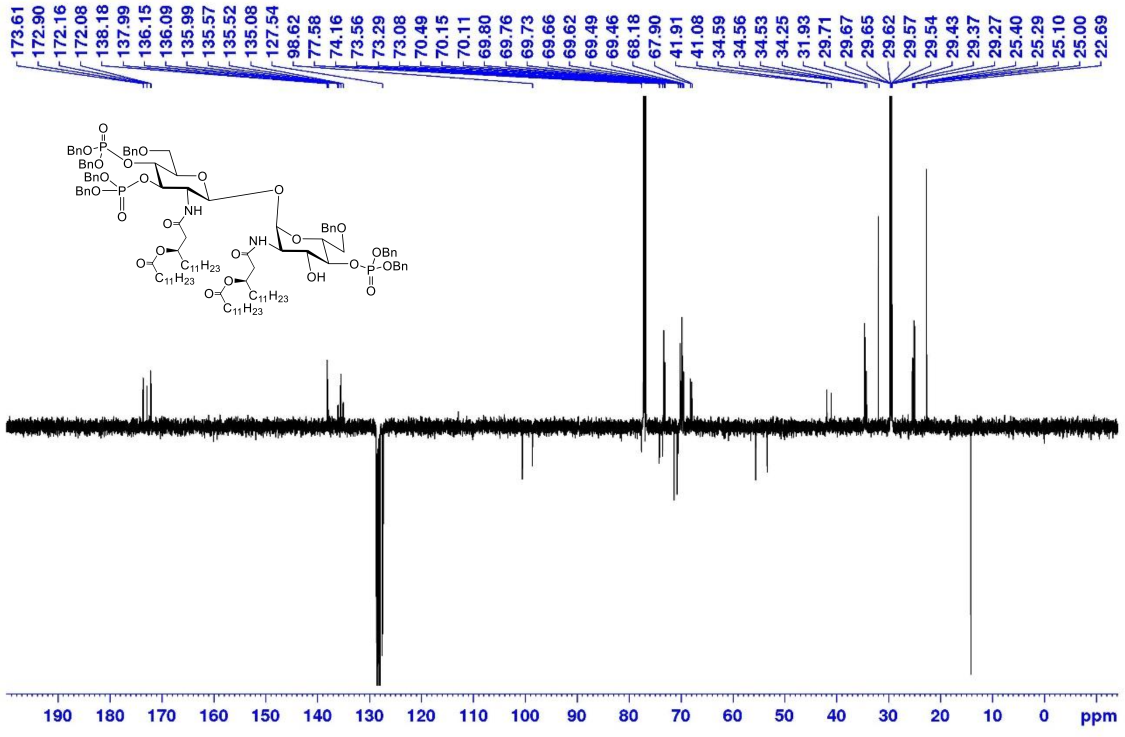

10: ^{1}H-^{31}P HMBC NMR, CDCl$_3$

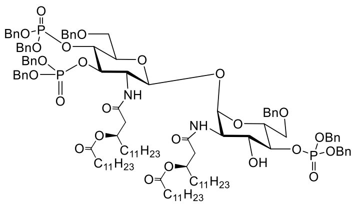

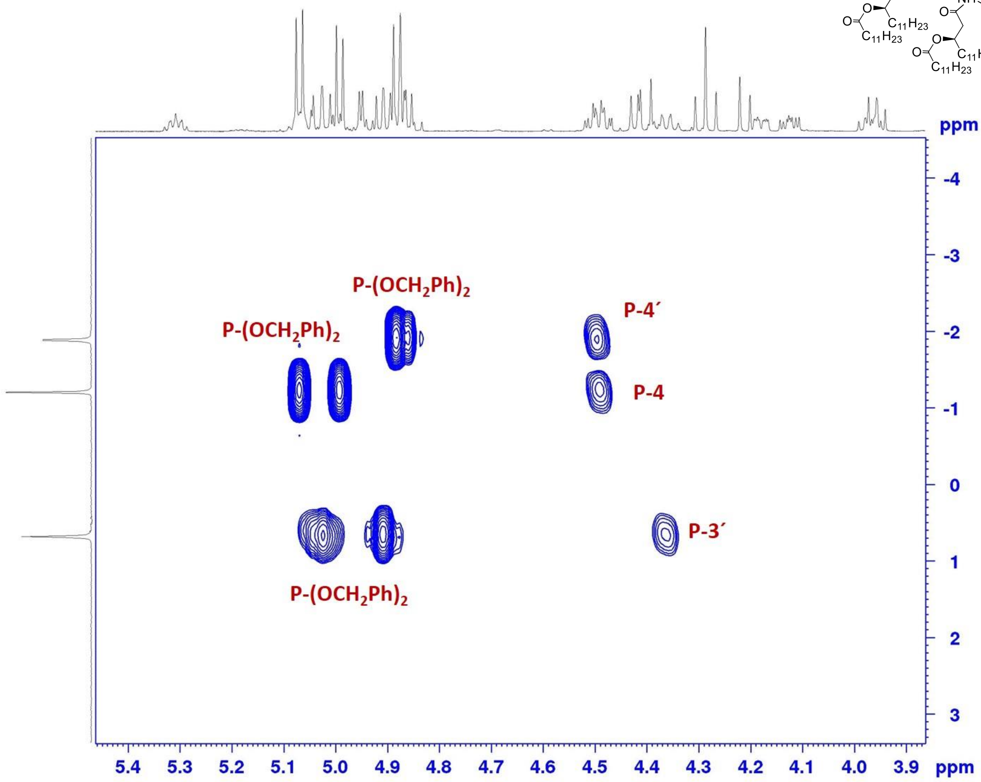

10: ^{1}H-^{13}C HMBC NMR, CDCl$_3$

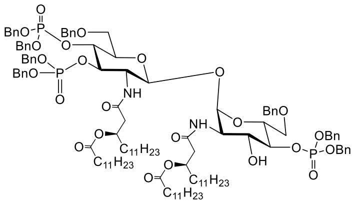

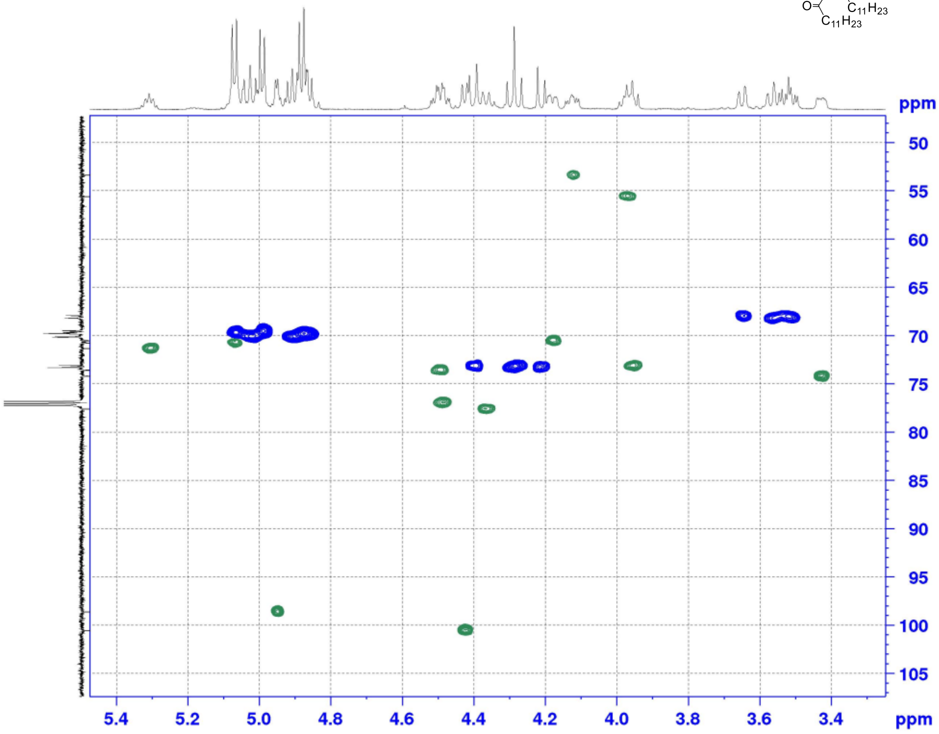

11: ^{1}H-NMR, 600 MHz, $CDCl_3$

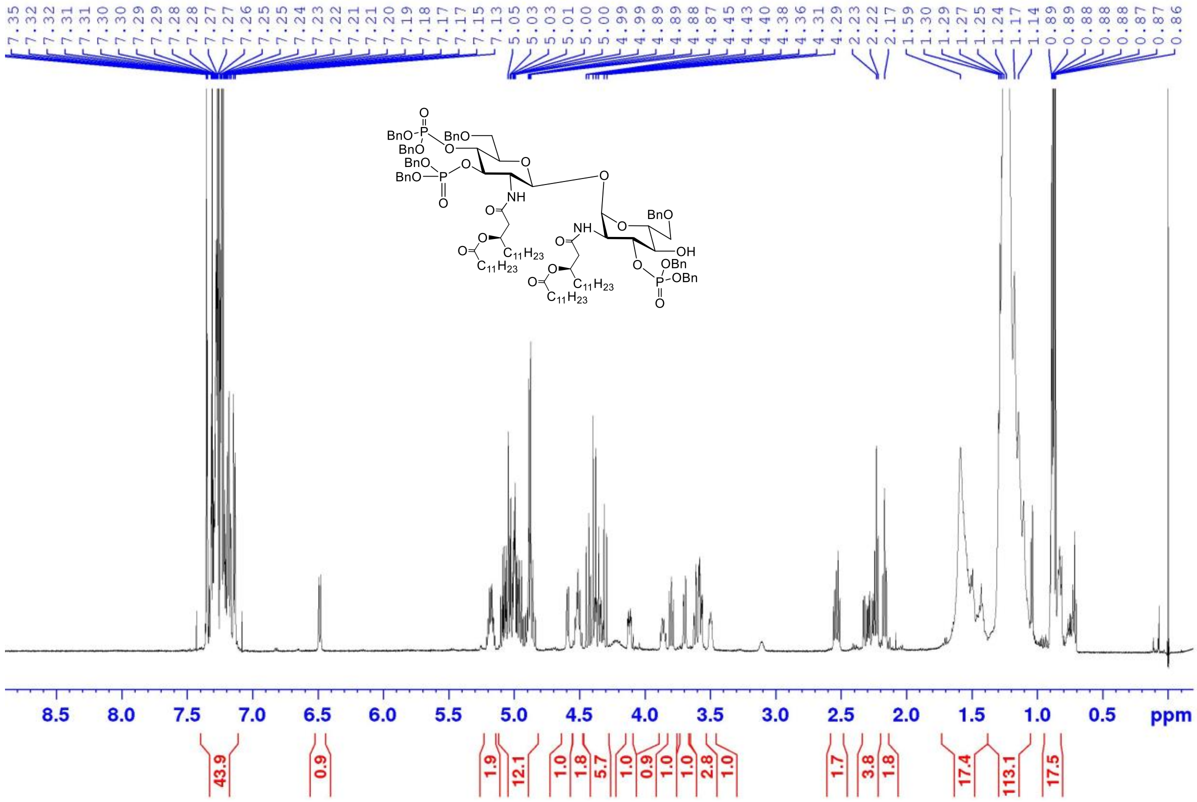

11: ^{13}C-NMR (APT), 150.9 MHz, $CDCl_3$

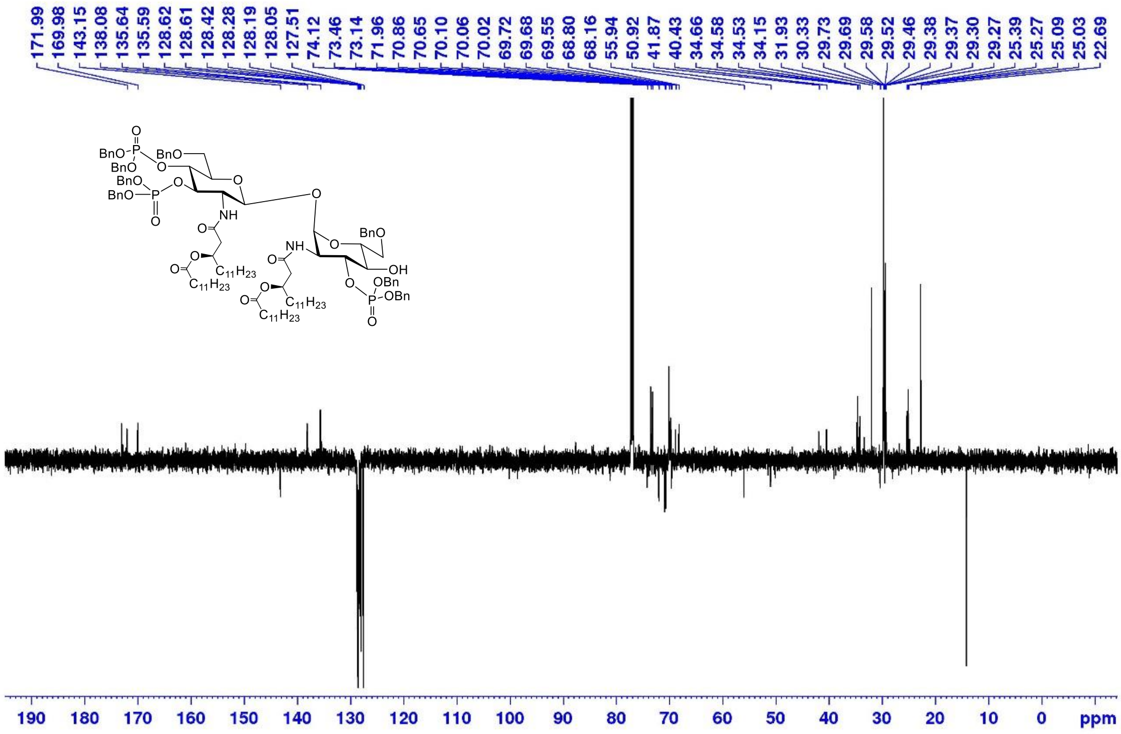

11: ^{1}H-^{31}P HMBC-NMR, $CDCl_3$

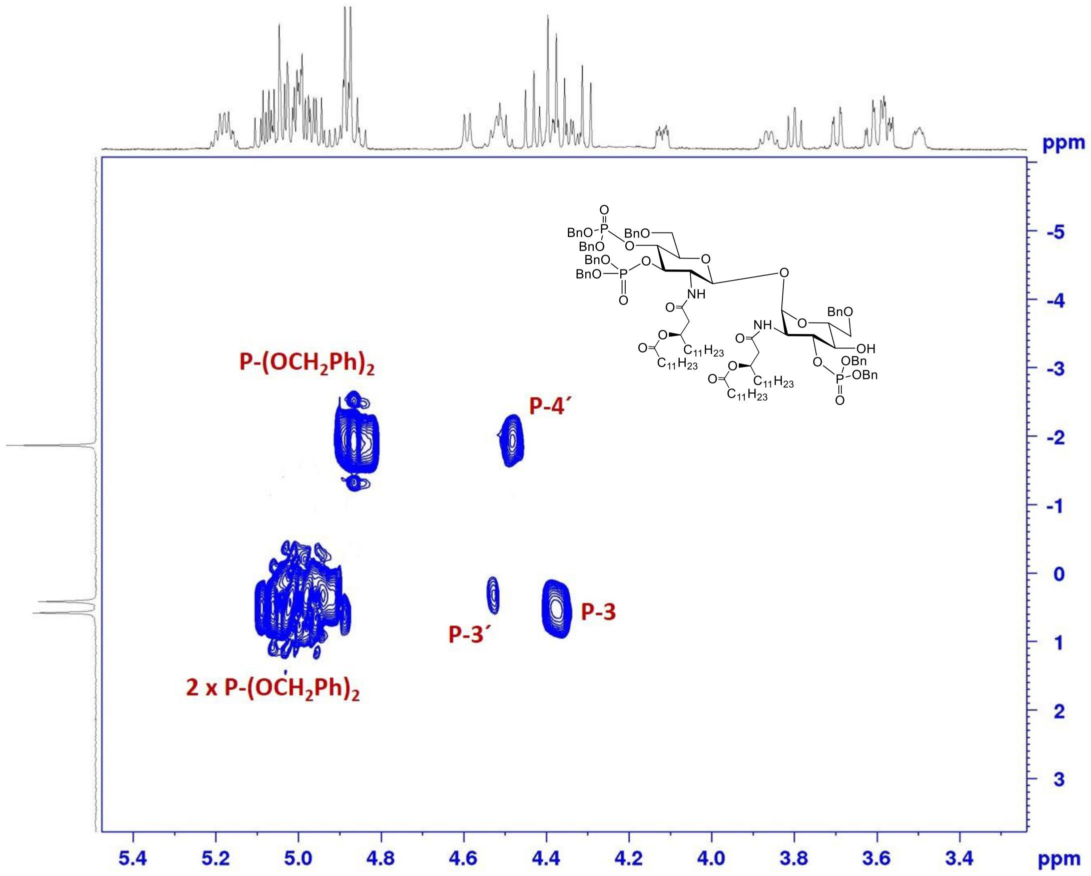

11: ^{1}H-^{13}C HMBC-NMR, $CDCl_3$

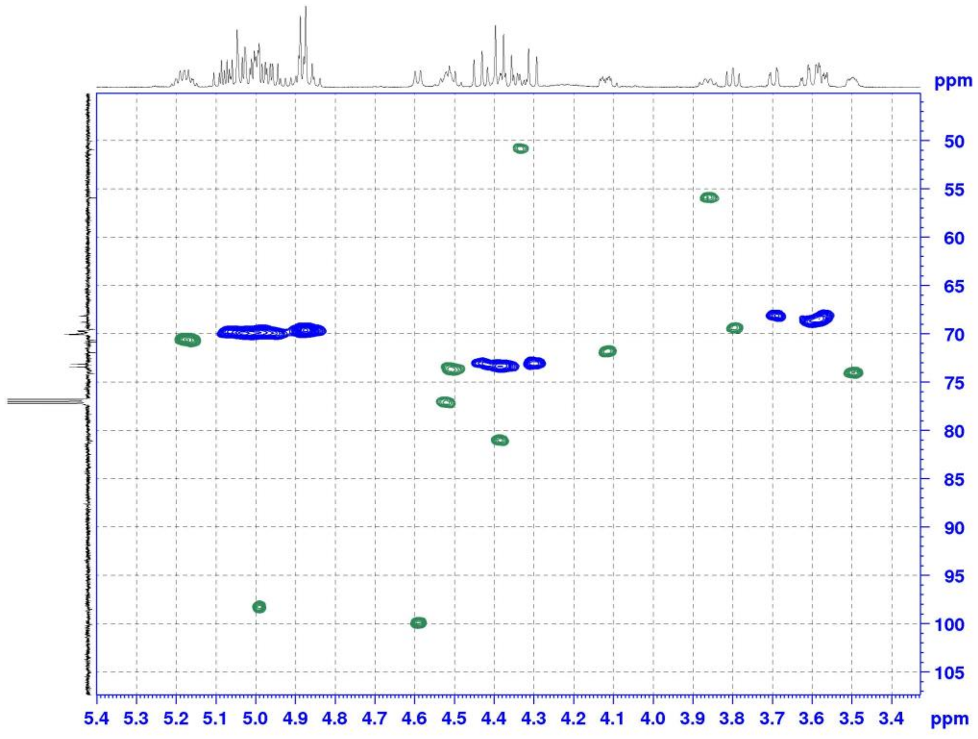

DLAM29: ^{1}H-NMR, 600 MHz, $CDCl_3$/MeOD 2:1

Due to its high amphiphilicity, the glycophospholipid tends to aggregate even in organic solutions. This is a well-known phenomenon which has a negative effect on the resolution of ^{1}H-NMR spectra. This is also the reason for providing the ^{1}H-^{13}C-HSQC NMR spectra instead of ^{13}C NMR. The rate of aggregation in chloroform-methanol solution is proportional to time, and the glycolipid in solution begins to precipitate within 5-10 h, precluding longer measurements.

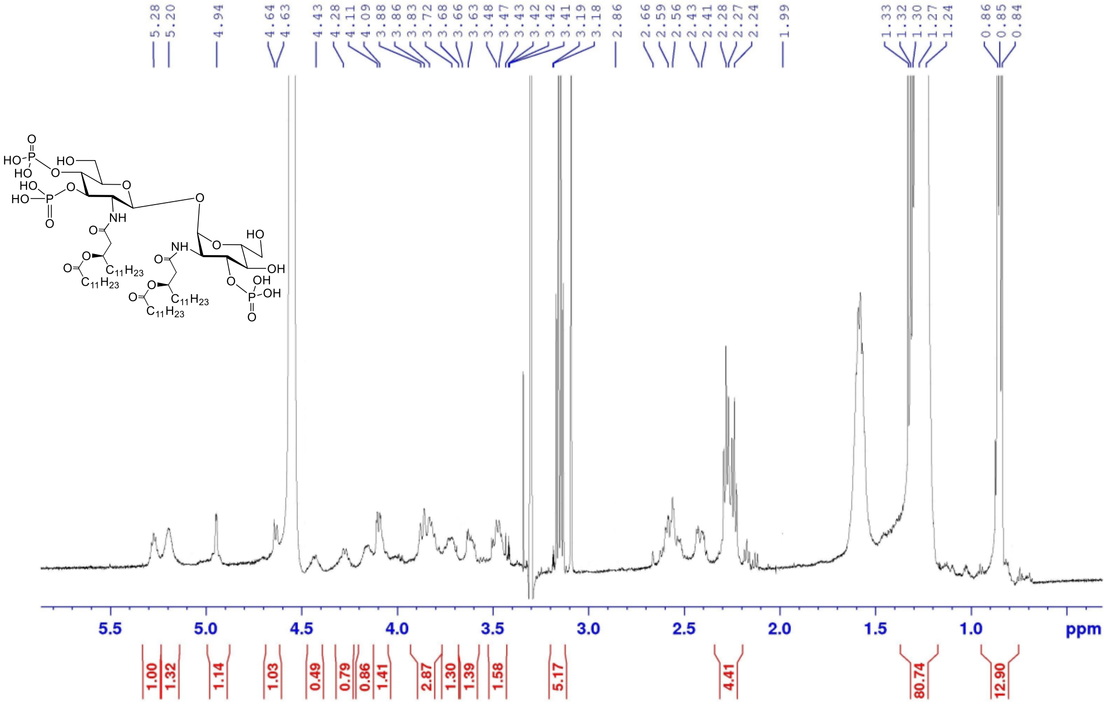

DLAM29: HSQC, $CDCl_3$/MeOD 2:1

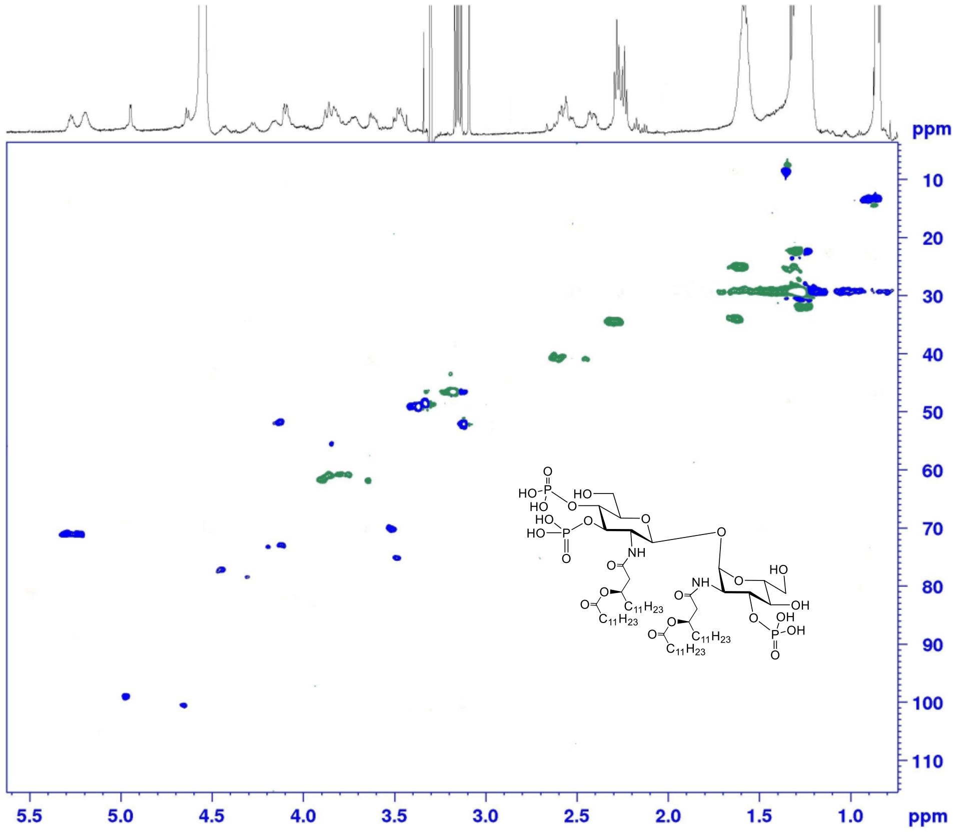

DLAM30: ^{1}H-NMR, 600 MHz, $CDCl_3$/MeOD 2:1

Due to its high amphiphilicity, the glycophospholipid tends to aggregate even in organic solutions. This is a well-known phenomenon which has a negative effect on the resolution of ^{1}H-NMR spectra. This is also the reason for providing the ^{1}H-^{13}C-HSQC NMR spectra instead of ^{13}C NMR. The rate of aggregation in choroform-methanol solution is proportional to time, and the dissoluted glycolipid begins to precipitate within 5-10 h, precluding longer measurements.

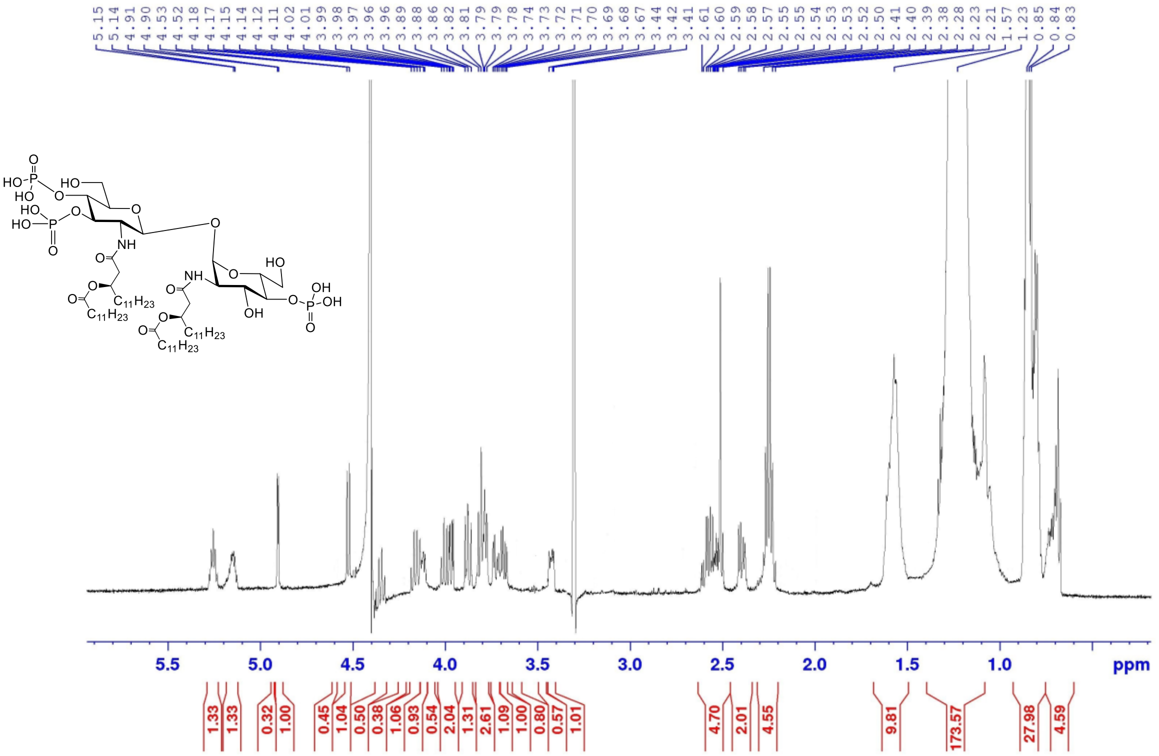

DLAM30: HSQC, $CDCl_3$/MeOD 2:1

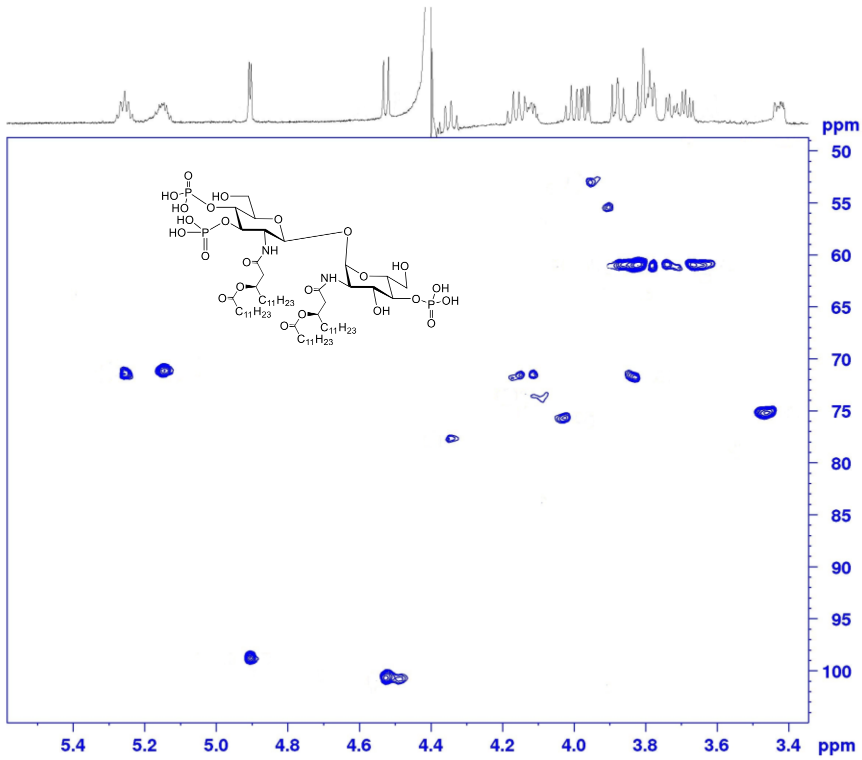

DLAM30: ^{31}P-HMBC, $CDCl_3$/MeOD 2:1

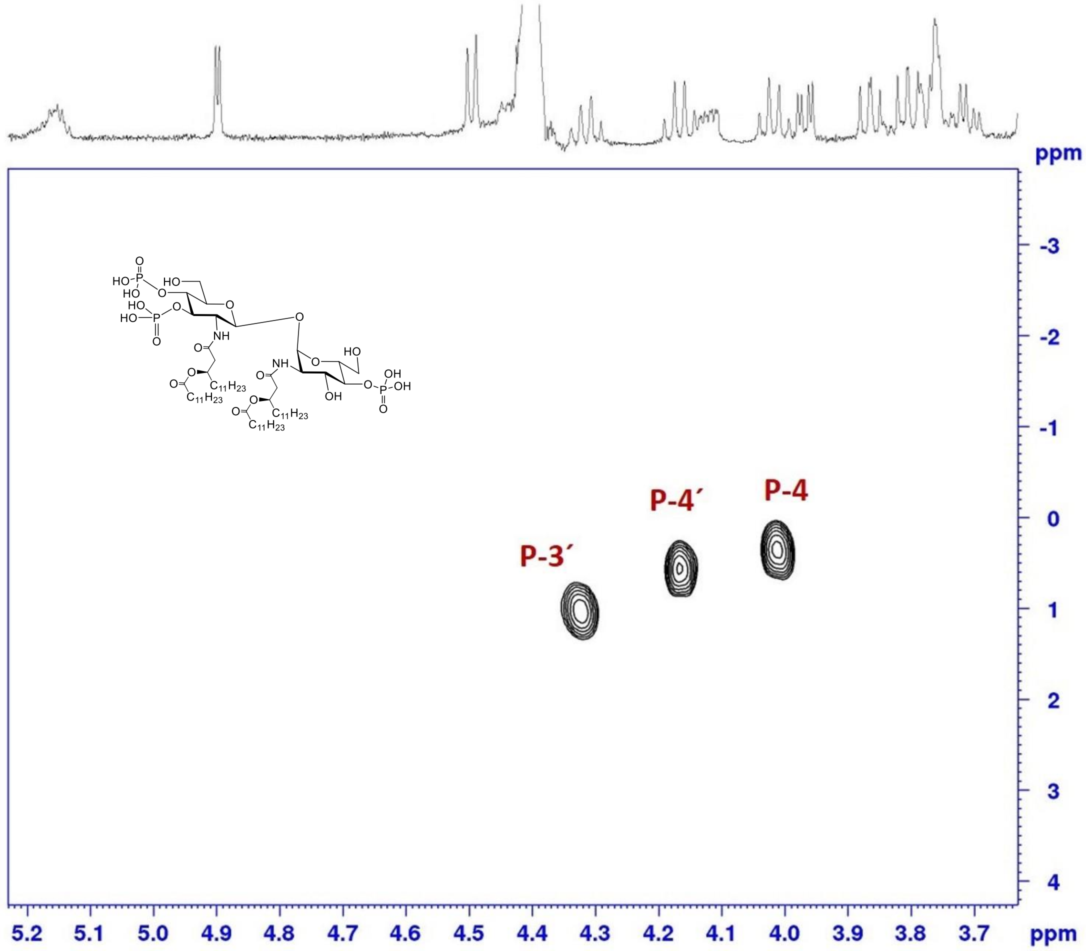

DLAM33: ^{1}H-NMR, 600 MHz, $CDCl_3$/MeOD 2:1

Due to its high amphiphilicity, the glycophospholipid tends to aggregate even in organic solutions. This is a well-known phenomenon which has a negative effect on the resolution of ^{1}H-NMR spectra. This is also the reason for providing the ^{1}H-^{13}C-HSQC NMR spectra instead of ^{13}C NMR. The rate of aggregation in choroform-methanol solution is proportional to time, and the dissoluted glycolipid begins to precipitate within 5-10 h, precluding longer measurements.

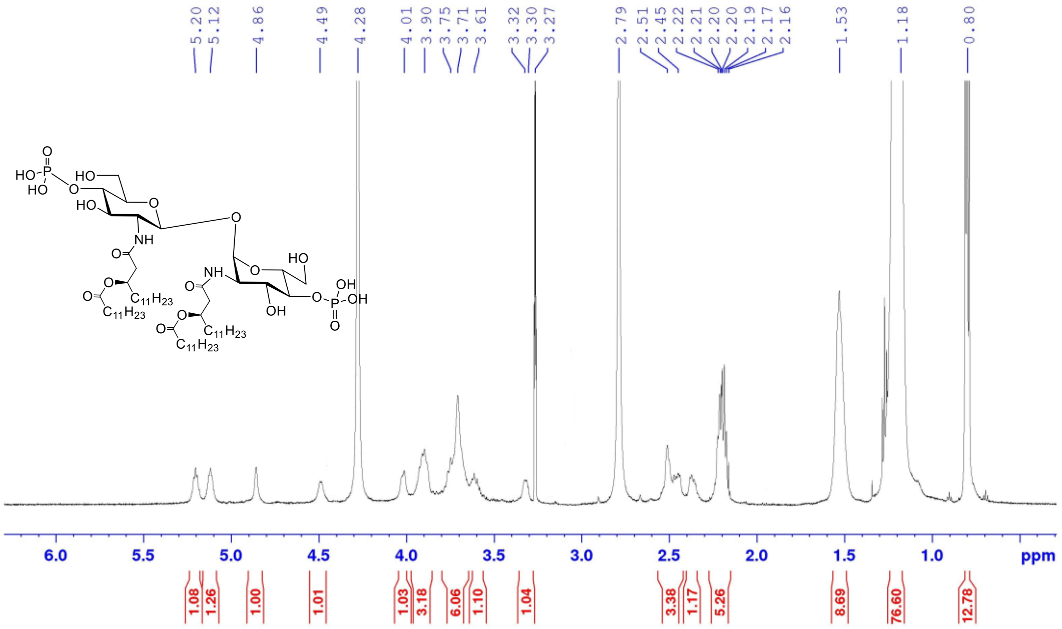

DLAM33: HSQC, $CDCl_3$/MeOD 2:1

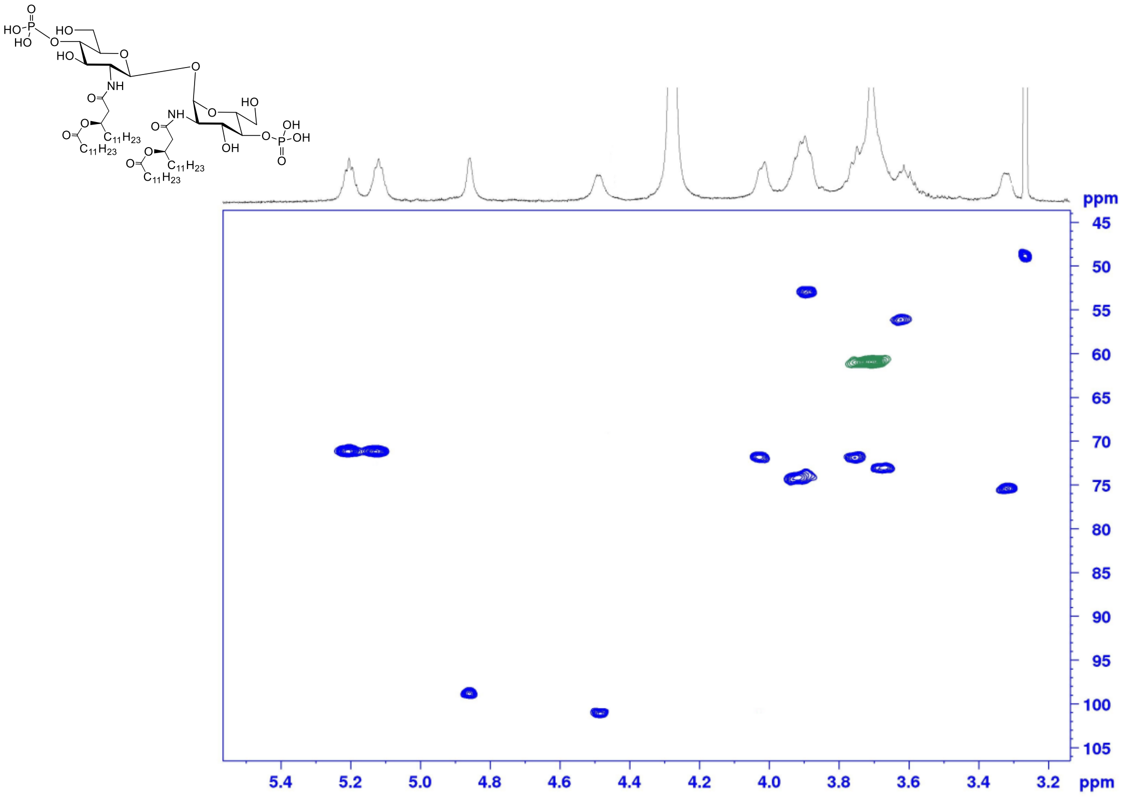

DLAM36: ^{1}H-NMR, 600 MHz, $CDCl_3$/MeOD 1:1

Due to its high amphiphilicity, the glycophospholipid tends to aggregate even in organic solutions. This is a well-known phenomenon which has a negative effect on the resolution of ^{1}H-NMR spectra.
<u>The addition of a fourth phosphate group changes the polarity,</u> allowing the amount of MeOD in solution to be increased. This changes the aggregation behaviour and allows a slightly better resolution of the ^{1}H-NMR-spectra and prevents precipitation of the glycolipid.

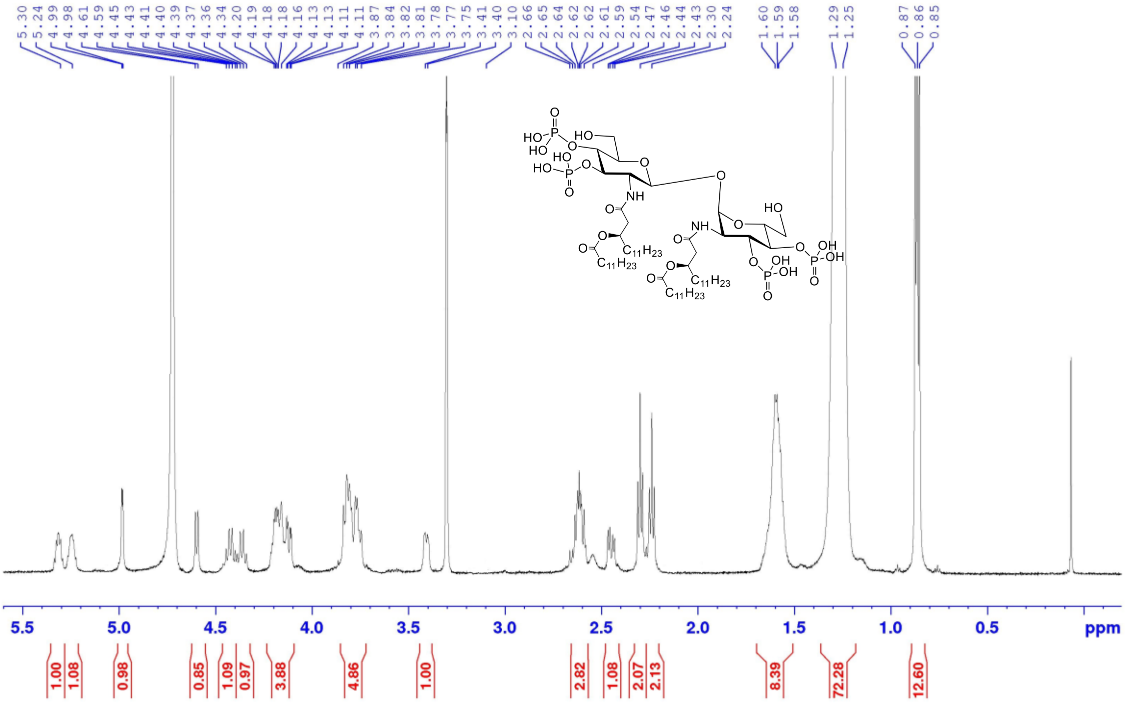

DLAM36: ^{13}C-NMR (APT), 150.9 MHz, $CDCl_3$/MeOD 1:1

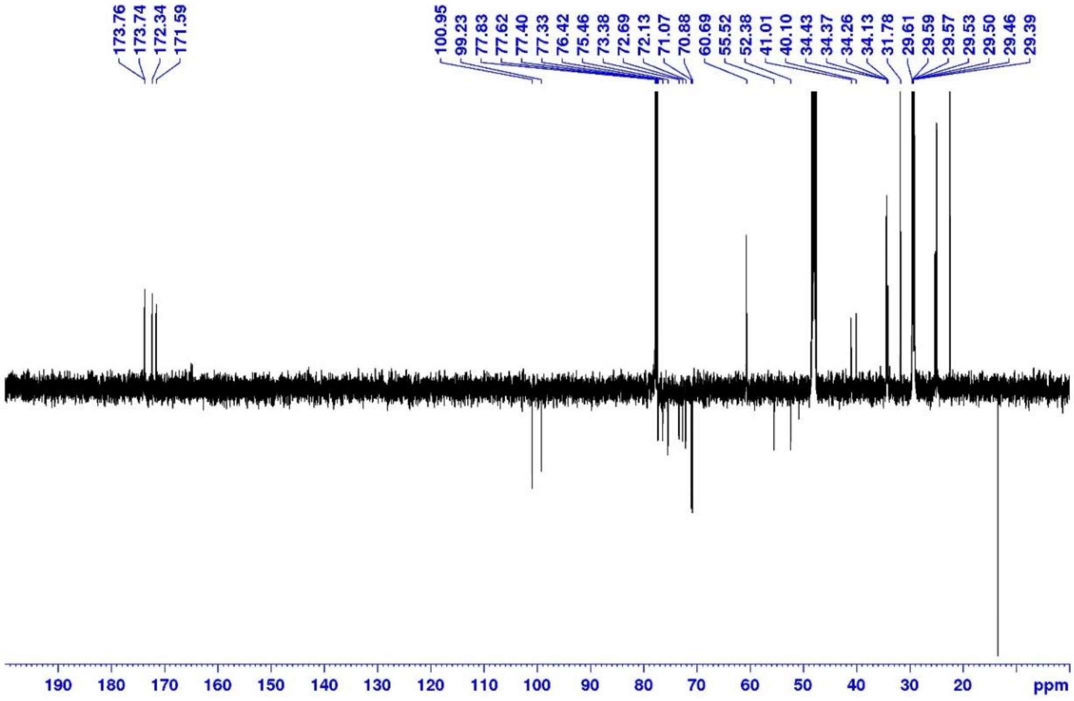

DLAM36: ^{31}P-HMBC, $CDCl_3$/MeOD 1:1

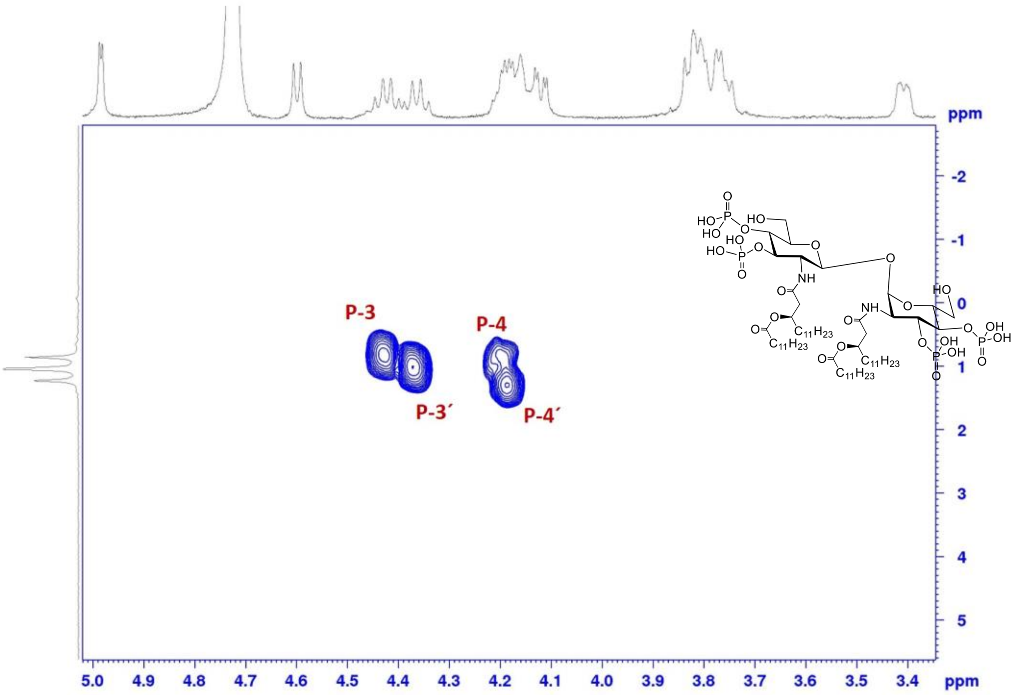

DLAM36: HSQC, $CDCl_3$/MeOD 1:1

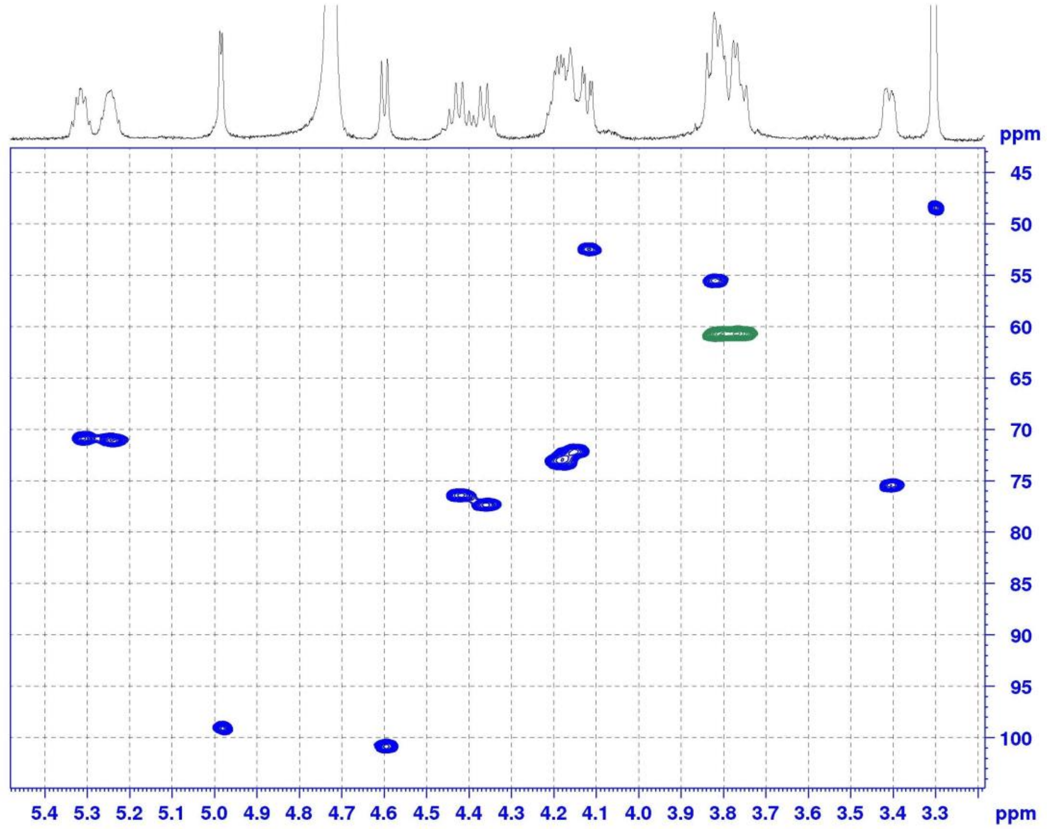

Supplement: Supplementary file 1 [file molecules-28-05948-s001.zip › molecules-2511843-supplementary.pdf]
